# Genomic evidence of functional diversity in DPANN archaea, from oxic species to anoxic vampiristic consortia

**DOI:** 10.1038/s43705-022-00088-6

**Published:** 2022-01-20

**Authors:** Adrien Vigneron, Perrine Cruaud, Connie Lovejoy, Warwick F. Vincent

**Affiliations:** 1 Département de Biologie, Université Laval, Québec, QC Canada; 2 Centre d’études nordiques (CEN), Université Laval, Québec, QC Canada; 3 Institut de Biologie Intégrative et des Systèmes, Université Laval, Québec, QC Canada; 4 Takuvik Joint International Laboratory, Université Laval, Québec, QC Canada; 5 Département de Biochimie, de Microbiologie et de Bio-informatique, Université Laval, Québec, QC Canada; 6 Québec Océan, Université Laval, Québec, QC Canada

**Keywords:** Water microbiology, Metagenomics

## Abstract

DPANN archaea account for half of the archaeal diversity of the biosphere, but with few cultivated representatives, their metabolic potential and environmental functions are poorly understood. The extreme geochemical and environmental conditions in meromictic ice-capped Lake A, in the Canadian High Arctic, provided an isolated, stratified model ecosystem to resolve the distribution and metabolism of uncultured aquatic DPANN archaea living across extreme redox and salinity gradients, from freshwater oxygenated conditions, to saline, anoxic, sulfidic waters. We recovered 28 metagenome-assembled genomes (MAGs) of DPANN archaea that provided genetic insights into their ecological function. Thiosulfate oxidation potential was detected in aerobic *Woesearchaeota*, whereas diverse metabolic functions were identified in anaerobic DPANN archaea, including degradation and fermentation of cellular compounds, and sulfide and polysulfide reduction. We also found evidence for “vampiristic” metabolism in several MAGs, with genes coding for pore-forming toxins, peptidoglycan degradation, and RNA scavenging. The vampiristic MAGs co-occurred with other DPANNs having complementary metabolic capacities, leading to the possibility that DPANN form interspecific consortia that recycle microbial carbon, nutrients and complex molecules through a DPANN archaeal shunt, adding hidden novel complexity to anaerobic microbial food webs.

## Introduction

Archaea are found across global ecosystems, carrying out diverse functions, including ammonium oxidation in aerobic environments, and sulfur, methane, and hydrocarbon cycling in anoxic environments [1–5]. As genome-centred metagenomic results become available, the size and topology of the archaeal tree of life continues to expand and includes at least 27 proposed archaeal phyla [6]. Among these, the monophyletic super-phylum DPANN, named by the initials of the first five phyla level taxa in the cluster (*Diapherotrites, Parvarchaeota, Aenigmarchaeota, Nanohaloarchaeota*, and *Nanoarchaeota* phyla), also includes *Woesearchaeota* (formerly named Deep-sea Hydrothermal Vent Euryarchaeota Group 6, DHVEG-6 [7]), *Micrarchaeota*, *Altiarchaeota* (formerly known as SM1 Euryarchaeon), *Huberarchaeota,* and *Undinarchaeota* [8, 9]. This super-phylum is estimated to account for approximately half of all the archaeal diversity of the planet [10]. DPANN Archaea have been detected in diverse environments, including subsurface aquifers [11, 12], fresh [13], marine [14] and hypersaline waters and sediments [15], hydrothermal vents [16], hot springs [17], and thawed permafrost [18, 19].

Genomic analysis of uncultured populations has increased our understanding of DPANN archaeal metabolism, and has expanded the range of known archaeal metabolic potential and putative environmental functions [6, 10, 15, 20]. In particular, metagenome-assembled genome (MAG) analysis has revealed unusual biological features within the DPANN archaea, with many incomplete or even absent metabolic pathways, suggesting a dependence on other organisms for basic metabolic requirements, and the potential for (epi)symbiotic lifestyles [10]. However, despite this recent progress exploring archaeal ecology, the biodiversity and metabolism of the DPANN super-phylum still remains largely unknown, leaving their ecosystem-level importance nebulous.

Many lakes in the Canadian High Arctic remain under ice and snow cover for most if not all the year, and some of the coastal lakes contain trapped ancient seawater. Isolated geographically and physically, these ‘meromictic’ (incompletely mixed) ecosystems are relatively pristine and free from exogenous inputs and wind mixing, leading to strong geochemical stratification of the water column. Complex and stable microbial (bacterial, archaeal, and eukaryotic) assemblages have been detected in the water columns of these lakes, with vertically segmented sulfur, nitrogen, and carbon cycles [21–24]. Among these water bodies, Lake A located on the northern coast of the Ellesmere Island is of particular interest. This lake is a marine-derived meromictic lake with an upper freshwater oxic layer, maintained by local ice and snow melt, lying above anoxic ancient ocean water that was trapped by the isostatic rebound of the continent following deglaciation [25]. With its multiple geochemically distinct layers, Lake A is a natural laboratory to investigate aquatic microbial community structure and metabolic pathways. Metagenomic analysis of the bacterial community revealed the distribution of numerous novel sulfur cycling bacteria along geochemical gradients, providing an expanded inventory of the bacterial diversity and increasing the list of known sulfur cycling bacteria and their metabolic potentials [26]. In contrast, aside from a single previous investigation of ammonium oxidizing archaeal diversity in the oxic freshwaters, the archaeal community of Lake A and other high-Arctic lakes has been poorly explored and remains uncharacterized [21, 23].

Here, we applied DNA and cDNA-based 16 S rRNA gene sequencing, quantitative PCR (qPCR), and genome-centric metagenomics to analyze archaeal community composition and function along the salinity, oxygen and sulfur gradients of Lake A. DPANN archaea including *Woesearchaeota*, contributed a large proportion of the microbial community in the metagenomic datasets, notably in the anoxic and sulfidic marine waters. Multiple MAGs of various archaeal populations were recovered, providing the first High Arctic MAGs of DPANN representatives. Analyzing the genetic composition of these DPANN archaea MAGs, we elucidated their metabolic capabilities to better understand their distribution across the contrasting environmental conditions of this model microbial ecosystem, and to provide broader insights into the metabolic diversity of this archaeal super-phylum.

## Material and methods

### Sample collection and nucleic acid extraction

**Fig. 1:**
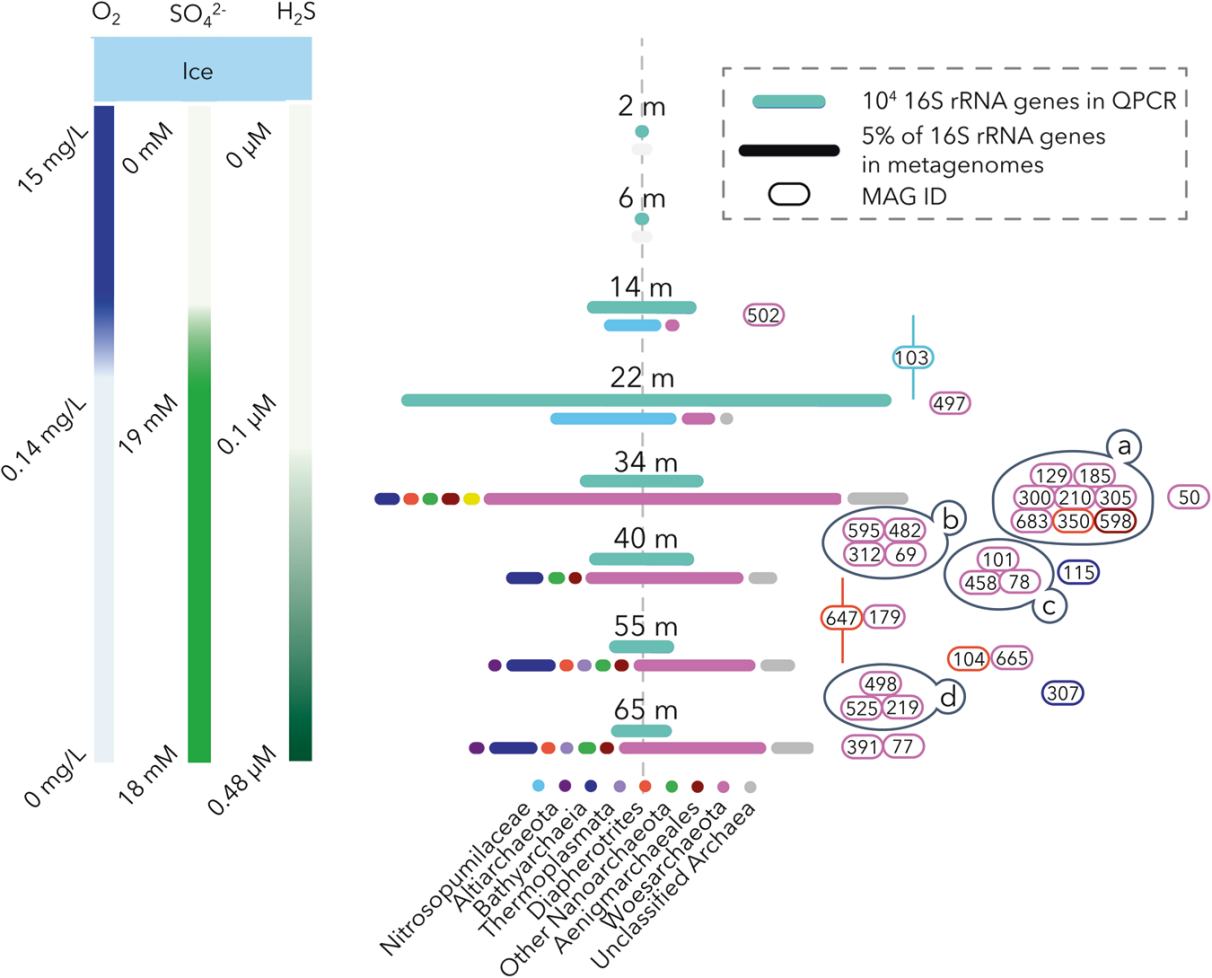
Archaeal community composition and abundance. Left-hand panel shows depth of samples along the geochemical gradients of Lake A. The right-hand panel shows the total archaeal abundance (top cyan bar), with the length of the bar based on the number of 16 S rRNA genes detected by qPCR (see legend for a 10^4^ unit). Below this bar is the archaeal community composition, shown as colored bars, with the length of each bar based on to the relative proportion of 16 S rRNA genes identified in the metagenomes; the 5% scale bar is given in the legend. Circled numbers indicate the MAG ID and color of the circle indicates the taxonomy of the MAGs from the bottom legend. MAGs are placed at the depth of highest coverage. The horizontal line spanning 55 and 40 m for bin103 and bin647 indicates that these MAGs were recovered in similar proportions from those depths. Clusters (a,b,c,d) of distributions indicate highly correlated MAGs (*R* > 0.99, *p* < 0.0001). Detailed geochemical data for Lake A has been previously published [25, 26] and is available in the Nordicana D database (https://www.cen.ulaval.ca/nordicanad).

Lake A is a salinity-stratified, ice-capped lake located in a polar desert catchment on northern Ellesmere Island, Nunavut, Canada that has been the site of several geochemical investigations [25, 27]. For this study, samples were collected in summer 2017 (18 July) near the center of the lake (lat. 82° 59.667’ N, long. 75° 26.602’ W) from three separate 24 cm-diameter holes drilled through the 0.6 m thick ice cover. Oxygen concentrations, salinity, and temperature profiles were measured using a XR-420 CTD profiler (RBR Ltd., Ottawa, Canada) to 65 m, which was the length of the instrument line, to rapidly visualize the boundary between the oxic freshwater and the marine sulfidic and anoxic waters. Eight sampling depths (2, 6, 14, 22, 34, 40, 55, and 65 m) were selected to cover water layers identified from the CTD profiles (Fig. 1). The sampling strategy and experimental procedures used in this study were detailed previously in the bacterial survey of Lake A [26]. Briefly, for each selected depth, 1 L of each triplicate water sample, collected with a Limnos Water sampler (KC, Denmark), was directly filtered through separate 0.22 µm pore size Sterivex filters^TM^ (Merck Millipore) and then stored below −50 °C until nucleic acid extraction.

Nucleic acids (DNA and RNA) of two of the replicate samples per depth were extracted from the same Sterivex filters using Qiagen Allprep DNA/RNA Mini Kit (Qiagen, Hilden, Germany) as previously described [28]. Negative controls of nucleic acid extractions, where no template was introduced at the initial step, were simultaneously carried out, to identify potential contamination from extraction kits, plasticware or reagents. The DNA extracts were stored at −20 °C until library preparation. For RNA extracts, two additional DNase steps (DNase I; Ambion, Foster City, CA, USA) were used to remove any trace of carried over DNA. The absence of DNA contamination was confirmed by amplification of 16 S rRNA genes with bacterial primers using the RNA extracts (undiluted and diluted ten times) as template, with no product detected after 35 PCR cycles. The extracted RNA was immediately converted to cDNA using a High-Capacity cDNA Reverse Transcription kit (Applied Biosystems, Foster City, CA, USA) and stored as cDNA at −20 °C until library preparation.

### Quantitative PCR

The abundance of archaeal 16 S rRNA genes was estimated in two replicate samples per depth using qPCR with primers Arc787f/Arc1059r [29]. Quantification was performed in triplicate with a range of template concentrations (0.1, 0.5, 1 ng of DNA) to compensate for any PCR inhibition. Genomic DNA extracted from *Methanosarcina acetivorans* (DSM2834) were serially diluted to construct standard curves (concentrations ranged from 10^2^ to 10^6^ 16 S rRNA genes per reaction). The *R*^2^ of standard curves obtained by qPCR were above 0.99, and PCR efficiencies were above 88.7%. The qPCR results were expressed in terms of 16 S rRNA gene number per mL of water sample (Fig. 1).

### Illumina MiSeq amplicon library preparation, sequencing and analysis

Archaeal community composition of by way of amplicons was determined by high throughput sequencing of 16 S rRNA (cDNA) and 16 S rRNA genes (DNA) using primers targeting the archaeal V1-V3 region (A27F/Arc518R; 500 bp) [7]. All PCR reactions were carried out following established protocols [30]. Samples and negative controls of the nucleic acids extractions, transcription and PCRs were sequenced using an Illumina MiSeq v3 kit at the IBIS/Laval University, Plate-forme d’Analyses Génomiques (Québec, QC). Reads were assembled into single paired-end sequences, curated and clustered into OTUs (97% sequence similarity) as detailed in a GitHub repository (https://github.com/CruaudPe/MiSeq_Multigenique). OTUs detected in negative controls were removed from the analysis as described in [31]. Taxonomic affiliations of the reads were determined with mothur v.1.44.3 [32] using BLAST against Silva database release 138 as a reference [33].

### Metagenomic library preparation, sequencing and analysis

One metagenome per sample depth, for a total of eight metagenomes, was constructed using a Nextera XT Library Kit (Illumina, San Diego, CA, USA). The eight metagenomes were pooled in equimolar quantities then sequenced in two Illumina MiSeq (2 × 300 bp) runs and one Illumina NextSeq run (2 × 150 bp) at the Institut de Biologie Integrative et des Systèmes (IBIS) sequencing platform (Université Laval, Canada) and at the CGEB—Integrated Microbiome Resource (Dalhousie University, Canada), respectively. Datasets (MiSeq and NextSeq) were pooled then quality filtered using the Trimmomatic v.0.39 tool [34], with default settings. The 16 S rRNA reads longer than 110 bp were isolated from metagenomic reads using REAGO 1.1 [35], and taxonomic assignments were performed as for the 16 S rRNA gene amplicons.

Each metagenome was assembled separately from paired-end reads passing quality filtering using SPAdes v3.15.1 [36]. Assembled contigs and mapping files (BAM files generated using BBmap 38.86) were uploaded to the Department of Energy Joint Genome Institute (DOE-JGI) IMG/MER analysis pipeline [37] for gene calling (Prodigal v2.6.3) and functional annotation using HMMER 3.1b2 against KEGG, Rfam 12.0, COG 2003, Pfam v30 and IMG-NR 20181114 databases. To account for differences in sequencing depth between samples, metagenomes were normalized to the size of the smallest dataset.

### Binning and functional characterization

For metagenome assembled genome (MAG) reconstruction, all quality filtered sequences were pooled and co-assembled using MEGAHIT v.1.2.9 [38]. Read coverage of the contigs was carried out using bwa-mem v.0.7.17 (http://bio-bwa.sourceforge.net), followed by contig binning using MetaBAT-2 [39] with contigs longer than 2000 bp. The completeness and contamination level of the combined genomic bins were then evaluated using CheckM v.1.1.2 [40]. Only bins with a contamination level under 5% and completeness above 40% were analyzed. The genetic composition of genomic bins was then explored using KEGG [41] and KofamKOALA. In addition, we searched for carbohydrate-active enzymes using dbCAN2 [42] and the CAZy database [43]. The phylogeny of MAGS was determined using the GTDB_Tk classify workflow with genomes containing a minimum of 40% of the 122 archaeal marker genes[44]. In addition, a phylogenetic tree of all ribosomal protein genes (rpL2, 3, 4, 5, 6, 14, 15, 16, 18, 22, 24, rpS3, 6, 8, 10, 17, 19) detected in the bins was constructed using metabolisHMM [45]. Publicly available DPANN archaeon genomes were downloaded from NCBI database and after applying the same completeness and contamination filtering as in our genomic bins, 100 public draft genomes of DPANN archaea were included in the phylogenetic analysis. Representatives of other archaeal phylum (*n* = 50) were also downloaded and included in the analysis. In addition, a phylogenetic tree of the pore-forming toxin was constructed based on representative sequences of the hemolysin gene (*tlyC*; K03699) downloaded from NCBI. The NCBI sequences were then aligned with sequences from our genomic bins using Clustal Omega [46] and a phylogenetic tree was constructed using IQ-Tree v.1.6.12 [47] with LG + F + G4 model and 1000 bootstraps; the results were then visualized and edited using iTOL. [48].

### Correlation analysis

Depth distribution and relative abundance of the genomic bins were inferred from the average coverage of the genomic bin per sampled depths. A Pearson’s correlation matrix was then calculated using the depth distribution of all quality filtered genomic bins. Genomic bins with correlation coefficients of *R*^2^ > 0.99 and *p* value < 0.0001 with DPANN archaea were isolated and analyzed in detail. The results were visualized in a network using the software environment R (v.4.0.3) implemented with the igraph (v1.2.6) package [49].

## Results

### Archaea and Woesearchaeota community composition of Lake A

The Lake A water column was sampled in triplicate at eight selected depths (2, 6, 14, 22, 34, 40, 55, and 65 m) to cover oxygen, salinity, and sulfur gradients of the lake (Fig. 1). Archaeal community composition was investigated by DNA and cDNA-based 16 S rRNA amplicon sequencing, qPCR and primer-free metagenomic sequencing on the discrete samples collected down the water column. Based on qPCR and the metagenomic dataset, Archaea represented <1% of the microbial community in the freshwater layer of the lake (Fig. 1). A peak of archaeal abundance was detected at the oxycline by qPCR, where metagenomic and DNA and cDNA-based 16 S rRNA sequences were mainly affiliated to *Nitrosopumilaceae*. *Woesearchaeota* sequences were also detected at the oxycline but only in the metagenomic dataset (16% of the archaeal metagenomic 16 S rRNA reads), revealing the occurrence of woesearchaeotal lineages with 16 S rRNA genes that escaped primer-based detection (Fig. 1 and Supplementary Figure). In the marine anoxic strata, qPCR data indicated a lower archaeal abundance than at the oxycline, however archaeal sequences represented up to 23% of the total 16 S rRNA genes in metagenomes and covered a larger taxonomic diversity (Fig. 1). These archaeal metagenomic 16 S rRNA sequences were mainly affiliated to *Woesearchaeota* (up to 77% of the archaeal reads) and *Bathyarchaeota* (up to 19% of the archaeal reads). Both phyla were also detected by the DNA and cDNA-based amplicon sequencing approaches but in lower proportions (Fig. 1, Supplementary Fig. 1), probably due to primer mismatches. By contrast, 16 S rRNA genes related to *Methanofastidiosales* and *Thermoplasmatales*, detected by amplicon sequencing were rare in the metagenomes (Fig. 1, Supplementary Fig. 1). These discrepancies between results of amplicon and metagenomic sequencing are likely due to primer bias precluding an accurate representation of the microbial community. Finally, besides the *Woesearchaeota*, other DPANN archaea, including *Aenigmarchaeales* and *Diapherotrites* were detected by amplicon and metagenomic sequencing in the anoxic waters of marine origin (Fig. 1).

### Phylogeny of metagenome assembled DPANN and Woesearchaeota genomes

**Fig. 2:**
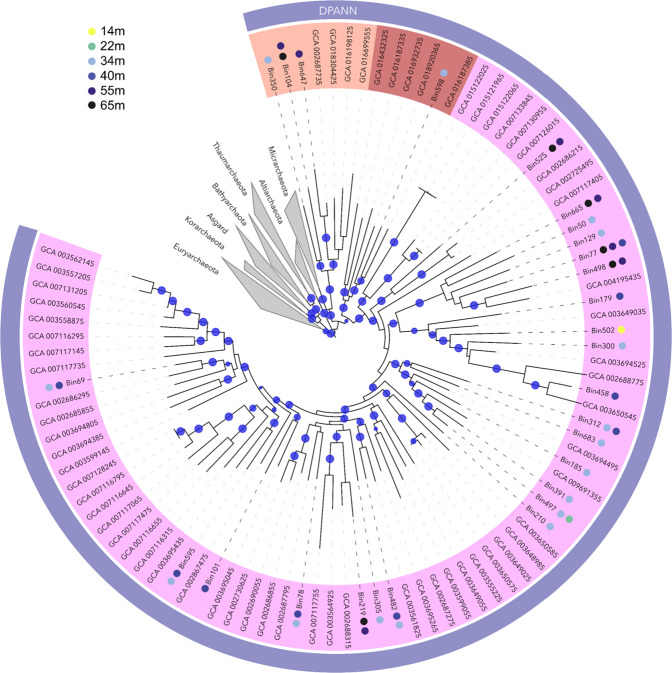
Phylogenetic tree of DPANN archaea. The tree was constructed based on concatenated ribosomal protein genes alignment with genomes available on NCBI. The blue points in the branches of the tree represent bootstrap values >0.8. The colored dot next to the bin name indicates the sampling depth where the MAGs were recovered. The major groups are color coded with *Woeseachaeota* in mauve, *Diapherotrites* in orange and *Aenigmarchaeota* in brown. The alignment file is available in Supplementary Material 1.

After binning of the contigs, a total of 30 medium- to good-quality (defined here as <5% contamination and >40% complete) archaeal MAGs were recovered. Taxonomic affiliation of MAGS was inferred from the 16 S rRNA gene analysis, GTDB_Tk topological placement and average nucleotide identity (ANI) and the phylogenetic tree of the concatenated alignment of ribosomal protein genes (rpL2, 3, 4, 5, 6, 14, 15, 16, 18, 22, 24, rpS3, 6, 8, 10, 17, 19) found in the MAGs. The different taxonomic affiliation strategies were congruent and most of archaeal MAGs were affiliated to the DPANN super-phylum, including 18 *Woesearchaeota* MAGs, 6 *Pacearchaeota* MAGs, 3 *Diapherotrites* MAGs and 1 *Aenigmarchaeota* MAG. Non-DPANN archaeal MAGs were related to *Bathyarchaeota* (2 MAGs) and *Nitrosopumilus* (1 MAG) (Fig. 2). Although multigene phylogenetic analyses are currently limited due to the restricted number of genomes available, our phylogenetic tree, constructed using available ribosomal protein genes indicated that Lake A *Woesearchaeota* were taxonomically diverse and widely distributed within known *Woesearchaeota* groups (Fig. 2).

### Depth distribution of DPANN and Woesearchaeota MAGs

**Fig. 3:**
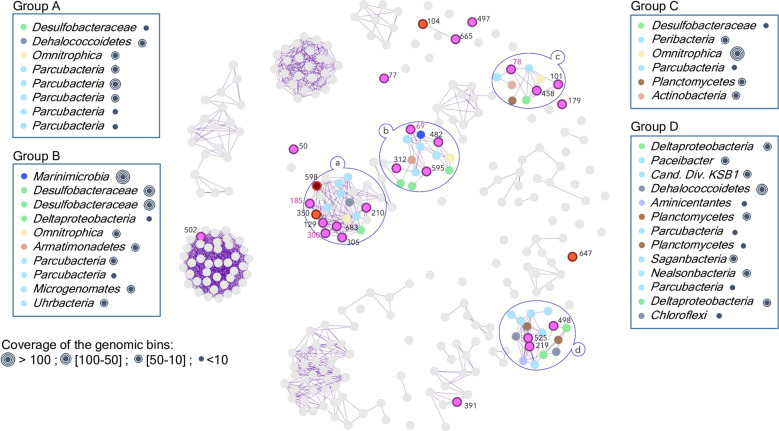
Network of the microbial (bacterial and archaeal) MAGs based on the correlation matrix calculated with the average coverage per sampled depth. MAGs of *Woesearchaeota* (pink bold dots), *Aenigmarchaeota* (brown bold dot) and *Diapherotrites* (orange bold dots) are color coded and labelled as in Fig. 1. Bacterial MAGs are as gray dots except for the highly correlated MAGs that are color coded by phyla. The purple lines connect MAGs with correlation coefficients (R) > 0.99.

**Fig. 4:**
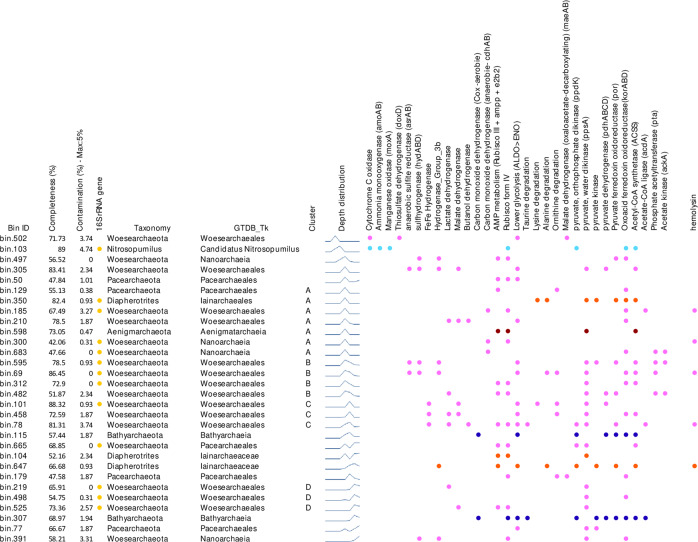
Metabolic potentials identified in the archaeal MAGs of Lake A. The MAGs are arranged by depth of maximum occurrence, and membership of clusters from the groups in Fig. 3 is indicated. Completeness and contamination percentages were estimated using CheckM. Discrepancies of the archaeal taxonomy are explained by differential terminology between the genome taxonomy database (GTDB) and NCBI with DPANN archaea and *Diapherotrites* referenced as *Nanoarchaeia* and *Ianarchaeales* respectively in GTDB.

Only one *Woesearchaeota* MAG was recovered from the upper oxic freshwater layer (Bin 502), while another MAG (Bin 497) was recovered from the oxycline (Figs. 1 and 2). All the other DPANN and woesearchaeotal MAGs were recovered from anoxic saline waters. However, the depth distribution of these MAGs differed among lineages. The majority of the MAGs fell within four clusters, and strongly covaried within the clusters (*R*^2^ > 0.99, *p* < 0.0001). Eight MAGs were detected exclusively at the oxycline/anoxic water transition (34 m; marked as A in Figs. 1, 3 and 4). Four MAGs were detected at 34 m and at the sulfate-rich layer of the marine anoxic waters (34–40 m; marked as B in Figs. 1, 3 and 4). Three were found exclusively at 40 m (marked as C). Finally, three co-correlated MAGs were detected only at the bottom of the marine water column (55 and 65 m; marked as D in Figs. 1, 3 and 4).

### Correlations of DPANN archaea with bacterial populations

A correlation matrix including bacterial MAGs recovered from the same samples [26] was calculated to investigate bacterial interactions with the DPANN archaeal clusters. Due to the low sample size, only correlations with *R*^2^ > 0.99 and *p* value < 0.0001 were kept as indicators of potential interactions (purple edges in Fig. 3). Bacterial MAGs strongly correlated with DPANN clusters were mainly affiliated to taxa in the Candidate Phyla Radiation, including *Parcubacteria*, *Deltaproteobacteria*, *Omnitrophica*, and *Planctomycetes*. However, considering the coverage of the MAGs as a proxy for the relative abundance of their populations, the most predominant populations correlated with DPANN clusters belonged to *Marinimicrobia* and *Omnitrophica* recovered from the 34 to 40 m samples (Fig. 3).

### Metabolic potential of metagenome assembled DPANN and *Woesearchaota* genomes

Our MAG analysis of the 19 *Woesearchaeota* and the 8 other DPANN archaea (*Diapherotrites*, *Pacearchaeota* and *Aenigmarchaeota*) indicated a relatively limited known metabolic potential (Fig. 4), with on average 352 KEGG orthologues (KO) over 1175 protein-coding genes identified per genome (30%). For comparison, an average of 845 KOs over 1907 protein-coding genes (44%) were identified in our *Bathyarchaeota* and *Nitrosopumilus* MAGs (Supplementary Table 1).

The metabolic core of the *Woesearchaeota*, defined as genes present in at least 75% of the *Woesearchaeota* MAGs, was limited to only 158 shared KOs, including housekeeping genes, ribosomal protein genes, transport systems and archaeal flagella genes. By contrast up to 737 KOs were only detected in four or fewer MAGs, suggesting high variability in metabolic processes among *Woesearchaeota*, albeit with the caveat that some genes might not have been detected due to the incompleteness of our MAGs. The non-core metabolic processes differed by environment and water layer of Lake A. For example, aerobic metabolism, with a gene coding for thiosulfate:quinone oxidoreductase (*doxD*), involved in sulfur/thiosulfate oxidation, was detected in the *Woesearchaeota* MAG recovered from the oxic freshwaters (Bin 502). This MAG also included genes for cytochrome c oxidase, supporting aerobic respiration. By contrast, anaerobic pathways were identified in the 18 other *Woesearchaeota* as well as in all other DPANN MAGs. Genes for anaerobic sulfite reduction (*asrAB*) were detected in two *Woesearchaeota* MAGs from cluster B and one from the oxycline in cluster A (Fig. 4). These MAGs, as well as two additional *Woesearchaeota* MAGs from those clusters had genes coding for sulfhydrogenase (*hydABD*), which is involved in elemental sulfur/polysulfide reduction (Fig. 4).

All woesearchaeotal and other DPANN MAGs lacked evidence for a complete tricarboxylic acid cycle and pentose phosphate pathway, and only the end of glycolysis was identified in half of the *Woesearchaeota* MAGs. A complete adenosine monophosphate (AMP) salvage pathway, including the gene coding for type III ribulose-1,5-bisphosphate carboxylase (Rubisco) was detected in 11 DPANN MAGs affiliated to *Diapherotrites* (1 MAG), *Aenigmarchaeota* (1 MAG), *Pacearchaeota* (3 MAGs), and *Woesearchaeota* (6 MAGs) that were recovered from the anoxic saline water (Fig. 3). The potential to recycle some amino acids (taurine, lysine, alanine, or ornithine), through conversion of amino acids to two-oxoacids by transaminases and subsequent decarboxylation to acyl-CoA via pyruvate/2-oxoacid:ferredoxin oxidoreductase was found in a small number of MAGs (5 MAGs of *Woesarchaeota* and 1 of *Diapherotrites*). While genes converting phosphoenolpyruvate generated via the AMP salvage pathway or glycolysis into pyruvate (*pps*) were detected in most (17 MAGs, 63%) of the DPANN MAGs. Genes coding for lactate, malate and butanol dehydrogenases, providing MAGs with a pathway for pyruvate fermentation, were detected in eight *Woesearchaeota* MAGs, and genes for hydrogenases (FeFe hydrogenases and type 3b hydrogenases) were also detected in nine MAGs, indicating that hydrogen, lactate, malate and butanol may be end-products of *Woesearchaeota* metabolism (Fig. 4). Acetate metabolism (consumption or production), through acetyl-CoA synthetase, acetate-CoA ligase (*acdA*) or the acetate kinase (*pta/ackA*) pathway was also identified in 13 DPANN MAGs. Finally, three *Woesearchaeota* MAGs included genes for anaerobic carbon monoxide dehydrogenase (*cdhAB*).

### Genes for carbohydrate activating enzymes of the Woesearchaeota

**Fig. 5:**
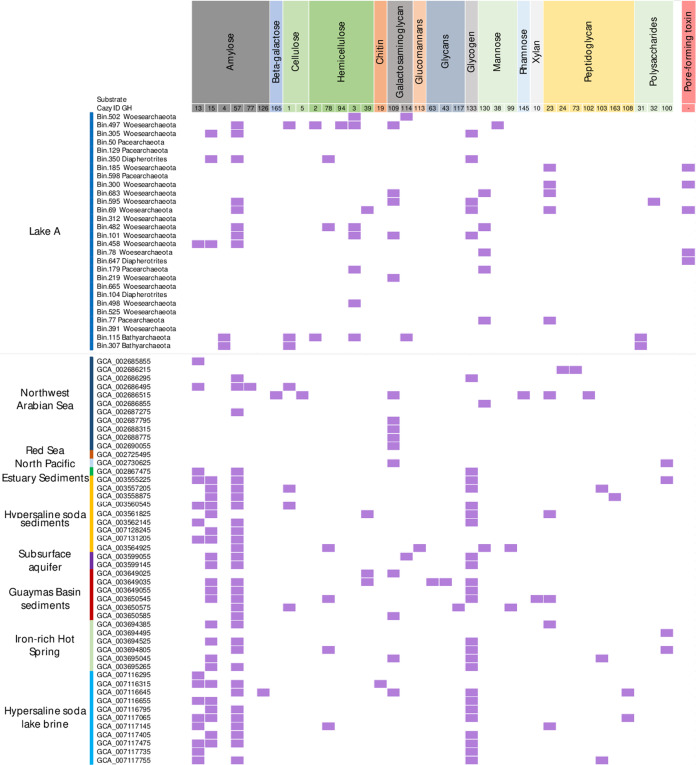
Genes for carbohydrate activating enzymes identified in the DPANN archaea MAGs of Lake A and in public databases. MAGs of Lake A are arranged by depth. Two additional *Bathyarchaeota* MAGs from Lake A were added for comparison.

The number of genes for carbohydrate activating enzymes (CAZy) identified was limited and uneven in Lake A DPANN MAGs, with a maximum of seven CAZy genes per MAG. More CAZy genes were detected in *Woesearchaeota* than in the other DPANN MAGs (Fig. 5). The genetic potential to degrade amylose, (hemi)cellulose, glycogen, glycan, polysaccharides was detected in the DPANN MAGs. To gauge the diversity of CAZy genes, we compared the distribution of CAZy genes in Lake A with other available DPANN MAGs (Fig. 5). CAZy genes for glycogen degradation were present in 23% of Lake A vs. 50% of Woesearchaeotal MAGs from other environments. Likewise, CAZy genes for amylose degradation were detected in 38% of Lake A MAGs vs. 67% for the other MAGs. By contrast, the potential for hemicellulose and polysaccharide degradation was overrepresented in Lake A MAGs compared to the other Woesearchaeotal genomes available. Finally, genes for peptidoglycan degradation, previously reported in a subsurface aquifer woesearchaeotal MAG, were detected in five woesearchaeotal MAGs of Lake A (Bin 300, Bin 185, Bin 77, Bin 683, and Bin 69), including the two most abundant woesearchaeotal MAGs (Bins 683 and 69) (Fig. 5).

### Genes of a pore-forming toxin in DPANN archaea

**Fig. 6:**
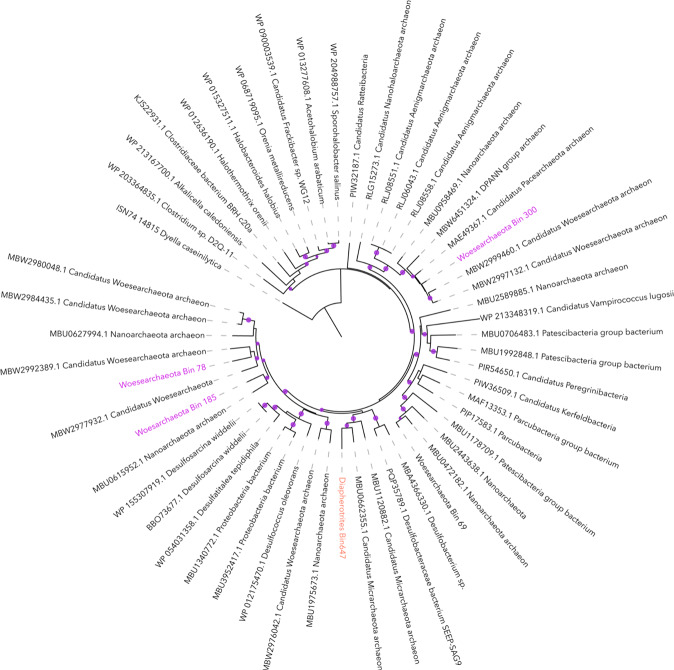
Phylogenetic tree of the pore-forming toxin gene. The tree was constructed with publicly available sequences of the pore-forming toxin gene (*tlyC*; K03699) available on NCBI. Blue points on the branches represent bootstrap values > 0.7. Sequences recovered from Lake A are color coded according to their taxonomic affiliation.

A gene coding for a putative pore-forming toxin (K03699) was detected in three of the peptidoglycan degrading Woesearchaeotal MAGs (Bin 300, Bin 185, and Bin 69) as well as in Bin 78 and *Diapherotrites* Bin 647 (Fig. 4). Comparison with public databases and phylogenetic analysis of the recovered sequences indicated that the sequence of the pore-forming toxin shared homologies with the membrane-damaging toxins found in *Clostridiales* and in Candidate Phyla Radiation bacteria including *Vampirococcus lugosii* (Fig. 6). Furthermore, the analysis also revealed that various other DPANN archaea genomes included similar genes encoding for pore-forming toxins, supporting the occurrence of such a gene in the Lake A bins.

## Discussion

The pronounced geochemical gradients down the water column of the ice-capped Lake A makes it an outstanding model system to explore the microbial metabolism of uncultivated groups over a broad set of environmental domains. For example, the depth distribution pattern of the *Nitrosopumilus*-related lineage along oxygen and ammonia gradients of Lake A and the detection of *amoAB* genes in its MAG corroborates the aerobic ammonia oxidation metabolism of *Nitrosopumilus* relatives that has been previously reported from clone library and qPCR data [23]. DPANN archaea, including *Woesearchaeota*, were not found in the previous PCR-based investigations of the Lake A archaeal community [21, 23]. However, our metagenomic approach indicated that these Archaea were major components of the microbial community, most notably in the anoxic ancient marine waters, suggesting that they are important contributors to ecosystem functioning.

### Metabolic variability within *Woesearchaeota* members

The recovery of up to 30 MAGs affiliated to *Woesearchaeota* and other DPANN archaea, indicated that genetically distinct populations occurred at the different sample depths of High Arctic Lake A. Although DPANN MAGs including *Woesearchaeota* have been detected previously in Arctic-Boreal lakes [50] and lakes associated with thawing permafrost [19, 51], these Lake A observations are, to our knowledge, the first DPANN MAGs from such a high latitude environment. Our phylogenetic analysis revealed that the woesearchaeotal populations were taxonomically diverse within the phyla radiation (Fig. 2). Although primer bias precluded the detection of all *Woesearchaeota* lineages by amplicon-based approaches, detection of *Woesearchaeota* in our cDNA results supported the likelihood of in situ activity for at least the detectable part of these populations.

Oxygen has been proposed as a strong determining factor for Woesearchaeotal diversity [52]. However, given the limited number of genomes and MAGs recovered from aerobic *Woesearchaeota*, the genetic capabilities of aerobic lineages remained speculative. We recovered one *Woesearchaeota* MAG from the oxic freshwater layer of Lake A. This MAG included genes for cytochrome oxidase and thiosulfate oxidation potential, supporting an aerobic metabolism. This result suggests that some aerobic *Woesearchaeota* are involved in the biogeochemical sulfur cycle by oxidizing sulfur cycle intermediates produced under anoxic and microoxic conditions.

Based on their genetic potential, part of the *Woesearchaeota* anaerobic lineages may likely be involved in other aspects of sulfur cycling. Genes for sulfide reduction and polysulfide reduction were identified in a few MAGs, supporting similar findings in hypersaline environments [15] and hypoxic marine waters [53]. This result indicates the potential for some *Woesearchaeota* to gain energy from reduced sulfur intermediates under anoxic and sulfidic conditions.

In addition to sulfur cycling, the genetic potential identified in *Woesearchaeota* and other DPANN archaea was centred on pyruvate metabolism and fermentation with acetate, lactate or hydrogen as end-products, as reported earlier [10]. The potential for ribose degradation through the AMP salvage pathway was detected in multiple MAGs, confirming previous genomic explorations of DPANN archaea [11, 54] and suggesting a potential RNA scavenging-based metabolism. The potential to degrade amino acids and various carbohydrates was also identified across the *Woesearchaeota* and other DPANN MAGs. With up to 17 different genes coding for carbohydrate degrading enzymes, the catabolic variability of *Woesearchaeota* was larger in Lake A compared to other environments where woesearchaeotal MAGs have been recovered (Guaymas sediments, a subsurface aquifer and soda lakes; Fig. 5), reflecting the contemporaneous co-occurrence of diverse habitats down the layered water column of this meromictic ice-covered lake. The unprecedented *Woesearchaeota* diversity detected in Lake A might therefore be explained by substantial metabolic and catabolic flexibility within the *Woesearchaeota*, suggesting a highly dynamic evolution of gene content in the phyla that would allow niche partitioning and alleviation of interspecies competition. The large metabolic repertoire within the *Woesearchaeota* could be predicted given the widespread distribution of this radiation in diverse aquatic systems and the numerous subgroups identified by 16 S rRNA gene analysis [52], but this high taxonomic and functional diversity within a single ecosystem is rare. Nonetheless, similar findings have been reported in oligotrophic stratified lakes [13], marine systems [53] as well as in groundwater [55], suggesting an adaptation of DPANN archaea to constrained and oligotrophic systems.

The metabolic potential of the MAGs was not consistent with their phylogeny as inferred from ribosomal protein genes (Fig. 2); for example, sulfide reduction and the AMP salvage pathway were found in several phylogenetically distanced lineages. This discrepancy as well as the limited number of genes detected in the genetic core of *Woesearchaeota* imply horizontal gene transfer as the most likely mechanism leading to the metabolic diversification of *Woesearchaeota*, as suggested for other DPANN archaea [54].

### Genomic evidence for predatory lifestyles in *Woesearchaeota*

Our analysis of CAZy genes revealed the potential to degrade peptidoglycan through large lytic murein transglycosylase in five phylogenetically distant MAGs, including the two most abundant *Woesearchaeota* MAGs recovered from Lake A. Lytic murein transglycosylase (CAZy ID GH23; Fig. 5) cleaves glycosidic linkages between N-actetylmuramoyl and N-acetylglucosaminyl residues, degrading murein (peptidoglycan) strands, the main components of bacterial cell walls. An analog of this gene was also reported in another *Woesearchaeota* genome (AR20) from a deep terrestrial aquifer [11] and detected in four other publicly available Woesarchaeotal MAGs (Fig. 5). This result suggests that some *Woesearchaeota* have the potential to attack and degrade bacterial peptidoglycan.

Interestingly, three of our putative peptidoglycan-degrading MAGs also include genes for a putative pore-forming toxin (*tlyC*) and numerous transport systems, conferring the ability to lyse bacterial cells in addition to the degradation of peptidoglycan. Analogs of hemolysin and membrane-bound lytic murein transglycosylase genes have been recently identified in the genome of *Vampirococcus lugosii*, a confirmed predatory bacterium of the Candidate Phyla Radiation [56] that shares numerous genomic characteristics with DPANN archaea [10]. Furthermore, phylogenetic analysis of the sequences revealed homologs of this gene in other DPANN archaea genomes, including *Woesarchaeota*, *Aenigmarchaeota,* and *Nanoarchaeota*, supporting the potential of membrane-damaging toxin production in DPANN archaea (Fig. 6), The presence of these genes would therefore be consistent with the pathogenicity as proposed for some *Woesearchaeota* [11]. Coverage of their MAGs indicted that potential predatory populations were the most abundant *Woesearchaeota*, and most notably in the 34 and 40 m samples (Fig. 1, group A). This pathogenicity may confer a selective advantage at this depth, where multiple potential prey co-occur and decaying microorganisms sink from within and above the oxycline. Like the CPR bacterium *Vampirococcus lugosii*, putative predatory *Woesearchaeota* could play a virus-like ecological role in ecosystems, controlling bacterial populations by a novel form of predation [56].

To identify potential targets of the putative predatory *Woesearchaeota*, a correlation matrix including bacterial MAGs recovered from the same samples [26] was calculated. Few low abundance *Parcubacteria*, and *Deltaproteobacteria* MAGs and highly abundant *Marinimicrobia* and *Omnitrophica* MAGs were identified as strongly correlated with the putative predatory *Woesearchaeota* (*R* > 0.99; *p* < 0.0001, Fig. 3). Although these correlations might reveal syntrophic relationships, involving acetate, lactate or hydrogen exchanges, as previously proposed [52], they would also be consistent with predation. All of the bacterial MAGs contain peptidoglycan biosynthesis genes (*murABCDEFGHJQU*), consistent with peptidoglycan in their cell walls, and thus potential vulnerability to attack by predatory *Woesearchaeota*.

### Metabolic cooperation between Woesearchaeota lineages

Due to their small cell sizes, limited genome and lack of essential enzymatic machinery, many DPANN organisms appear to fundamentally depend on other organisms for basic resources [10]. However, the extent to which they rely on other organisms is predicted to vary widely, depending on the inventory of metabolic capacities found in each group. A previous co-occurrence network calculated with *Woesearchaeota* 16 S rRNA genes suggested a syntrophic relationship with methanogenic Archaea [52]. However, no methanogens were detected in our metagenomic dataset, therefore this co-occurrence might simply result from confounding niche preferences (i.e., anoxic and sulfidic environments). Interestingly, correlation analysis indicated that potential predatory *Woesearchaeota* strongly co-vary (*R*^2^ > 0.99; *p* < 0.0001) with other *Woesearchaeota* lineages of different genetic capabilities (Fig. 3). Bin 69, identified as potential pathogen and peptidoglycan degrader, was strongly correlated with Bins 482 and 312, with the nucleotide salvage pathway and Bin 595 with sulfur and acetate-based metabolism. Bins 683, 185, and 300, all suspected to be pathogens from this analysis, were strongly correlated together in a cluster including five other *Woesearchaeota* MAGs with potential for amino acid degradation (Bin 350), nucleotide salvage pathway (Bins 598 and 129) and fermentation (Bins 210 and 305). Although these correlations might indicate similar ecological niches despite different putative physiology, as is possible with methanogens [52], these results would also be consistent with the formation of intra-phyla consortia of *Woesearchaeota* species with metabolic complementarities. The non-pathogenic *Woesearchaeota* may benefit from released metabolites of the killed bacteria by specializing in substrates not used by the pathogens, thereby avoiding direct competition with other *Woesarchaeota* species. Such co-operative interactions among DPANN would be consistent with the Black Queen hypothesis [57], favored by the physically stable Lake A ecosystem [58, 59]. High-resolution microscopy with specific probes could be used to test whether this cooperative metabolism was based on physical proximity, which would provide competition-free ecological niches for DPANN archaea in highly competitive, resource-limited environments.

## Conclusions

Our observations from an extreme ice-capped, permanently stratified lake with pronounced redox gradients provide new insights into the genetic potential of DPANN archaea, expanding their known potential ecophysiology over a wide range of oxygen and salinity conditions. Substantial metabolic flexibility was identified, potentially driven by horizontal gene transfer, including aerobic and anaerobic metabolism based on transformations of sulfur compounds. A putative vampiristic metabolism was also identified in the predominant *Woesarchaeota* populations. These populations, in collaboration with other cellular compound-degrading DPANN populations, may potentially recycle part of the microbial biomass through a DPANN archaeal shunt within the microbial loop.

## Supplementary information

Supplementary Table 1

Supplementary Figure 1

Supplementary Material 1

Supplementary Material 2

## Data Availability

Assembled metagenome data are available in IMG/MR (https://img.jgi.doe.gov/mer/) under the following accession numbers: 3300033443, 3300033444, 3300033445, 3300033439, 3300033411, 3300033473, 3300033474, 3300033495 Co-assembly is also available on IMG/MR under accession number 3300033064. Raw amplicon sequences and bin files were deposited in the NCBI public database under Bioproject PRJNA616293. The concatenated ribosomal gene sequences alignment used for the phylogenomic tree and the phylogenetic tree generated by GTDB_tk are available in Supplementary Material 1 and 2 respectively. In-house scripts used in this study are available on GitHub/CruaudPe. Environmental metadata were previously published [25, 26] and additional data are available in the Nordicana D database (http://www.cen.ulaval.ca/nordicanad).
