# Supplementary material for: Genomic evidence of functional diversity in DPANN archaea, from oxic species to anoxic vampiristic consortia
Source: ISME Commun. 2022 Jan 20;2:4. doi: 10.1038/s43705-022-00088-6 (PMC9723730; doi:10.1038/s43705-022-00088-6)

Supplementary Figure 1. Archaeal community composition of the Lake A samples. Relative proportion of archaeal lineages identified in Lake A using the metagenomic dataset (M), or by DNA-based (D) and RNA-based (R) 16S rRNA gene sequencing.

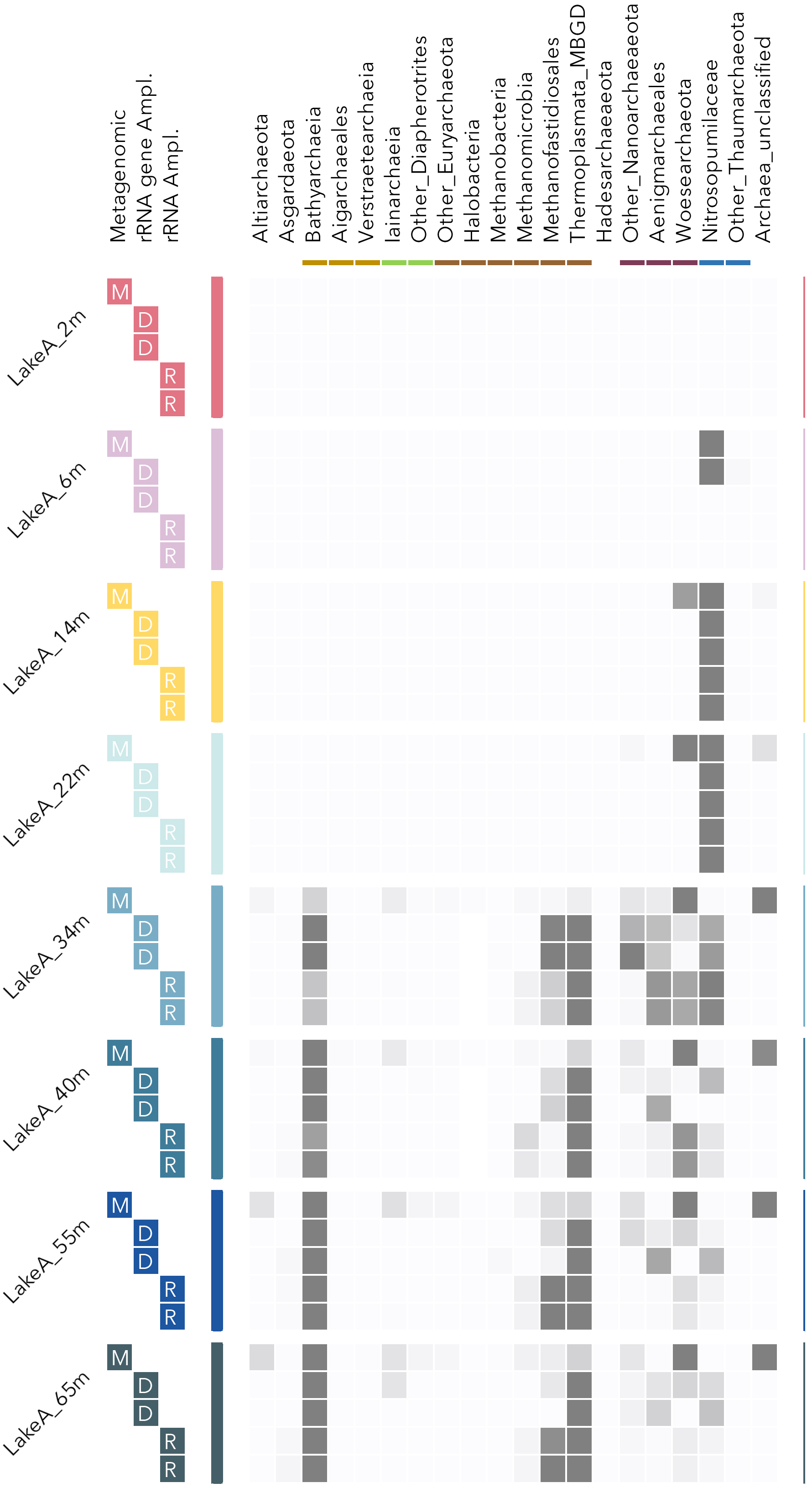

Supplement: Supplementary file 2 — Supplementary Figure 1 [file 43705_2022_88_MOESM2_ESM.pdf]
