# Supplementary material for: Genomic evidence of functional diversity in DPANN archaea, from oxic species to anoxic vampiristic consortia
Source: ISME Commun. 2022 Jan 20;2:4. doi: 10.1038/s43705-022-00088-6 (PMC9723730; doi:10.1038/s43705-022-00088-6)
Supplement: Supplementary file 4 — Supplementary Material 2 [file 43705_2022_88_MOESM4_ESM.pdf]

Supplementary Material 2. Archaeal phylogenetic tree constructed using GTDB-Tk using genomes with at least 40% of the 122 archaeal marker genes.

((GB_GCA_003663295.1:0.236401,GB_GCA_003663355.1:0.140537)'1.0:p__QMZS01;c__QMZS01;o_
_QMZS01;f__QMZS01;g__QMZS01':0.502437,(((((((((((((((((((((((((((((((((((((((((((((((((((((RS_GCF_002286985
.1:0.0151983,RS_GCF_004114995.1:0.0169901)0.969:0.00421523,RS_GCF_000337915.1:0.0145305)
0.599:0.00339736,RS_GCF_007671685.1:0.0152311)0.0:0.0029097,RS_GCF_002727125.1:0.0192273
)0.855:0.00288531,(RS_GCF_000739595.1:0.0143872,RS_GCF_003781945.1:0.0185155)0.998:0.0048
546)0.988:0.00338389,RS_GCF_000022205.1:0.0247001)0.738:0.00465987,RS_GCF_000337375.1:0.
0134867)0.0:0.00470121,RS_GCF_000337355.1:0.0166951)0.996:0.00797608,RS_GCF_003990725.2:
0.0300801)1.0:0.010925,(((RS_GCF_002252755.1:0.0180211,RS_GCF_007671725.1:0.0153736)0.542
:0.00429996,RS_GCF_003721435.2:0.0193905)0.711:0.00444494,RS_GCF_001261915.1:0.0239715)1
.0:0.0135196)0.682:0.00676663,(RS_GCF_000336995.1:0.0234435,GB_GCA_001564205.1:0.0368203
)1.0:0.0167166)0.971:0.00825557,RS_GCF_000746205.1:0.0477031)0.823:0.00704668,((((((RS_GCF_
000337035.1:0.0181577,RS_GCF_002114285.1:0.018666)0.998:0.00709848,RS_GCF_001280455.1:0.
0195882)0.867:0.00288306,(RS_GCF_000336875.1:0.0226948,RS_GCF_005435165.1:0.025429)1.0:0.
0104405)0.962:0.00370767,(((RS_GCF_000296615.1:0.00823346,RS_GCF_003666015.1:0.0149645)0.
979:0.00496337,RS_GCF_002355655.1:0.0141082)0.335:0.00289395,RS_GCF_000337335.1:0.01241
14)0.633:0.00393168)0.353:0.00290905,RS_GCF_900102375.1:0.0159906)0.988:0.00658507,RS_GCF
_900111935.1:0.0233625)0.024:0.00349623,(((RS_GCF_000337075.1:0.00794994,RS_GCF_00033741
5.1:0.00935967)0.996:0.00502747,GB_GCA_003023405.1:0.0239172)0.999:0.00853685,RS_GCF_004
114375.1:0.0376571)0.982:0.00560228)1.0:0.0383691)0.802:0.00854172,RS_GCF_009742825.1:0.05
07371)1.0:0.0208944,((((((RS_GCF_001542905.1:0.00772223,RS_GCF_002252985.1:0.019476)1.0:0.0
120739,RS_GCF_900182635.1:0.0136548)0.0:0.0053661,RS_GCF_900113615.1:0.0150282)0.999:0.0
0959184,RS_GCF_003697845.1:0.0184195)0.032:0.00598049,RS_GCF_003287355.1:0.024637)1.0:0.
0163528,GB_GCA_000496175.1:0.0643188)1.0:0.0200728)0.995:0.013453,((GB_GCA_003554605.1:
0.0247183,RS_GCF_900188065.1:0.0270119)1.0:0.0551738,RS_GCF_009806985.1:0.0528055)0.876:
0.0152493)'1.0:g__Halorubrum':0.0631034,(((RS_GCF_002355635.1:0.0123678,RS_GCF_900107205.
1:0.0111444)1.0:0.016663,RS_GCF_900108505.1:0.0289458)'1.0:g__Halopenitus':0.074437,RS_GCF_
001462205.1:0.0767548)0.078:0.0172171)1.0:0.0328994,GB_GCA_000496235.1:0.220605)0.524:0.0
201037,RS_GCF_002952775.1:0.136171)1.0:0.0242292,((((((RS_GCF_003605575.1:0.0148112,RS_GC
F_003605635.1:0.0325787)1.0:0.0137921,RS_GCF_006861655.1:0.0194657)1.0:0.0367321,RS_GCF_
006861665.1:0.0378947)0.953:0.015527,GB_GCA_000496215.1:0.0568653)'1.0:g__Halonotius':0.05
36844,(((GB_GCA_001563795.1:0.0474485,GB_GCA_003554115.1:0.0395008)1.0:0.0359163,RS_GCF
_900109065.1:0.0569752)'1.0:g__Halohasta':0.028274,GB_GCA_001563965.1:0.0983567)0.784:0.02
03116)1.0:0.0593333,((RS_GCF_005239175.1:0.00834592,RS_GCF_005543295.1:0.0135823)1.0:0.06
02661,RS_GCF_013342525.1:0.0877142)'1.0:g__N1521':0.0575085)1.0:0.047666)0.987:0.0225086,((
(((RS_GCF_001307315.1:0.0199337,RS_GCF_900115675.1:0.0266104)0.381:0.00823468,RS_GCF_00
0739555.1:0.019472)1.0:0.0148911,(RS_GCF_001282785.1:0.0276189,RS_GCF_002025255.2:0.0258
492)0.999:0.0153435)1.0:0.0403181,RS_GCF_000224475.1:0.0977032)'1.0:g__Halolamina':0.112486
,(((RS_GCF_013401515.1:0.043174,RS_GCF_013402875.1:0.0582341)1.0:0.0397877,RS_GCF_000495
475.1:0.0940727)0.341:0.0174288,((RS_GCF_009831625.1:0.0287137,RS_GCF_900129775.1:0.03735
34)0.88:0.0156236,GB_GCA_003020945.1:0.0627484)1.0:0.0688457)'1.0:g__Halobaculum':0.063725
8)1.0:0.0320507)0.944:0.01869,((((((((RS_GCF_000739575.1:0.029855,RS_GCF_007655485.1:0.0392
078)1.0:0.0184862,RS_GCF_004799685.1:0.0305486)1.0:0.0185319,((RS_GCF_014212355.1:0.03275
92,GB_GCA_014646875.1:0.0423412)1.0:0.0632647,RS_GCF_900107195.1:0.053676)0.932:0.013985
)0.091:0.00566159,(RS_GCF_003665925.1:0.0236931,RS_GCF_003665935.1:0.0273979)1.0:0.024015

7)'1.0:g__Halobellus':0.0280929,(((RS_GCF_000237865.1:0.029305,GB_GCA_000415965.1:0.027214
2)0.974:0.0152584,GB_GCA_000415985.1:0.0414075)1.0:0.0874373,GB_GCA_000416005.1:0.18987
2)'1.0:g__Haloquadratum':0.0781858)1.0:0.0403342,((((RS_GCF_000172995.2:0.0352793,RS_GCF_9
00112175.1:0.0301297)0.996:0.0153945,RS_GCF_000337095.1:0.0500559)0.558:0.0109523,RS_GCF
_900115785.1:0.0462417)'1.0:g__Halogeometricum':0.0367201,(RS_GCF_900100875.1:0.0236341,R
S_GCF_900113245.1:0.0380614)'1.0:g__Halopelagius':0.0491901)0.987:0.0197397)1.0:0.0292572,(((
(((((RS_GCF_000337795.1:0.00371579,RS_GCF_000337835.1:0.00267702)0.99:0.00361983,RS_GCF_
001368915.1:0.00817474)0.778:0.0033331,RS_GCF_000025685.1:0.0117763)0.96:0.00452092,RS_G
CF_001469875.2:0.00420693)0.589:0.0028303,((RS_GCF_000336775.1:0.00348563,RS_GCF_000336
815.1:0.00590086)0.996:0.00347496,RS_GCF_001482285.1:0.00411501)0.894:0.00283026)1.0:0.028
6701,(RS_GCF_000306765.2:0.0247717,RS_GCF_000337815.1:0.0251916)1.0:0.0145888)1.0:0.01366
08,((RS_GCF_001469865.1:0.00847919,RS_GCF_009674605.1:0.00556349)0.998:0.00984308,(RS_GC
F_009674585.1:0.0195819,RS_GCF_009674625.1:0.0225298)0.969:0.00794319)1.0:0.0206229)0.993
:0.0138932,(RS_GCF_000336755.1:0.00606194,RS_GCF_000336955.1:0.00570274)1.0:0.0330193)'1.
0:g__Haloferax':0.0919019)0.998:0.0230166,((RS_GCF_002906575.1:0.0334296,RS_GCF_007004735.
1:0.031151)1.0:0.0255722,RS_GCF_009176545.1:0.0493236)'1.0:g__Salinigranum':0.0956929)0.999:
0.01966,((((RS_GCF_900110465.1:0.0324896,RS_GCF_900114455.1:0.031228)1.0:0.0230676,RS_GCF
_900103715.1:0.0388411)'1.0:g__Halogranum':0.0630498,(RS_GCF_002844195.1:0.0457511,RS_GCF
_004116405.1:0.05713)'1.0:g__Halegenticoccus':0.0535718)0.967:0.0201959,(RS_GCF_001469955.1:
0.0252065,RS_GCF_003439925.1:0.0193595)'1.0:g__Haloprofundus':0.0916516)1.0:0.0237869)1.0:0.
0293221)0.53:0.0142015,((((((RS_GCF_003336245.1:0.0387139,RS_GCF_003342675.1:0.0152371)1.0
:0.0152162,RS_GCF_000427685.1:0.0349292)1.0:0.016877,RS_GCF_003856835.1:0.0413947)0.661:0
.00991914,RS_GCF_900107665.1:0.0536367)0.418:0.0188454,RS_GCF_009762275.1:0.0479866)'1.0:
g__Haloplanus':0.0819997,GB_GCA_000496195.1:0.238229)0.659:0.0377511)'1.0:f__Haloferacaceae
':0.069339,(((((((((((((((((((((((RS_GCF_000337615.1:0.0120892,RS_GCF_000731985.1:0.00597296)0.9
95:0.00575659,RS_GCF_000337175.1:0.00877404)1.0:0.026412,(RS_GCF_000337195.1:0.0160152,R
S_GCF_005576615.1:0.0110255)1.0:0.017271)0.923:0.00786416,RS_GCF_002494345.1:0.030609)0.0
:0.00549709,RS_GCF_900110865.1:0.036869)0.873:0.00617895,(RS_GCF_002572525.1:0.017157,RS
_GCF_013456555.1:0.0244107)1.0:0.023655)0.999:0.00925689,((RS_GCF_000230735.2:0.00502858,
RS_GCF_000337115.1:0.00467936)1.0:0.00733258,(GB_GCA_000690595.2:0.0116046,RS_GCF_0019
53745.1:0.0118653)0.745:0.00448883)1.0:0.0283493)0.718:0.00759582,(((RS_GCF_000337475.1:0.0
0923348,RS_GCF_900111485.1:0.0120251)0.262:0.0036261,RS_GCF_004217335.1:0.00697202)1.0:0
.0100493,RS_GCF_005938085.1:0.017619)1.0:0.0284977)0.99:0.00928843,RS_GCF_013402815.1:0.0
554873)0.866:0.00916707,RS_GCF_900110455.1:0.0470818)'1.0:g__Natrinema':0.0173335,(((((((RS_
GCF_004799645.1:0.0228708,RS_GCF_900156475.1:0.026232)0.925:0.0110532,RS_GCF_000337735.
1:0.0169349)0.284:0.00784626,RS_GCF_009392895.1:0.0224075)1.0:0.0303954,RS_GCF_003670115
.1:0.0518996)0.962:0.0125011,((RS_GCF_000383975.1:0.0130614,RS_GCF_900100335.1:0.0136456)
1.0:0.0286854,(RS_GCF_001971705.1:0.00789227,RS_GCF_900108095.1:0.00603214)1.0:0.0572626)
0.996:0.0123939)0.0:0.00473829,RS_GCF_009834785.1:0.0463367)'0.999:g__Natronorubrum':0.016
7474,((((RS_GCF_000337495.1:0.00577732,RS_GCF_003977755.1:0.0066818)0.323:0.00290421,RS_
GCF_013342135.1:0.00925521)0.996:0.00846021,RS_GCF_000025325.1:0.0113774)'1.0:g__Haloterri
gena':0.0394273,RS_GCF_000337215.1:0.0518943)0.334:0.0134114)0.998:0.0132856)0.976:0.00824
834,(((((RS_GCF_000226975.2:0.0106758,RS_GCF_900112205.1:0.00664787)'1.0:g__Halobiforma':0.
0368945,RS_GCF_000337895.1:0.037725)0.997:0.0117901,(RS_GCF_000230715.2:0.0535806,RS_GC
F_900104065.1:0.0292353)1.0:0.0184636)1.0:0.0215674,RS_GCF_000455365.1:0.050321)0.462:0.00
984008,((RS_GCF_000217715.1:0.0124144,RS_GCF_003610195.1:0.0127515)'1.0:g__Halopiger':0.02
61134,(RS_GCF_002177135.1:0.0238171,RS_GCF_011043775.1:0.0202917)'1.0:g__Natronolimnobiu
s':0.0502634)0.993:0.0119222)0.908:0.00609903)0.911:0.00662594,((((RS_GCF_000337575.1:0.0068

3175,RS_GCF_001861355.1:0.00850293)0.966:0.00378128,RS_GCF_000025625.1:0.0117571)0.677:0
.00464092,RS_GCF_000337135.1:0.0135166)1.0:0.0332836,((RS_GCF_000337535.1:0.00620374,RS_
GCF_000337595.1:0.0102219)0.549:0.00425893,RS_GCF_000337555.1:0.00992936)1.0:0.0421896)'1
.0:g__Natrialba':0.0336308)0.592:0.00819745,(RS_GCF_000455345.1:0.0571063,RS_GCF_006543045
.1:0.0658524)0.997:0.0138726)0.0:0.00675916,(((RS_GCF_000328685.1:0.0298875,RS_GCF_000337
695.1:0.0303054)0.2:0.00842968,(RS_GCF_000337675.1:0.0160146,GB_GCA_003559215.1:0.016377
)1.0:0.0138299)1.0:0.0294295,RS_GCF_008122205.1:0.0457569)'1.0:g__Natronococcus':0.030578)0.
99:0.0105445,((RS_GCF_003841465.1:0.0537897,RS_GCF_003841485.1:0.057123)0.784:0.0100285,(
RS_GCF_003841505.1:0.040807,RS_GCF_008245225.1:0.0506193)0.996:0.0149366)'1.0:g__Natrarch
aeobius':0.0177944)0.574:0.011303,(((GB_GCA_001563805.1:0.0219572,RS_GCF_003430825.1:0.02
30918)1.0:0.0312175,RS_GCF_002156705.1:0.0509798)0.955:0.0131588,GB_GCA_001563885.1:0.06
39658)'0.999:g__Natrarchaeobaculum':0.0145544)1.0:0.0241958,(((RS_GCF_000691505.1:0.008334
1,RS_GCF_900116205.1:0.0103601)1.0:0.0143927,RS_GCF_000517625.1:0.0197746)'1.0:g__Halosta
gnicola':0.0696377,((GB_GCA_001564285.1:0.00110037,GB_GCA_003552325.1:0.00166548)1.0:0.06
0113,GB_GCA_001564275.1:0.0560359)'1.0:g__Tc-
Br11':0.0744489)0.373:0.0194644)0.963:0.0183881,(((RS_GCF_000328525.1:0.00830782,RS_GCF_00
0337515.1:0.0118905)1.0:0.072139,GB_GCA_007135505.1:0.0677144)1.0:0.0343906,GB_GCA_0071
19345.1:0.0793338)'1.0:g__Halovivax':0.0513232)0.294:0.0106102,((GB_GCA_001564115.1:0.02808
92,GB_GCA_003552885.1:0.0403182)'1.0:g__Tc-Br11-
E2g8':0.0864549,GB_GCA_001564255.1:0.0813246)0.938:0.020788)0.765:0.0169601,RS_GCF_00334
4565.1:0.124149)1.0:0.0733141,(RS_GCF_011440375.1:0.13785,RS_GCF_900103505.1:0.103886)1.0:
0.0583826)'0.995:f__Natrialbaceae':0.0209178,(((((GB_GCA_003023565.1:0.0445856,RS_GCF_00367
5875.1:0.0180898)1.0:0.0272938,RS_GCF_005954745.1:0.0576589)0.924:0.0140696,(RS_GCF_00367
5855.1:0.0253256,RS_GCF_004785955.1:0.0237818)1.0:0.0340731)'1.0:f__QS-9-68-
17;g__Halostella':0.0600185,RS_GCF_900215575.1:0.119987)1.0:0.0285686,((GB_GCA_003022865.1
:0.191372,GB_GCA_003023195.1:0.130191)'1.0:f__SW-7-71-33;g__SW-7-71-
33':0.0767867,RS_GCF_000403645.1:0.184923)0.997:0.0382392)0.626:0.0188546)0.96:0.020137)0.2
31:0.0126592,((((((((RS_GCF_003369835.1:0.0502813,RS_GCF_010747475.1:0.0423893)0.361:0.013
298,RS_GCF_005239435.1:0.0359223)0.949:0.00947041,(RS_GCF_004087835.1:0.03795,RS_GCF_00
4765815.2:0.041141)1.0:0.0206503)0.812:0.00976049,RS_GCF_004765785.1:0.0446243)1.0:0.02283
,((RS_GCF_003382685.1:0.0506666,RS_GCF_004765805.1:0.070997)1.0:0.0413979,GB_GCA_003023
465.1:0.0556264)0.981:0.0134775)1.0:0.0175804,RS_GCF_004765795.1:0.065612)0.554:0.0136421,
RS_GCF_004143485.2:0.0688324)0.887:0.0174995,RS_GCF_008831545.1:0.0815677)'1.0:g__Halorus
sus':0.040353,(((RS_GCF_000376445.1:0.0365736,RS_GCF_001625445.1:0.0340356)1.0:0.0351197,(
RS_GCF_000710615.1:0.0148437,RS_GCF_900156425.1:0.00794619)1.0:0.0521387)1.0:0.0566735,R
S_GCF_003298465.1:0.0842819)'1.0:g__Haladaptatus':0.035857)'1.0:f__Haladaptataceae':0.0643142
)0.988:0.0177554,(((((((((((((((RS_GCF_000336895.1:0.00520632,RS_GCF_012726115.1:0.00532613)
1.0:0.004872,RS_GCF_000336635.1:0.0122842)0.82:0.00152941,(RS_GCF_000011085.1:0.00936776,
RS_GCF_001485575.1:0.00763731)0.993:0.00373185)0.993:0.00416684,(RS_GCF_000223905.1:0.00
466889,RS_GCF_000336615.1:0.00723507)0.998:0.00534418)0.794:0.0022858,RS_GCF_008729015.
1:0.0127422)0.925:0.0024898,((RS_GCF_001280425.1:0.00793828,RS_GCF_002844335.1:0.0097110
6)1.0:0.00631017,RS_GCF_000337775.1:0.0162766)0.0:0.00207235)0.998:0.00731943,RS_GCF_0054
06125.1:0.0132562)1.0:0.0265708,RS_GCF_010119195.1:0.0358247)'1.0:g__Haloarcula':0.0292224,(
((RS_GCF_004681185.1:0.0140246,RS_GCF_010119205.1:0.0183956)1.0:0.0428691,GB_GCA_01464
7235.1:0.040767)0.523:0.0119754,(GB_GCA_003020925.1:0.0459668,GB_GCA_003021755.1:0.0425
255)1.0:0.04881)'1.0:g__Halomicroarcula':0.0224343)1.0:0.0542245,((((RS_GCF_000023965.1:0.003
18815,RS_GCF_000379085.1:0.00599233)0.999:0.00786636,RS_GCF_009617995.1:0.00571193)1.0:0
.0494052,GB_GCA_003021685.1:0.0776956)1.0:0.0474355,RS_GCF_900114435.1:0.107347)'0.905:g

__Halomicrobium':0.0275402)1.0:0.0413381,(((((RS_GCF_013415885.1:0.0205335,RS_GCF_0134159
05.1:0.0129251)0.122:0.00575934,RS_GCF_000337455.1:0.0187363)1.0:0.0188152,RS_GCF_004698
125.1:0.0229629)0.997:0.0187171,(GB_GCA_003023165.1:0.0631699,RS_GCF_003730195.1:0.04662
36)0.959:0.0148971)'1.0:g__Halosimplex':0.0507273,((GB_GCA_003021305.1:0.0818896,GB_GCA_0
03021655.1:0.0859808)0.867:0.0196186,GB_GCA_003023785.1:0.0966076)0.999:0.0252327)1.0:0.0
591436)0.219:0.017484,(((((RS_GCF_004944975.1:0.0388976,RS_GCF_900880625.1:0.0391593)1.0:0
.026718,(RS_GCF_009690625.1:0.030466,RS_GCF_013839425.1:0.0267496)1.0:0.0565017)0.903:0.0
211509,(RS_GCF_000023945.1:0.0278393,RS_GCF_000470655.1:0.0405135)1.0:0.0431601)'1.0:g__H
alorhabdus':0.0659474,RS_GCF_003058365.1:0.224848)0.997:0.0317658,(RS_GCF_004799665.1:0.0
815679,RS_GCF_009741825.1:0.0966793)'1.0:g__Halapricum':0.0641835)0.742:0.0322503)0.626:0.0
192718,(((((((GB_GCA_003021015.1:0.00825196,GB_GCA_003022845.1:0.0183084)1.0:0.0306117,G
B_GCA_003022725.1:0.0370629)0.922:0.0217662,GB_GCA_003022085.1:0.0551712)1.0:0.058724,G
B_GCA_003021335.1:0.225394)0.891:0.0187618,((GB_GCA_003551265.1:0.116092,RS_GCF_900100
385.1:0.100846)1.0:0.0334888,GB_GCA_003021285.1:0.110658)1.0:0.0329492)0.415:0.0128794,(RS
_GCF_004015825.1:0.0829291,RS_GCF_009831575.1:0.085705)1.0:0.0522424)1.0:0.0183984,((GB_G
CA_001564135.1:0.0312329,GB_GCA_003551945.1:0.0408838)1.0:0.110263,GB_GCA_000416085.1:
0.181064)1.0:0.0310466)'1.0:g__Halovenus':0.0397808,(((GB_GCA_003022745.1:0.0700449,GB_GCA
_003023485.1:0.0624291)1.0:0.0709145,RS_GCF_009831455.1:0.113164)0.91:0.026306,RS_GCF_00
9789175.1:0.110174)0.996:0.0191948)1.0:0.036998)1.0:0.0233939,((((RS_GCF_004118325.1:0.0263
723,RS_GCF_900102305.1:0.0198761)0.247:0.00628376,RS_GCF_900110215.1:0.0178548)0.996:0.0
106887,RS_GCF_001989615.1:0.0256011)1.0:0.0582298,RS_GCF_005049285.1:0.0834899)'1.0:g__H
alorientalis':0.066148)0.907:0.017959,GB_GCA_003021105.1:0.142761)1.0:0.0190576,(((((((((GB_G
CA_003022945.1:0.0177951,GB_GCA_003023535.1:0.0192472)0.64:0.00463854,GB_GCA_00302096
5.1:0.0101941)1.0:0.0542776,(GB_GCA_003022815.1:0.0128007,GB_GCA_003022905.1:0.0096859)1
.0:0.0679936)0.905:0.0191754,((GB_GCA_003021005.1:0.0266572,GB_GCA_003021745.1:0.0140574
)1.0:0.0872072,GB_GCA_003021325.1:0.0481903)0.136:0.0117808)1.0:0.0549906,((((RS_GCF_00059
1055.1:0.0571618,RS_GCF_013839515.1:0.0450724)1.0:0.0269143,RS_GCF_005239135.1:0.0636308
)0.908:0.0169871,GB_GCA_007121575.1:0.0966452)1.0:0.0236006,RS_GCF_000026045.1:0.0880259
)1.0:0.0250713)0.385:0.00820041,((RS_GCF_009741925.1:0.00549393,RS_GCF_013391635.1:0.0075
5028)1.0:0.0606812,(RS_GCF_013391085.1:0.0856476,RS_GCF_013391105.1:0.0684726)0.0:0.01511
5)0.997:0.015858)0.993:0.0135126,GB_GCA_007135435.1:0.10497)0.641:0.0189532,GB_GCA_00712
2455.1:0.115276)'1.0:g__Natronomonas':0.0555331,((((RS_GCF_001950595.1:0.0124253,RS_GCF_00
9184545.1:0.0194242)1.0:0.0230707,GB_GCA_003021785.1:0.0680484)1.0:0.0875467,(GB_GCA_003
021175.1:0.0156704,GB_GCA_003023185.1:0.0182495)1.0:0.073015)'1.0:g__CBA1134':0.0858704,((
GB_GCA_003020985.1:0.0534155,RS_GCF_007421925.1:0.0675259)'1.0:g__QS-4-70-
19':0.0850517,GB_GCA_003022005.1:0.140837)0.94:0.0310691)0.99:0.0245989)1.0:0.0309066,((GB
_GCA_003021975.1:0.0960666,RS_GCF_009791395.1:0.126927)1.0:0.0404336,RS_GCF_003862495.1
:0.185822)'1.0:g__Halomarina':0.0474275)0.995:0.0241801)'0.998:f__Haloarculaceae':0.0225983,((((
((RS_GCF_000336695.1:0.016162,RS_GCF_000336715.1:0.0138991)1.0:0.0308894,RS_GCF_0007552
45.1:0.0409758)1.0:0.0494683,(RS_GCF_000336675.1:0.0165216,RS_GCF_009900715.1:0.0177048)1
.0:0.0931924)1.0:0.0292042,((RS_GCF_000334895.1:0.0166995,GB_GCA_003022685.1:0.0446015)0.
523:0.0100366,(RS_GCF_000336915.1:0.0244747,RS_GCF_000336935.1:0.0260934)0.968:0.0106305
)1.0:0.0664785)1.0:0.0495939,GB_GCA_003021045.1:0.143008)1.0:0.0389294,((GB_GCA_00302123
5.1:0.0659186,GB_GCA_003022925.1:0.0876602)0.694:0.0195886,(GB_GCA_003021705.1:0.040199
6,GB_GCA_003021725.1:0.0364352)1.0:0.0536682)1.0:0.128865)'1.0:f__Halococcaceae;g__Halococc
us':0.0582152)0.987:0.0199692)0.445:0.0146421,((((((((RS_GCF_001485535.1:0.0263203,RS_GCF_0
01488575.1:0.0159619)0.869:0.00636094,RS_GCF_009741975.1:0.029513)0.985:0.010658,RS_GCF_
011734195.1:0.0304696)1.0:0.0253798,RS_GCF_009831555.1:0.0597325)1.0:0.0278176,RS_GCF_00

0230955.2:0.0717055)0.878:0.017111,(RS_GCF_008124605.1:0.0984879,RS_GCF_900110535.1:0.03
57435)1.0:0.0597556)'1.0:g__Halobacterium':0.0709228,((((GB_GCA_014647115.1:0.0144043,GB_G
CA_014647155.1:0.0328585)0.998:0.0148485,RS_GCF_000474235.1:0.0425558)1.0:0.0345124,GB_G
CA_014647455.1:0.0463909)'1.0:g__Halarchaeum':0.0829345,(RS_GCF_007833275.1:0.119652,GB_
GCA_014647475.1:0.134683)0.986:0.0311992)1.0:0.027607)1.0:0.0351795,(RS_GCF_001011115.1:0.
138929,RS_GCF_001886955.1:0.179794)1.0:0.0571304)'1.0:f__Halobacteriaceae':0.0495244,(GB_GC
A_003021085.1:0.155167,GB_GCA_007122875.1:0.212931)0.991:0.0359959)0.804:0.0172981)0.986:
0.0284181,(((RS_GCF_000196895.1:0.0400882,RS_GCF_003697815.1:0.0506693)0.509:0.0151786,RS
_GCF_001593955.1:0.0315431)'1.0:g__Halalkalicoccus':0.10453,(GB_GCA_007128515.1:0.0332508,G
B_GCA_007129135.1:0.0299148)'1.0:g__SKXI01':0.124227)'1.0:f__Halalkalicoccaceae':0.0492637)1.0
:0.044564,((GB_GCA_007118075.1:0.176734,GB_GCA_007118145.1:0.166661)'1.0:f__SKSH01;g__SK
SH01':0.164292,(GB_GCA_007118975.1:0.190438,GB_GCA_007123105.1:0.179242)'1.0:f__SKNY01;g
__SKNY01':0.0940959)0.0:0.032517)1.0:0.113006,((GB_GCA_002503845.1:0.0509931,GB_GCA_0098
89685.1:0.068509)1.0:0.0373817,GB_GCA_009889625.1:0.0854399)'1.0:f__UBA12382;g__UBA1238
2':0.309842)'1.0:c__Halobacteria;o__Halobacteriales':0.322155,(((((((((((((GB_GCA_002839605.1:0.
0188047,GB_GCA_013331015.1:0.0243721)1.0:0.0306115,GB_GCA_002508495.1:0.0727047)0.996:0
.0187649,GB_GCA_012798365.1:0.0585163)1.0:0.026893,(RS_GCF_000327485.1:0.0205718,GB_GCA
_002835825.1:0.0300133)1.0:0.0729069)0.998:0.0209046,(((GB_GCA_002502245.1:0.0417964,GB_G
CA_003157895.1:0.0413776)0.817:0.0107896,GB_GCA_002497485.1:0.0561298)0.908:0.013474,GB
_GCA_002067035.1:0.0667701)1.0:0.0283467)1.0:0.0274372,(GB_GCA_002839675.1:0.0303724,GB_
GCA_903832665.1:0.0411865)1.0:0.0701117)0.473:0.0149794,(((GB_GCA_002495065.1:0.0612975,
GB_GCA_003154335.1:0.0785929)0.425:0.0132463,GB_GCA_002495055.1:0.0595092)0.236:0.00942
474,GB_GCA_003141335.1:0.0478367)1.0:0.0430278)1.0:0.0325407,(((RS_GCF_000017625.1:0.0584
34,GB_GCA_002502735.1:0.0588348)0.951:0.0171407,GB_GCA_003170075.1:0.0586127)1.0:0.0580
206,GB_GCA_003136075.1:0.0956136)1.0:0.038382)'1.0:g__Methanoregula':0.0385914,(GB_GCA_0
02067895.1:0.121022,GB_GCA_903900025.1:0.099574)'1.0:g__MVRE01':0.0392511)1.0:0.106529,(((
(((((GB_GCA_002067095.1:0.0194965,GB_GCA_002501965.1:0.0231628)0.0:0.00543169,GB_GCA_0
02067155.1:0.0204641)1.0:0.0232184,GB_GCA_013331225.1:0.043074)1.0:0.0376929,((GB_GCA_00
2067485.1:0.0072674,GB_GCA_002067685.1:0.00960318)1.0:0.0374616,GB_GCA_002506105.1:0.05
78587)1.0:0.0515737)0.999:0.0411111,GB_GCA_000235685.2:0.140388)1.0:0.0400209,GB_GCA_002
509495.1:0.113406)'1.0:g__Methanolinea':0.0570146,(((GB_GCA_002068435.1:0.0116232,GB_GCA_
012516785.1:0.0259134)1.0:0.0812402,GB_GCA_013331415.1:0.100207)1.0:0.0447516,((GB_GCA_0
02499905.1:0.0342934,GB_GCA_013415575.1:0.0362279)0.998:0.0190414,GB_GCA_013415565.1:0.
0400418)1.0:0.07667)'1.0:g__UBA9949':0.0657571)0.993:0.0227428,((GB_GCA_001412335.1:0.0207
052,GB_GCA_002509485.1:0.0163855)'1.0:g__SD8':0.12803,GB_GCA_002503825.1:0.141542)0.824:
0.0289301)1.0:0.0447106,(GB_GCA_903819595.1:0.0442317,GB_GCA_903828975.1:0.0363318)'1.0:
g__CAIKOD01':0.161537)1.0:0.0383037)'0.974:f__Methanoregulaceae':0.0272722,((((((RS_GCF_0000
13445.1:0.0248297,GB_GCA_012520015.1:0.0792362)0.999:0.0189318,GB_GCA_012729995.1:0.046
8929)0.043:0.018632,(RS_GCF_003173335.1:0.0450061,GB_GCA_012729535.1:0.0666545)1.0:0.024
4551)1.0:0.111763,(RS_GCF_003173355.1:0.036325,GB_GCA_003251955.1:0.0269527)1.0:0.115149)
'1.0:g__Methanospirillum':0.0816886,GB_GCA_009779205.1:0.279802)1.0:0.108519,GB_GCA_90392
6075.1:0.299692)'1.0:f__Methanospirillaceae':0.0478177)1.0:0.0291019,((((GB_GCA_002495225.1:0.
0424669,GB_GCA_013331215.1:0.040578)1.0:0.0298919,GB_GCA_012728895.1:0.099352)0.349:0.0
318308,GB_GCA_004332335.1:0.0746054)'1.0:g__UBA288':0.156898,RS_GCF_000021965.1:0.17236
7)'1.0:f__Methanosphaerulaceae':0.0788882)1.0:0.0316909,(((((((RS_GCF_000015765.1:0.0131876,G
B_GCA_003315675.1:0.0171516)0.983:0.00502658,GB_GCA_002498375.1:0.00936922)1.0:0.016575
6,RS_GCF_002287215.1:0.0224602)1.0:0.0306643,GB_GCA_002506085.1:0.0652055)1.0:0.0698693,
GB_GCA_001940805.1:0.0735366)'1.0:g__Methanocorpusculum':0.207529,(((GB_GCA_002496395.1

:0.0276559,GB_GCA_013329165.1:0.0336518)1.0:0.0650684,(GB_GCA_003838065.1:0.0938902,GB_
GCA_003838185.1:0.106003)0.991:0.0294489)1.0:0.0438286,GB_GCA_007130455.1:0.107793)'1.0:g
__Methanocalculus':0.121766)'1.0:f__Methanocorpusculaceae':0.0899506,(((RS_GCF_000275865.1:
0.00817674,RS_GCF_013377755.1:0.0100122)1.0:0.0728691,(RS_GCF_001571385.1:0.0113433,GB_
GCA_002498315.1:0.00852649)1.0:0.10956)0.505:0.0227445,RS_GCF_004297185.1:0.0990446)'1.0:f
__Methanofollaceae;g__Methanofollis':0.150185)0.99:0.0319917)0.991:0.0251544,((((((((((((RS_GCF
_000015825.1:0.0174452,RS_GCF_000691865.1:0.0140722)0.999:0.0084702,(RS_GCF_001017125.1:
0.0176935,RS_GCF_001602375.1:0.029328)0.985:0.0078956)0.751:0.00436059,((GB_GCA_00131596
5.1:0.029441,RS_GCF_900095385.1:0.0145629)0.999:0.0109534,GB_GCA_012511245.1:0.0674982)0
.494:0.00970493)1.0:0.0185939,(GB_GCA_002498065.1:0.0247973,GB_GCA_002839575.1:0.042193)
0.867:0.00936961)1.0:0.0181417,(GB_GCA_002503885.1:0.0104864,GB_GCA_002839645.1:0.01262
25)1.0:0.0417882)1.0:0.0108285,(GB_GCA_002499455.1:0.0599194,GB_GCA_002506585.1:0.047625
4)0.981:0.0122695)0.944:0.00855168,GB_GCA_002508705.1:0.0600448)0.71:0.00883706,(GB_GCA_
001896715.1:0.0643833,GB_GCA_002501655.1:0.0438046)0.976:0.0142067)0.652:0.0102249,((((GB
_GCA_012510295.1:0.0191593,GB_GCA_012521035.1:0.0213116)1.0:0.0337997,GB_GCA_01251033
5.1:0.0601985)0.996:0.0182301,(GB_GCA_001512375.1:0.049692,GB_GCA_012797575.1:0.0648782)
1.0:0.0201076)0.93:0.0125885,RS_GCF_000304355.2:0.0242786)1.0:0.0413763)0.952:0.0164677,RS
_GCF_001571405.1:0.0676549)1.0:0.0264171,GB_GCA_002497965.1:0.104965)'1.0:g__Methanocull
eus':0.0633026,(GB_GCA_002503105.1:0.114853,RS_GCF_004102725.1:0.121897)0.505:0.0257618)
1.0:0.0608187,GB_GCA_002503135.1:0.219956)'1.0:f__Methanoculleaceae':0.0431248)0.933:0.0273
481,(((((RS_GCF_000147875.1:0.00844538,RS_GCF_000784355.1:0.00647498)'1.0:g__Methanolacini
a':0.130992,RS_GCF_000243255.1:0.176862)0.576:0.0299498,RS_GCF_000711215.1:0.202487)1.0:0.
079078,(((RS_GCF_001315945.1:0.0404425,RS_GCF_009914725.1:0.0201811)1.0:0.0362322,GB_GC
A_012523385.1:0.08853)1.0:0.103961,GB_GCA_011045995.1:0.150496)'1.0:g__Methanogenium':0.0
754607)1.0:0.0618843,((GB_GCA_001412355.1:0.0997286,GB_GCA_013415665.1:0.0862388)'1.0:g_
_LKUD01':0.0796084,GB_GCA_011048835.1:0.171556)0.966:0.0327977)'0.999:f__Methanomicrobia
ceae':0.0379516)'1.0:c__Methanomicrobia;o__Methanomicrobiales':0.297712)0.975:0.048582,(((GB
_GCA_003139855.1:0.00438252,GB_GCA_003158275.1:0.00326531)1.0:0.0351743,GB_GCA_003162
175.1:0.036974)1.0:0.0667672,GB_GCA_003170935.1:0.101111)'1.0:c__Bog-38;o__Bog-38;f__Bog-
38;g__Bog-
38':0.364559)1.0:0.0472208,(((((((((((((RS_GCF_000504205.1:0.0251082,RS_GCF_006546655.1:0.01
80278)0.583:0.00774592,RS_GCF_900100715.1:0.0132918)1.0:0.0419287,RS_GCF_900114835.1:0.0
449738)1.0:0.0181932,(RS_GCF_002243045.1:0.0517842,GB_GCA_002507205.1:0.041706)0.923:0.0
143282)0.966:0.0165825,GB_GCA_003565715.1:0.109311)1.0:0.0534273,(((GB_GCA_002508635.1:0
.00994431,RS_GCF_013388255.1:0.00357187)1.0:0.0493675,RS_GCF_004745425.1:0.0518814)1.0:0.
0435148,(GB_GCA_000306725.1:0.0394561,GB_GCA_002501695.1:0.0373623)1.0:0.0619929)1.0:0.0
344768)'1.0:g__Methanolobus':0.0613878,((((GB_GCA_001896725.1:0.0533307,GB_GCA_00206727
5.1:0.0197276)0.901:0.00926906,RS_GCF_000328665.1:0.0429821)0.995:0.00968247,(GB_GCA_002
067265.1:0.0255638,GB_GCA_002508425.1:0.0183846)1.0:0.0198293)1.0:0.0287231,GB_GCA_0143
61205.1:0.0651792)'1.0:g__Methanomethylovorans':0.129277)1.0:0.0363401,(((((RS_GCF_00002586
5.1:0.0225267,RS_GCF_001889405.1:0.0131634)0.995:0.00410927,(RS_GCF_002761295.1:0.009026
37,RS_GCF_900215215.1:0.0157714)0.713:0.0015935)0.903:0.00247994,RS_GCF_004137855.1:0.01
25716)0.877:0.00569043,RS_GCF_003722075.1:0.0107015)'1.0:g__Methanohalophilus':0.211416,((((
RS_GCF_000765475.1:0.0142922,RS_GCF_900774055.1:0.0130194)0.911:0.00603204,RS_GCF_9001
11645.1:0.0129704)1.0:0.0227932,RS_GCF_000970325.1:0.0376659)1.0:0.0345107,RS_GCF_000013
725.1:0.0823104)'1.0:g__Methanococcoides':0.0860013)1.0:0.0370851)0.999:0.0274772,(((RS_GCF_
000217995.1:0.0191351,GB_GCA_007127385.1:0.0213148)1.0:0.0803479,GB_GCA_003555325.1:0.1
72125)'1.0:g__Methanosalsum':0.103661,RS_GCF_000196655.1:0.228452)0.997:0.0360049)1.0:0.03

74949,((((((((((RS_GCF_000195895.1:0.0168197,RS_GCF_000970025.1:0.0126657)1.0:0.00923354,R
S_GCF_000969905.1:0.0164425)0.999:0.0100819,GB_GCA_002505485.1:0.0227751)0.303:0.005314
28,((RS_GCF_000970305.1:0.00491895,GB_GCA_013331275.1:0.00830992)0.645:0.00433488,RS_GC
F_002287235.1:0.0111229)1.0:0.0146545)1.0:0.0112229,GB_GCA_002496565.1:0.0268227)1.0:0.01
31496,(GB_GCA_002499445.1:0.0179221,GB_GCA_002509325.1:0.017646)1.0:0.035105)0.811:0.004
95212,((RS_GCF_000969885.1:0.0277508,RS_GCF_001304615.2:0.0159017)1.0:0.0272803,RS_GCF_0
04099695.1:0.0298462)0.989:0.00833093)1.0:0.0212801,((GB_GCA_003157235.1:0.020017,GB_GCA
_003164755.1:0.0226333)1.0:0.069281,GB_GCA_001714685.2:0.0351635)1.0:0.021841)1.0:0.01730
97,((((RS_GCF_000970265.1:0.026587,RS_GCF_000979455.1:0.0188157)1.0:0.0116785,RS_GCF_000
969965.1:0.0257038)1.0:0.0189083,(RS_GCF_000007345.1:0.0175025,RS_GCF_000970085.1:0.0184
298)1.0:0.0205774)0.436:0.00798629,(((RS_GCF_000970285.1:0.0144809,GB_GCA_013330315.1:0.0
273201)0.334:0.00670409,RS_GCF_000970205.1:0.0354619)0.91:0.00547759,GB_GCA_013331265.1
:0.0367179)0.998:0.0103073)1.0:0.0188984)'1.0:g__Methanosarcina':0.0482774,(RS_GCF_00097004
5.1:0.0177044,GB_GCA_013329415.1:0.0130038)'1.0:g__MTP4':0.0684108)1.0:0.110762,((((GB_GCA
_009783635.1:0.049067,GB_GCA_009784005.1:0.0536123)1.0:0.0396212,RS_GCF_004363215.1:0.0
586516)1.0:0.0413645,GB_GCA_009776675.1:0.103722)1.0:0.112564,GB_GCA_012518265.1:0.2567
39)'1.0:g__Methanimicrococcus':0.149476)1.0:0.0670267)0.365:0.0290955,GB_GCA_012329085.1:0.
338108)'1.0:f__Methanosarcinaceae':0.0901125,((((((((RS_GCF_900196725.1:0.0234023,GB_GCA_9
02386135.1:0.0231226)1.0:0.0604161,RS_GCF_000685155.1:0.105573)0.928:0.0170057,((GB_GCA_0
12026835.1:0.0251075,GB_GCA_902386205.1:0.0205274)1.0:0.0635376,GB_GCA_902386115.1:0.12
3287)0.959:0.0143634)0.409:0.00795497,GB_GCA_902386085.1:0.109735)0.995:0.0184046,((((RS_G
CF_002487355.1:0.0184325,GB_GCA_902384525.1:0.0108147)0.928:0.00560775,GB_GCA_0120267
95.1:0.0203246)1.0:0.0258271,GB_GCA_003104905.1:0.0538706)1.0:0.0662661,GB_GCA_90238625
5.1:0.0745645)1.0:0.0401147)'1.0:g__Methanoperedens':0.0471815,GB_GCA_002839545.1:0.15016
9)1.0:0.109603,GB_GCA_013331375.1:0.278735)'1.0:f__Methanoperedenaceae':0.0646476,(((GB_G
CA_004193545.1:0.0149489,GB_GCA_004212095.1:0.0319521)0.741:0.0143164,GB_GCA_01291072
5.1:0.0256381)'1.0:g__Ethanoperedens':0.174389,(GB_GCA_002254825.2:0.00211058,GB_GCA_002
255135.1:5e-09)'1.0:g__EX4572-44':0.198094)'1.0:f__EX4572-
44':0.166258)0.963:0.0361903,((((GB_GCA_009649835.1:0.00347343,GB_GCA_013374465.1:0.0035
8841)1.0:0.0154494,(GB_GCA_013330355.1:0.0387142,GB_GCA_014237145.1:0.0281802)0.0:0.0066
2936)'1.0:g__UBA7939':0.0920551,GB_GCA_013374385.1:0.128046)1.0:0.0359749,GB_GCA_002926
195.1:0.165398)'1.0:f__HR1':0.168597)0.97:0.0400551)1.0:0.0475419,((((((GB_GCA_003194445.1:0.
0175237,GB_GCA_003661105.1:0.0226902)1.0:0.0222006,GB_GCA_013572355.1:0.0228545)0.78:0.
011768,GB_GCA_002503595.1:0.0195349)1.0:0.0869472,GB_GCA_002506015.1:0.139284)0.805:0.0
202779,GB_GCA_003601535.1:0.0982964)0.884:0.0262522,(GB_GCA_004211975.1:0.092623,GB_GC
A_012961645.1:0.0591391)1.0:0.0668281)'1.0:f__ANME-
2c;g__UBA203':0.261997)'0.844:o__Methanosarcinales':0.0372495,((((((((((GB_GCA_002067705.1:0.
0556192,GB_GCA_011391755.1:0.04193)1.0:0.0320844,(GB_GCA_002256595.1:0.0300457,GB_GCA
_002505805.1:0.0500789)1.0:0.0473834)1.0:0.0239729,((RS_GCF_000204415.1:0.00697554,GB_GCA
_011620785.1:0.00965257)1.0:0.0820758,GB_GCA_903871465.1:0.0585461)1.0:0.020258)0.994:0.0
13437,GB_GCA_902385025.1:0.0677067)0.982:0.0168419,GB_GCA_003857025.1:0.0752599)0.771:0
.0141172,(GB_GCA_002067755.1:0.0192371,GB_GCA_012798025.1:0.0171855)1.0:0.0574184)1.0:0.
0273047,GB_GCA_002067365.1:0.0910775)'1.0:g__Methanothrix':0.0388992,GB_GCA_013178225.1
:0.125025)0.999:0.030634,(GB_GCA_002506335.1:0.175688,GB_GCA_003162615.1:0.102459)0.554:
0.0237688)1.0:0.0598977,((RS_GCF_000014945.1:0.153863,GB_GCA_002067795.1:0.169825)1.0:0.0
442739,GB_GCA_013415595.1:0.149271)1.0:0.0324992)1.0:0.0657782,(((((RS_GCF_000235565.1:0.0
405154,GB_GCA_001602645.1:0.0321311)1.0:0.0366283,GB_GCA_002506535.1:0.0720829)1.0:0.01
94136,(GB_GCA_002502785.1:0.0308595,GB_GCA_013329455.1:0.0501724)1.0:0.0159844)0.0:0.008

75384,GB_GCA_001509375.1:0.0408796)'1.0:g__Methanothrix_A':0.101161,GB_GCA_002501765.1:
0.155891)1.0:0.071285)'1.0:o__Methanotrichales;f__Methanotrichaceae':0.201354)'1.0:c__Methan
osarcinia':0.038872,(((RS_GCF_000011005.1:0.0778381,RS_GCF_000251105.1:0.0825112)'1.0:g__M
ethanocella':0.0665259,(RS_GCF_000063445.1:0.102753,GB_GCA_002067315.1:0.101779)'1.0:g__M
ethanocella_A':0.0554144)'1.0:f__Methanocellaceae':0.290332,(GB_GCA_002495885.1:0.0944905,G
B_GCA_013415795.1:0.0888594)'1.0:f__UBA148;g__UBA148':0.233087)'1.0:c__Methanocellia;o__M
ethanocellales':0.0590125)0.937:0.0259008)1.0:0.043361,(((((((((GB_GCA_003160755.1:0.0291856,
GB_GCA_013180605.1:0.0307098)1.0:0.0709281,GB_GCA_003601795.1:0.0453194)0.0:0.0398245,G
B_GCA_003661125.1:0.0813059)'1.0:g__QEXZ01':0.0355029,GB_GCA_003194425.1:0.208185)0.985:
0.0218372,(((GB_GCA_013180585.1:0.0744149,GB_GCA_013374505.1:0.106326)'1.0:g__QENJ01':0.
0703783,GB_GCA_003194435.1:0.19202)1.0:0.0402988,GB_GCA_009618475.1:0.0977082)0.97:0.01
99704)1.0:0.0267224,(((GB_GCA_013139985.1:0.00378863,GB_GCA_013180565.1:0.0147807)0.999:
0.0125495,GB_GCA_009903405.1:0.0148372)1.0:0.0180835,GB_GCA_003336485.1:0.0390674)'1.0:g
__QENH01':0.143463)0.05:0.0262025,((GB_GCA_011049045.1:0.180981,GB_GCA_014061035.1:0.18
3477)'0.983:g__JACGMN01':0.0361035,GB_GCA_004212135.1:0.141027)0.999:0.0265266)'1.0:f__AN
ME-1':0.181352,GB_GCA_003661185.1:0.251007)'1.0:o__ANME-
1':0.138768,(GB_GCA_001766815.1:0.158002,GB_GCA_001766825.1:0.111357)'1.0:o__Syntropharc
haeales;f__Syntropharchaeaceae':0.2565)'1.0:c__Syntropharchaeia':0.0620878,((GB_GCA_00201007
5.1:0.0114246,GB_GCA_003661015.1:0.0168082)'1.0:g__JdFR-
19':0.235604,RS_GCF_000711905.1:0.261955)'1.0:c__Methanosarcinia_A;o__Methanosarcinales_A;f
__Methermicoccaceae':0.180144)0.692:0.0262125)1.0:0.0364975,(((GB_GCA_004212085.1:0.02878
3,RS_GCF_902158735.1:0.0387785)0.69:0.0127235,RS_GCF_902158745.1:0.0227062)'1.0:g__Metha
nolliviera':0.196169,(GB_GCA_004212075.1:0.0892877,GB_GCA_013329575.1:0.0864341)'1.0:g__M
ethanoliparum':0.228022)'1.0:c__Methanoliparia;o__Methanoliparales;f__Methanoliparaceae':0.27
2595)0.66:0.027135,(((((((((((GB_GCA_004347825.1:0.0329308,GB_GCA_011605865.1:0.0192273)1.
0:0.0418069,GB_GCA_011048195.1:0.0657616)1.0:0.0595939,GB_GCA_011367245.1:0.102893)1.0:0
.0391404,(GB_GCA_004347865.1:0.052195,GB_GCA_011361745.1:0.0609301)1.0:0.0720032)'1.0:g_
_WYZ-
LMO2':0.140567,((RS_GCF_000008665.1:0.0319964,GB_GCA_011333375.1:0.0623481)1.0:0.052803
6,GB_GCA_002507545.1:0.0850914)'1.0:g__Archaeoglobus':0.0513061)0.334:0.026344,GB_GCA_00
2010285.1:0.138179)1.0:0.0610729,(RS_GCF_000025285.1:0.0561302,GB_GCA_003663025.1:0.1277
26)'1.0:g__Archaeoglobus_B':0.131592)0.801:0.0235002,GB_GCA_003662995.1:0.161603)0.679:0.0
260282,((((((GB_GCA_002010195.1:0.108987,GB_GCA_002494625.1:0.0824605)0.999:0.0404082,RS
_GCF_000385565.1:0.143069)0.0:0.0173709,(GB_GCA_002011235.1:0.0391171,GB_GCA_00366298
5.1:0.0305568)1.0:0.0841869)1.0:0.0423471,GB_GCA_002011215.1:0.128421)'1.0:g__Archaeoglobu
s_A':0.103733,GB_GCA_002010215.1:0.169361)0.938:0.0236471,(((RS_GCF_000194625.1:0.0461172
,GB_GCA_003663055.1:0.0697092)1.0:0.0455058,GB_GCA_003978865.1:0.228138)'1.0:g__Archaeog
lobus_C':0.0441113,GB_GCA_002010045.1:0.242162)0.337:0.0222641)0.474:0.0223705)0.996:0.027
3074,((((RS_GCF_000789255.1:0.103768,GB_GCA_002494725.1:0.10955)0.689:0.0216469,RS_GCF_0
01006045.1:0.0620881)1.0:0.0671804,GB_GCA_011357905.1:0.115171)'1.0:g__Geoglobus':0.057257
3,RS_GCF_000025505.1:0.144981)1.0:0.0543584)'1.0:f__Archaeoglobaceae':0.154502,GB_GCA_002
010305.1:0.298856)'1.0:o__Archaeoglobales':0.128861,(GB_GCA_002011165.1:0.094481,GB_GCA_0
14361165.1:0.0866129)'1.0:o__JdFR-21;f__JdFR-21;g__JdFR-
21':0.27066)'1.0:c__Archaeoglobi':0.0493863)1.0:0.0536308,((GB_GCA_001914405.1:0.259455,GB_
GCA_003034855.1:0.277486)1.0:0.0770642,(RS_GCF_002153915.1:0.102506,GB_GCA_004212035.1:
0.0929235)'1.0:f__Methanonatronarchaeaceae;g__Methanonatronarchaeum':0.193684)'1.0:c__Met
hanonatronarchaeia;o__Methanonatronarchaeales':0.279063)'1.0:p__Halobacteriota':0.0602183,((((
(((((((((((((((((((((((GB_GCA_001628485.1:0.00842774,GB_GCA_902625325.1:0.0225347)0.987:0.00

51975,(GB_GCA_001629255.1:0.0203795,GB_GCA_902601345.1:0.0165245)0.991:0.00595404)0.408
:0.000898647,GB_GCA_001629235.1:0.00765833)0.913:0.00404766,GB_GCA_902573015.1:0.01345
51)0.907:0.0063084,GB_GCA_902573085.1:0.0359306)0.999:0.0181972,GB_GCA_002697065.1:0.03
5329)1.0:0.0502846,(((((GB_GCA_002723045.1:0.0288256,GB_GCA_002730445.1:0.0273731)0.0:0.0
0590663,GB_GCA_003602515.1:0.0382077)0.996:0.0141108,GB_GCA_002457155.1:0.0298823)0.36
8:0.00892884,GB_GCA_002683155.1:0.0726521)1.0:0.0226726,(GB_GCA_004195615.1:0.0308037,G
B_GCA_014239955.1:0.0263549)1.0:0.0383012)1.0:0.0372256)1.0:0.0396552,GB_GCA_002718695.1
:0.113068)0.786:0.0132174,(((GB_GCA_002172185.1:0.0285477,GB_GCA_002720055.1:0.0333807)0
.983:0.0162013,GB_GCA_002713185.1:0.0302613)1.0:0.092289,(GB_GCA_002506755.1:0.0265515,
GB_GCA_902573115.1:0.0184696)1.0:0.10545)0.987:0.0257419)0.381:0.013373,GB_GCA_00271608
5.1:0.106845)1.0:0.0330628,GB_GCA_002725315.1:0.109833)0.946:0.0231804,GB_GCA_002728565.
1:0.172887)0.95:0.0266304,GB_GCA_002722735.1:0.143135)0.886:0.0285989,(GB_GCA_001629205.
1:0.0387573,GB_GCA_002496485.1:0.0376869)1.0:0.16573)1.0:0.0740852,((((((GB_GCA_002712575.
1:0.0123453,GB_GCA_002719615.1:0.0214622)0.999:0.0091236,GB_GCA_003602595.1:0.0228199)0
.999:0.0123727,GB_GCA_002172375.1:0.0240142)1.0:0.0287124,GB_GCA_002731195.1:0.0425028)
0.997:0.0236284,GB_GCA_002457145.1:0.0576836)1.0:0.0595583,GB_GCA_002503285.1:0.110517)
1.0:0.13887)'1.0:g__MGIIb-
O3':0.0446546,(((((((GB_GCA_002499325.1:0.0212397,GB_GCA_002499865.1:0.0169053)0.0:0.0015
4507,(GB_GCA_002501805.1:0.0116289,GB_GCA_002502095.1:0.0125696)0.189:0.00141024)1.0:0.0
237894,(GB_GCA_002499345.1:0.0255085,GB_GCA_002506825.1:0.0145882)1.0:0.0272668)1.0:0.04
06066,GB_GCA_002496725.1:0.0708482)0.902:0.0197467,((GB_GCA_002501605.1:0.0168588,GB_G
CA_002505775.1:0.0128702)1.0:0.0416674,GB_GCA_002730095.1:0.0398221)1.0:0.0389433)1.0:0.0
7404,(((GB_GCA_002497985.1:0.00628572,GB_GCA_002719635.1:0.0106556)0.991:0.00874032,GB_
GCA_002726275.1:0.0157855)1.0:0.0401666,((GB_GCA_002498925.1:0.0330413,GB_GCA_00271899
5.2:0.0220143)1.0:0.0324471,GB_GCA_002504435.1:0.0401572)1.0:0.0242587)1.0:0.0642022)'1.0:g
__MGIIb-
O5':0.102471,(((((GB_GCA_002457605.1:0.0192242,GB_GCA_002505325.1:0.0122833)1.0:0.017934,
(GB_GCA_002495735.1:0.0189674,GB_GCA_004195715.1:0.017981)1.0:0.0171025)1.0:0.0184705,G
B_GCA_014240285.1:0.04011)1.0:0.0452643,((((GB_GCA_002501885.1:0.0172534,GB_GCA_002503
055.1:0.0291793)1.0:0.0297261,GB_GCA_002727275.1:0.0458974)0.979:0.0114722,GB_GCA_00271
4745.1:0.04461)0.336:0.0116501,GB_GCA_002698225.1:0.0546284)1.0:0.0930849)0.919:0.0352982,
(GB_GCA_002507125.1:0.0657138,GB_GCA_003193925.1:0.0656939)1.0:0.0402724)'1.0:g__Thalass
archaeum':0.084574)0.885:0.0233462)0.997:0.0233782,(((((((GB_GCA_002498985.1:0.00535827,GB
_GCA_002730775.1:0.0214672)0.574:0.00152655,GB_GCA_002685315.1:0.00634558)1.0:0.0134167
,GB_GCA_002495525.1:0.0279697)1.0:0.0187926,((GB_GCA_002497245.1:0.0220356,GB_GCA_9025
93945.1:0.018084)1.0:0.016227,GB_GCA_002504905.1:0.0373814)0.852:0.00798499)0.994:0.01492
36,GB_GCA_002686525.1:0.0530044)1.0:0.0406288,((GB_GCA_002494975.1:0.0140479,GB_GCA_90
2511005.1:0.0192505)0.666:0.00971556,GB_GCA_902514485.1:0.0184079)1.0:0.0484782)1.0:0.102
537,(GB_GCA_002499785.1:0.0270225,GB_GCA_012960975.1:0.0341156)1.0:0.112941)'1.0:g__MGII
b-
O2':0.0568129)1.0:0.0394943,(((((((GB_GCA_002497025.1:0.00487941,GB_GCA_902560445.1:0.018
7981)0.994:0.00882062,GB_GCA_002457555.1:0.0232008)1.0:0.0230174,((GB_GCA_002496905.1:0.
0235007,GB_GCA_002502175.1:0.0122212)0.97:0.00514385,GB_GCA_002685415.1:0.0242459)0.99
9:0.0130463)0.998:0.0152198,(GB_GCA_002502365.1:0.00868724,GB_GCA_002708385.1:0.0239327
)1.0:0.046826)1.0:0.0519989,(GB_GCA_002497895.1:0.0148449,GB_GCA_013329905.1:0.0224294)1
.0:0.0496935)1.0:0.0410359,((GB_GCA_002457595.1:0.045681,GB_GCA_013330385.1:0.0355869)1.
0:0.0487808,GB_GCA_002498725.1:0.131211)0.774:0.0205439)1.0:0.0323659,GB_GCA_002498525.
1:0.121817)'1.0:g__MGIIb-

O1':0.080613)1.0:0.075821,((((((GB_GCA_002170775.2:0.0276834,GB_GCA_002712645.1:0.0099083
8)0.705:0.00501318,GB_GCA_002497295.1:0.022795)0.992:0.00653249,GB_GCA_002504845.1:0.01
1895)0.693:0.00811713,GB_GCA_002717835.1:0.0436275)0.992:0.0158699,GB_GCA_002505455.1:0
.0277382)1.0:0.0758291,((((GB_GCA_002505695.1:0.0148568,GB_GCA_002507175.1:0.0210146)0.9
9:0.010347,GB_GCA_002495675.1:0.0256069)1.0:0.0524162,GB_GCA_011523055.1:0.0665239)1.0:0
.06814,GB_GCA_002499585.1:0.124083)0.997:0.0352526)'1.0:g__MGIIb-
N1':0.202336)1.0:0.092646,(((((((GB_GCA_002502625.1:0.0220828,GB_GCA_002708015.1:0.011972
3)0.879:0.00587013,GB_GCA_002702985.1:0.0184216)0.324:0.00469412,GB_GCA_002697105.1:0.0
223968)1.0:0.0247896,GB_GCA_002708695.1:0.0493151)0.526:0.0138988,GB_GCA_002503045.1:0.
0405999)1.0:0.041621,(GB_GCA_002503665.1:0.0154316,GB_GCA_002718215.1:0.0135973)1.0:0.06
09224)1.0:0.0828575,(((GB_GCA_002497905.1:0.016199,GB_GCA_002506875.1:0.0100078)0.994:0.
0121071,GB_GCA_002713585.1:0.0220828)1.0:0.0333888,GB_GCA_002712285.1:0.032889)1.0:0.12
3102)'1.0:g__MGIIb-
N2':0.208435)1.0:0.0583574,(((((GB_GCA_002685895.1:0.068611,GB_GCA_002725475.1:0.0773223)
0.991:0.0256302,GB_GCA_002727675.1:0.111308)0.65:0.0274228,((GB_GCA_012959435.1:0.033527
9,GB_GCA_012964335.1:0.0324651)0.385:0.0148823,GB_GCA_014384685.1:0.0326697)1.0:0.08922
59)1.0:0.0881865,GB_GCA_002505495.1:0.163275)'1.0:g__MGIIb-
Q1':0.139017,(((((GB_GCA_002498455.1:0.0151137,GB_GCA_002724815.1:0.0140771)1.0:0.020337
2,GB_GCA_002701965.1:0.0283995)0.999:0.0250782,GB_GCA_002505685.1:0.0444011)1.0:0.04051
8,GB_GCA_002457195.1:0.08657)0.985:0.0326603,GB_GCA_002722595.1:0.101532)'1.0:g__MGIIb-
P':0.166946)0.928:0.0433849)'1.0:f__Thalassarchaeaceae':0.0681877,((((((((((((((((GB_GCA_0027210
85.1:5e-
09,GB_GCA_013329355.1:0.0010867)1.0:0.0127159,GB_GCA_013329865.1:0.00791115)1.0:0.02844
18,GB_GCA_002506025.1:0.0294877)1.0:0.0208265,GB_GCA_002505355.1:0.0637332)0.981:0.0128
976,GB_GCA_002496955.1:0.0772805)0.592:0.0115067,GB_GCA_013329295.1:0.0476424)0.186:0.0
0819321,(GB_GCA_002495945.1:0.0533961,GB_GCA_011526375.1:0.0452957)0.456:0.00835795)1.0
:0.0300197,(((GB_GCA_002726395.1:0.0172153,GB_GCA_009887195.1:0.0219938)0.992:0.0070514
4,GB_GCA_002688825.1:0.0333021)0.833:0.00580382,GB_GCA_013329925.1:0.0140026)1.0:0.0625
294)1.0:0.0686031,(((GB_GCA_002172355.2:0.011555,GB_GCA_002697705.1:0.0168059)0.961:0.01
05145,GB_GCA_003602575.1:0.0249089)1.0:0.0492844,((GB_GCA_002702945.1:0.0321023,GB_GCA
_002729095.1:0.039727)0.809:0.0091627,GB_GCA_002727485.1:0.0688341)1.0:0.0308975)1.0:0.05
62304)1.0:0.0528613,((((((GB_GCA_002499705.1:0.0109908,GB_GCA_902520595.1:0.0182509)0.989
:0.00953168,GB_GCA_002170315.2:0.0233043)1.0:0.0268986,GB_GCA_009937025.1:0.0425292)1.0:
0.0228241,((GB_GCA_002506275.1:0.0291766,GB_GCA_003602605.1:0.0266489)1.0:0.0215682,GB_
GCA_002687075.1:0.0696828)1.0:0.0174048)1.0:0.0340514,GB_GCA_002499545.1:0.0769742)1.0:0.
0390616,((((GB_GCA_000246735.1:0.0170994,GB_GCA_009887095.1:0.0145221)1.0:0.0239408,GB_
GCA_013330285.1:0.0317721)0.989:0.0136669,((GB_GCA_002502605.1:0.011242,GB_GCA_0036026
45.1:0.0148001)1.0:0.0275163,GB_GCA_002495535.1:0.0343344)0.986:0.0134388)0.871:0.0119855,
GB_GCA_013329255.1:0.0560554)1.0:0.0489533)1.0:0.0501436)0.292:0.0195992,((GB_GCA_002499
015.1:0.0311765,GB_GCA_009937205.1:0.0199515)1.0:0.0396553,GB_GCA_012959845.1:0.0385888
)1.0:0.138413)'0.996:g__MGIIa-
L1':0.0288327,((((((((GB_GCA_002704515.1:0.0115905,GB_GCA_013330155.1:0.0104136)0.997:0.01
11927,GB_GCA_002714305.1:0.0225163)0.999:0.0150365,GB_GCA_002502295.1:0.0368682)1.0:0.0
320249,((GB_GCA_002694525.1:0.0332268,GB_GCA_002722695.1:0.0449969)1.0:0.0289038,GB_GC
A_902625885.1:0.0826686)0.744:0.0111692)0.418:0.00843372,(GB_GCA_002497765.1:0.0378094,G
B_GCA_002725275.1:0.0389805)1.0:0.0170137)0.973:0.0148115,(GB_GCA_002495645.1:0.0297156,
GB_GCA_002689105.1:0.0277849)1.0:0.0275932)0.999:0.0242433,GB_GCA_002728035.1:0.0777529
)1.0:0.0319297,((GB_GCA_002494645.1:0.0227906,GB_GCA_003602485.1:0.0299354)1.0:0.0660121

,(GB_GCA_002505405.1:0.0443528,GB_GCA_002726495.1:0.0526847)1.0:0.0693909)0.246:0.017993
8)0.973:0.0227208,(GB_GCA_003602475.1:0.0703994,GB_GCA_011524855.1:0.0730076)1.0:0.06504
22)'1.0:g__Poseidonia':0.0953448)0.997:0.0393456,((((((GB_GCA_002499195.1:0.0480919,GB_GCA
_002706065.1:0.0443677)0.198:0.00715269,(GB_GCA_002502215.1:0.0167092,GB_GCA_002698145
.1:0.0144743)1.0:0.0359713)0.998:0.0131831,((GB_GCA_002692685.1:0.0290252,GB_GCA_0122702
65.1:0.00929631)0.889:0.00587828,GB_GCA_013330015.1:0.018871)1.0:0.0261946)0.0:0.00745992,
GB_GCA_002719815.1:0.0443693)0.912:0.0118657,(GB_GCA_002722615.1:0.0233453,GB_GCA_013
911465.1:0.0107203)1.0:0.0277628)0.904:0.017869,GB_GCA_002726845.1:0.0609961)1.0:0.050014
4,((GB_GCA_002171315.2:0.0103974,GB_GCA_002496635.1:0.0110068)0.967:0.00936453,(GB_GCA
_003602665.1:0.0138542,GB_GCA_009887135.1:0.0171687)0.998:0.0140461)1.0:0.0617576)'1.0:g_
_MGIIa-
L2':0.146355)0.941:0.0304301,((((((GB_GCA_002496845.1:0.0070735,GB_GCA_002723635.1:0.0108
405)1.0:0.0161236,GB_GCA_003602635.1:0.0150793)1.0:0.01924,((GB_GCA_002694585.1:0.009450
18,GB_GCA_002722135.1:0.0104802)0.954:0.0093833,GB_GCA_002731395.1:0.0304789)1.0:0.0222
957)0.999:0.0138271,GB_GCA_013330765.1:0.0433904)0.284:0.0095978,GB_GCA_013330925.1:0.0
211636)'1.0:g__MGIIa-L3':0.191478,(GB_GCA_002495405.1:0.00086064,GB_GCA_002718195.1:5e-
09)'1.0:g__MGIIa-
L4':0.218916)1.0:0.0568042)1.0:0.0618887,(((((((((GB_GCA_002498185.1:0.00713257,GB_GCA_002
727695.1:0.0104223)0.997:0.00887558,(GB_GCA_002709705.1:0.0235343,GB_GCA_002710205.1:0.
0209712)0.143:0.00484792)0.985:0.00793411,((GB_GCA_013330625.1:0.00805762,GB_GCA_90256
3095.1:0.00317383)0.944:0.00298474,GB_GCA_002715725.1:0.0106403)1.0:0.0299529)1.0:0.01322
37,((((GB_GCA_002506705.1:0.00837406,GB_GCA_011525095.1:0.0087056)1.0:0.0291667,GB_GCA_
011522915.1:0.0300795)1.0:0.0165289,GB_GCA_002720115.1:0.0798971)0.265:0.00734115,GB_GC
A_002689565.1:0.0470386)0.902:0.00633485)1.0:0.0260858,(((GB_GCA_002701145.1:0.00819029,G
B_GCA_902605365.1:0.00766877)1.0:0.0622249,(GB_GCA_003602535.1:0.0352978,GB_GCA_00993
6765.1:0.045785)1.0:0.0243432)1.0:0.019,(GB_GCA_002689345.1:0.0523949,GB_GCA_003602415.1:
0.0446982)0.403:0.00930193)0.991:0.0147414)0.971:0.0169704,GB_GCA_002694245.1:0.0752529)0
.221:0.0150396,(GB_GCA_002507425.1:0.0647944,GB_GCA_002710015.1:0.0416118)1.0:0.0646182)
0.998:0.0282877,(GB_GCA_002707335.2:0.0124327,GB_GCA_002727515.1:0.00672469)1.0:0.12679
4)0.991:0.0285971,(GB_GCA_002706615.1:0.111948,GB_GCA_004195515.1:0.135876)0.441:0.02484
21)'1.0:g__MGIIa-
K1':0.0814883,(GB_GCA_002699425.1:0.0302303,GB_GCA_002719395.1:0.0236741)'1.0:g__MGIIa-
K2':0.253842)0.999:0.0468595)1.0:0.070649,(((GB_GCA_002505935.1:0.00995706,GB_GCA_002719
475.1:0.014111)1.0:0.11925,(GB_GCA_012959275.1:0.026964,GB_GCA_014240385.1:0.0288256)1.0:
0.12471)'1.0:g__UBA226':0.131186,(GB_GCA_002503395.1:0.213125,GB_GCA_004195635.1:0.2032
58)1.0:0.0624656)0.991:0.0429799)1.0:0.0580913,((((GB_GCA_002498205.1:0.031633,GB_GCA_002
696315.1:0.0355354)0.997:0.0238855,(GB_GCA_002692465.1:0.014069,GB_GCA_002720275.1:0.01
55913)1.0:0.0402858)1.0:0.0411447,GB_GCA_002699515.1:0.0888002)0.99:0.0347402,GB_GCA_002
720095.2:0.100259)'1.0:g__MGIIa-
I':0.285772)'0.976:f__Poseidoniaceae':0.046424)1.0:0.113882,GB_GCA_002715545.1:0.428696)'1.0:c
__Poseidoniia_A;o__Poseidoniales':0.250436,((RS_GCF_012044895.1:0.235728,GB_GCA_013041945
.1:0.263265)'1.0:g__JABDRC01':0.367313,GB_GCA_013288705.1:0.411515)'1.0:c__JABDBI01;o__JAB
DBI01;f__JABDBI01':0.628659)0.947:0.121151,(((((((GB_GCA_902531985.1:0.0373194,GB_GCA_9026
27615.1:0.0406176)1.0:0.0363887,GB_GCA_902533295.1:0.0601448)1.0:0.0421621,(GB_GCA_01424
0055.1:0.0434605,GB_GCA_014240255.1:0.0292903)1.0:0.0661757)0.64:0.0236281,GB_GCA_00250
6485.1:0.113502)0.738:0.0286184,((((GB_GCA_001875345.1:0.022298,GB_GCA_001875365.1:0.034
3205)0.963:0.0113957,GB_GCA_002724775.1:0.0404459)0.996:0.0168193,GB_GCA_002718915.1:0.
044224)0.553:0.00831803,GB_GCA_002497685.1:0.040953)1.0:0.0923986)'1.0:g__CG-

Epi1':0.173825,(((GB_GCA_002731655.1:0.0191572,GB_GCA_003193815.1:0.00949238)0.992:0.0151
054,GB_GCA_002509165.1:0.0354269)1.0:0.0537924,(GB_GCA_002687355.1:0.0339542,GB_GCA_0
02731905.1:0.0290307)1.0:0.0558603)'1.0:g__UBA8886':0.216545)1.0:0.0846971,(GB_GCA_001875
425.1:0.0286046,GB_GCA_002509225.1:0.0381016)'1.0:g__UBA102':0.298744)'1.0:c__Poseidoniia_B
;o__MGIII;f__CG-
Epi1':0.291604)1.0:0.0953987,(GB_GCA_002254385.1:0.424764,GB_GCA_002254765.1:0.400033)'0.
999:c__EX4484-6;o__EX4484-
6':0.12578)0.728:0.0419615,((((((((((((((((RS_GCF_001560915.1:0.0106456,RS_GCF_902787415.1:0.
018838)1.0:0.0588306,RS_GCF_902763685.1:0.0546877)1.0:0.0854297,RS_GCF_000350305.1:0.153
07)0.622:0.021035,((RS_GCF_000300255.2:0.0337907,GB_GCA_002503545.1:0.0490904)1.0:0.04292
24,RS_GCF_001481295.1:0.14354)1.0:0.0372477)1.0:0.0395793,GB_GCA_002495325.1:0.140009)'1.
0:g__Methanomethylophilus':0.118506,(((((GB_GCA_009776625.1:0.0874895,GB_GCA_009777615.1
:0.035934)1.0:0.0437469,(GB_GCA_009778275.1:0.0579781,GB_GCA_009778575.1:0.0629559)0.352
:0.0142351)0.58:0.0130705,GB_GCA_009786295.1:0.0831702)0.562:0.0163012,RS_GCF_000800805.
1:0.0848246)'1.0:g__Methanoplasma':0.0986955,(GB_GCA_002506905.1:0.0887294,GB_GCA_01271
9315.1:0.187605)'1.0:g__UBA328':0.0470595)0.827:0.0222265)0.999:0.0277236,((((GB_GCA_002504
405.1:0.0581924,RS_GCF_009911715.1:0.0463818)0.978:0.0214578,RS_GCF_900767505.1:0.074949
1)1.0:0.0274621,((GB_GCA_002506425.1:0.0357967,GB_GCA_006954465.1:0.036676)1.0:0.0220894
,GB_GCA_002504495.1:0.10607)1.0:0.0670401)'1.0:g__UBA71':0.030359,(GB_GCA_001563305.1:0.0
802806,GB_GCA_002506175.1:0.0673079)'1.0:g__ISO4-
G1':0.0792655)1.0:0.0660357)0.841:0.0211422,((((GB_GCA_002505345.1:0.0130668,GB_GCA_00250
9405.1:0.0128657)0.996:0.00934784,GB_GCA_002498365.1:0.0205125)1.0:0.0291389,GB_GCA_011
620825.1:0.0502127)1.0:0.0662598,GB_GCA_002498605.1:0.15149)'1.0:g__VadinCA11':0.113927)0.
999:0.0285689,GB_GCA_012515075.1:0.155723)1.0:0.0351088,(((((GB_GCA_001421175.1:0.016055
5,GB_GCA_002506255.1:0.021804)0.972:0.0121988,GB_GCA_002502965.1:0.0260071)0.999:0.0268
58,GB_GCA_012522645.1:0.0595771)'1.0:g__RumEn-
M2':0.141456,GB_GCA_012520845.1:0.253076)1.0:0.0545524,GB_GCA_006954405.1:0.179319)0.96
8:0.034778)1.0:0.046205,(((GB_GCA_009776695.1:0.0929949,GB_GCA_009780575.1:0.0998093)0.6
43:0.0157221,(GB_GCA_009780795.1:0.0853407,GB_GCA_009784745.1:0.0956506)0.902:0.0165811
)0.685:0.0220514,GB_GCA_009778265.1:0.0840576)'1.0:g__WRKA01':0.104104)1.0:0.113351,(((((GB
_GCA_003557905.1:0.0303052,GB_GCA_007116915.1:0.0193108)1.0:0.0195386,GB_GCA_00356087
5.1:0.0338884)1.0:0.0490124,GB_GCA_007117455.1:0.077368)1.0:0.0396838,(GB_GCA_003550345.
1:0.035678,GB_GCA_003555025.1:0.0474436)1.0:0.0739025)'1.0:g__PWHV01':0.0575977,GB_GCA_
013329105.1:0.125312)1.0:0.123155)'1.0:f__Methanomethylophilaceae':0.142705,(((((((((GB_GCA_0
01421185.1:0.034082,GB_GCA_002498285.1:0.0302334)0.784:0.0139783,GB_GCA_012518185.1:0.1
38275)0.093:0.0129578,GB_GCA_001512965.1:0.0588375)1.0:0.01929,GB_GCA_002504525.1:0.045
6249)1.0:0.0563971,GB_GCA_012519255.1:0.115007)'1.0:g__DTU008':0.0713063,(((GB_GCA_00206
7635.1:0.0233643,GB_GCA_012719175.1:0.0233287)1.0:0.0772215,GB_GCA_002508545.1:0.070344
8)1.0:0.060586,RS_GCF_000308215.1:0.0989412)'0.731:g__Methanomassiliicoccus':0.028993)0.968:
0.0399312,RS_GCF_000404225.1:0.190064)'1.0:f__Methanomassiliicoccaceae':0.122375,((((((GB_GC
A_002497075.1:0.0178575,GB_GCA_013329565.1:0.0222282)1.0:0.0279945,GB_GCA_012729095.1:
0.0488839)1.0:0.0290193,GB_GCA_002067045.1:0.0548174)1.0:0.0323932,GB_GCA_004525545.1:0.
0791996)'1.0:g__UBA472':0.174662,(GB_GCA_002067865.1:0.125158,GB_GCA_013415865.1:0.1131
51)'1.0:g__MVRC01':0.0914352)1.0:0.0597239,(GB_GCA_003135935.1:0.0269552,GB_GCA_0031538
95.1:0.0295917)'1.0:g__FEN-
33':0.224582)'0.999:f__UBA472':0.0426267)0.97:0.0319979,((GB_GCA_011334735.1:0.0237717,GB_
GCA_014361295.1:0.0193489)'1.0:f__JACIVX01;g__JACIVX01':0.181071,GB_GCA_013415695.1:0.218
814)0.952:0.0344053)1.0:0.0426427)'1.0:o__Methanomassiliicoccales':0.110357,((((GB_GCA_001800

675.1:0.0324608,GB_GCA_001800815.1:0.0238363)'1.0:g__COMBO-56-
21':0.13635,(GB_GCA_011391345.1:0.012458,GB_GCA_013329135.1:0.0131489)'1.0:g__UBA9653':0
.133283)0.866:0.0404935,GB_GCA_013331315.1:0.155559)'1.0:f__UBA10834':0.142818,(GB_GCA_0
11046235.1:0.219847,GB_GCA_011355145.1:0.19404)'1.0:f__DTMW01':0.1646)'1.0:o__UBA10834':
0.0921523)1.0:0.0559363,((((((((((GB_GCA_005878525.1:0.0205248,GB_GCA_005879045.1:0.01384
72)0.992:0.0113851,GB_GCA_005878665.1:0.0354832)0.881:0.00932677,(GB_GCA_005878515.1:0.0
154671,GB_GCA_005878985.1:0.0166627)1.0:0.0203531)1.0:0.0369723,((GB_GCA_005878395.1:0.0
00888829,GB_GCA_005878485.1:0.00135313)1.0:0.0151597,GB_GCA_005878995.1:0.0163685)1.0:0
.0568085)1.0:0.0231147,((((GB_GCA_005879015.1:0.0129826,GB_GCA_005879065.1:0.0143841)1.0:
0.0200093,GB_GCA_005878385.1:0.0398742)1.0:0.0307778,(GB_GCA_005878615.1:0.0203434,GB_
GCA_005878635.1:0.017927)1.0:0.0526316)1.0:0.024665,GB_GCA_005878325.1:0.0887333)0.999:0.
0223781)0.409:0.0169571,GB_GCA_005878415.1:0.0901438)1.0:0.0508806,GB_GCA_013329855.1:0
.132338)0.994:0.0244385,(((GB_GCA_001800745.1:0.127401,GB_GCA_001800825.1:0.113725)0.9:0.
022117,GB_GCA_011362635.1:0.11072)0.989:0.024411,GB_GCA_011331255.1:0.162901)0.134:0.02
24513)'1.0:g__RBG-16-68-
12':0.104688,(GB_GCA_005878915.1:0.105337,GB_GCA_005878955.1:0.0798801)'1.0:g__EA-
19':0.142665)1.0:0.0638041,GB_GCA_005878375.1:0.257032)'1.0:f__RBG-16-68-
12':0.124599,GB_GCA_004377185.1:0.337695)'1.0:o__RBG-16-68-
12':0.0679839,(((GB_GCA_002496385.1:0.0676355,GB_GCA_002499085.1:0.103634)1.0:0.0551088,
GB_GCA_013791785.1:0.120952)'1.0:g__UBA147':0.137942,GB_GCA_001595885.1:0.216879)'1.0:o_
_SG8-5;f__SG8-
5':0.192728)0.994:0.0402373)0.996:0.0451831,(((((GB_GCA_002495185.1:0.0778226,GB_GCA_0025
03985.1:0.0639066)1.0:0.121886,GB_GCA_013290045.1:0.204053)0.601:0.0491122,GB_GCA_01304
4425.1:0.168451)'1.0:f__UBA184;g__UBA184':0.482394,GB_GCA_011363025.1:0.375068)'1.0:o__UB
A184':0.105251,GB_GCA_011333475.1:0.373739)0.927:0.0422578)0.976:0.0392093,(((((GB_GCA_00
3560595.1:0.113306,GB_GCA_007133925.1:0.156547)0.999:0.0354152,GB_GCA_003553085.1:0.110
998)'1.0:g__PWKY01':0.0829632,GB_GCA_003552585.1:0.19188)1.0:0.0642227,GB_GCA_007129655
.1:0.260555)0.998:0.0458091,(((GB_GCA_003551675.1:0.106062,GB_GCA_003554965.1:0.140286)0.
0:0.0222149,GB_GCA_003555395.1:0.0837041)'1.0:g__PWHR01':0.154621,GB_GCA_003551065.1:0.
189193)0.98:0.0412961)'1.0:o__PWKY01;f__PWKY01':0.321775)0.424:0.0292233,((((((((((GB_GCA_0
02204705.1:0.0619992,GB_GCA_011334705.1:0.0618737)'1.0:g__B-
DKE':0.2405,GB_GCA_000965745.1:0.27819)1.0:0.0578027,((GB_GCA_002498845.1:0.225614,GB_G
CA_002503205.1:0.281472)1.0:0.0737455,RS_GCF_001316105.1:0.301082)0.965:0.0427032)0.983:0.
0361823,((GB_GCA_000496135.1:0.253983,RS_GCF_900083515.1:0.335482)1.0:0.116603,(GB_GCA_
002502705.1:0.173849,GB_GCA_011370275.1:0.188309)'1.0:g__UBA582':0.2214)0.797:0.0446283)1
.0:0.0425813,((((RS_GCF_000152265.2:0.00145353,RS_GCF_002078355.1:0.00291237)1.0:0.061729
6,GB_GCA_002505185.1:0.0558609)'1.0:g__Ferroplasma':0.14841,RS_GCF_001402945.1:0.149464)1
.0:0.0969923,RS_GCF_900176435.1:0.22157)1.0:0.145238)0.712:0.0439517,(((RS_GCF_000195915.1
:0.042562,GB_GCA_008709555.1:0.032795)1.0:0.0275421,RS_GCF_003205235.1:0.0425179)1.0:0.08
9752,RS_GCF_000011185.1:0.133994)'1.0:g__Thermoplasma':0.207382)'1.0:f__Thermoplasmatacea
e':0.239453,(GB_GCA_001856825.1:0.170072,GB_GCA_011358095.1:0.152947)'1.0:f__GCA-
001856825;g__GCA-
001856825':0.420369)'1.0:o__Thermoplasmatales':0.102512,(GB_GCA_002878135.1:0.0743164,GB_
GCA_009758405.1:0.0766569)'1.0:o__ARK-15;f__ARK-15;g__ARK-
15':0.336667)0.97:0.0636302,(((RS_GCF_000025665.1:0.0841676,RS_GCF_000327505.1:0.0840654)'
1.0:g__Aciduliprofundum':0.0781613,GB_GCA_013154005.1:0.177108)'1.0:f__Aciduliprofundaceae':
0.131483,GB_GCA_002011395.1:0.31001)'0.999:o__Aciduliprofundales':0.0662599)0.999:0.0533691
,GB_GCA_003649695.1:0.555204)1.0:0.0720562,GB_GCA_003649915.1:0.449421)0.999:0.0541129)'

0.996:c__Thermoplasmata':0.0307206)1.0:0.0358,((((((((((GB_GCA_002496355.1:0.0505736,GB_GC
A_002506745.1:0.0432943)1.0:0.0273996,(GB_GCA_003942085.1:0.0536299,GB_GCA_902384685.1:
0.0684413)0.959:0.0159406)0.877:0.0183384,(GB_GCA_001595945.1:0.0672273,GB_GCA_00290055
5.1:0.04683)1.0:0.0446342)'1.0:g__SM1-
50':0.0913563,GB_GCA_903877165.1:0.215487)1.0:0.075974,GB_GCA_903930505.1:0.195255)1.0:0.
0703502,(((GB_GCA_001595915.1:0.138258,GB_GCA_002900535.1:0.143711)0.964:0.0316451,GB_
GCA_003651045.1:0.117783)1.0:0.0569488,(GB_GCA_002254885.1:0.28087,GB_GCA_003649845.1:
0.172005)1.0:0.072451)0.992:0.0375063)0.834:0.0348351,GB_GCA_003649715.1:0.165011)'1.0:f__
DHVEG-
1':0.0897468,((GB_GCA_003650935.1:0.193188,GB_GCA_003651105.1:0.239802)'1.0:f__B18-
G2':0.0757096,GB_GCA_003649745.1:0.244218)0.0:0.0275485)'1.0:o__DHVEG-
1':0.107015,(((((GB_GCA_002011355.1:0.0944885,GB_GCA_011052825.1:0.127292)0.0:0.0138085,G
B_GCA_003650975.1:0.199488)0.981:0.0340305,((GB_GCA_014361195.1:0.0645663,GB_GCA_01436
1245.1:0.0619335)'1.0:g__JACIWB01':0.153208,GB_GCA_008297865.1:0.107497)1.0:0.0461128)'1.0:
f__JdFR-
43':0.111512,(GB_GCA_008297795.1:0.134396,GB_GCA_011049295.1:0.235673)'1.0:f__DSCA01':0.0
993051)1.0:0.0674095,GB_GCA_002502685.1:0.326288)'1.0:o__UBA202':0.0979346)0.311:0.033335
4,((GB_GCA_013329495.1:0.00355278,GB_GCA_013329505.1:0.00330525)'1.0:f__UBA9212;g__UBA
9212':0.423861,GB_GCA_002838935.1:0.352224)'1.0:o__UBA9212':0.0768278)'1.0:c__E2':0.095199
7,(GB_GCA_003009755.1:0.221346,GB_GCA_003561665.1:0.19673)'1.0:c__SW-10-69-26;o__SW-10-
69-26;f__SW-10-69-
26':0.446993)0.973:0.0346264)0.927:0.0250814,((GB_GCA_002495235.1:0.141979,GB_GCA_011620
765.1:0.121499)'1.0:o__UBA287;f__UBA287;g__UBA287':0.44786,GB_GCA_002494765.1:0.553155)'
1.0:c__UBA186':0.107321)0.986:0.0376505,(GB_GCA_003663565.1:0.0613734,GB_GCA_003663585.
1:0.085739)'1.0:c__B47-G6;o__B47-G6B;f__47-G6;g__B47-
G6':0.56887)'1.0:p__Thermoplasmatota':0.0609196)1.0:0.0602804,((((((((((((((((((((((((((GB_GCA_90
0316895.1:0.01268,GB_GCA_900320515.1:0.0137201)0.98:0.00720467,(GB_GCA_902764015.1:0.02
65187,GB_GCA_902794475.1:0.0211927)0.868:0.0070912)1.0:0.018229,GB_GCA_902788255.1:0.03
47964)0.651:0.0083898,(GB_GCA_902772665.1:0.0214555,GB_GCA_902782595.1:0.00752945)1.0:0.
0523749)0.887:0.00672624,(RS_GCF_003111625.1:0.029637,GB_GCA_902774605.1:0.0359611)0.99
2:0.00902983)0.782:0.00774635,GB_GCA_900313645.1:0.0440445)0.996:0.00993501,GB_GCA_9027
85855.1:0.0635689)0.999:0.0124563,(((RS_GCF_003814835.1:0.0299323,RS_GCF_900769095.1:0.03
12373)1.0:0.0312594,GB_GCA_902770715.1:0.0274457)0.389:0.00961765,RS_GCF_001548675.1:0.0
33214)0.998:0.00979586)0.934:0.00718789,((GB_GCA_900314615.1:0.036481,GB_GCA_902764455.
1:0.0289189)0.888:0.0121059,(GB_GCA_900318035.1:0.0128489,GB_GCA_902771435.1:0.00562137
)1.0:0.0313424)1.0:0.0174703)0.957:0.00750459,((((GB_GCA_900314695.1:0.00704917,GB_GCA_90
0320955.1:0.0143388)1.0:0.0148809,GB_GCA_902801725.1:0.0279156)0.041:0.00590805,GB_GCA_
900319985.1:0.0305391)1.0:0.0157823,GB_GCA_902789505.1:0.0419704)0.991:0.00917305)1.0:0.0
382434,RS_GCF_001639275.1:0.0872965)0.559:0.0185073,(((((RS_GCF_001477655.1:0.0268985,GB_
GCA_902777885.1:0.0313286)0.955:0.00724838,(RS_GCF_900103415.1:0.0477398,GB_GCA_900317
865.1:0.0231276)0.868:0.00768489)0.724:0.00737135,((GB_GCA_900319535.1:0.00165321,GB_GCA
_902768765.1:0.0075478)0.75:0.000316342,GB_GCA_902763935.1:0.000633113)1.0:0.0264918)1.0:
0.0181632,GB_GCA_902796575.1:0.0875842)0.995:0.0198643,GB_GCA_902783085.1:0.0502605)1.0
:0.0380697)1.0:0.0354569,(RS_GCF_000016525.1:0.0101175,RS_GCF_002252585.1:0.0085581)1.0:0.
0696303)0.998:0.0235218,((GB_GCA_002496065.1:0.0373944,RS_GCF_900766745.1:0.0461358)1.0:
0.067499,RS_GCF_003111605.1:0.0732724)0.527:0.0187084)'1.0:g__Methanobrevibacter_A':0.1101
79,((RS_GCF_000320505.1:0.0656226,GB_GCA_900314605.1:0.106862)0.364:0.0256067,(RS_GCF_0
00621965.1:0.0173106,RS_GCF_002208625.1:0.0264905)1.0:0.0633689)'1.0:g__Methanobrevibacter

_B':0.108207)0.965:0.031069,((((((GB_GCA_004553595.1:0.00902679,GB_GCA_902784195.1:0.0065
5032)1.0:0.0139049,GB_GCA_902774685.1:0.0183972)0.994:0.00872597,GB_GCA_902769175.1:0.0
350861)1.0:0.0138885,RS_GCF_900114585.1:0.0467514)1.0:0.0232838,(RS_GCF_000024185.1:0.031
9793,GB_GCA_900321995.1:0.0376378)1.0:0.0214832)1.0:0.0445576,GB_GCA_900314635.1:0.0868
44)'1.0:g__Methanobrevibacter':0.0812477)0.81:0.0248844,GB_GCA_002495685.1:0.151785)1.0:0.0
634137,((((((RS_GCF_000513315.1:0.0111678,RS_GCF_002072215.1:0.0099176)1.0:0.0528252,GB_G
CA_003315655.1:0.077528)1.0:0.0317456,GB_GCA_009784145.1:0.0937734)1.0:0.0305371,RS_GCF_
001639285.1:0.101639)0.884:0.0199879,(GB_GCA_009776495.1:0.0607335,GB_GCA_009777005.1:0
.050277)1.0:0.0780562)'0.994:g__Methanobrevibacter_C':0.0266414,(RS_GCF_001639265.1:0.1316
04,RS_GCF_001639295.1:0.189048)'1.0:g__Methanobrevibacter_D':0.0457912)0.886:0.0248661)1.0:
0.108655,GB_GCA_902795935.1:0.193236)0.998:0.0355237,(((GB_GCA_002509745.1:0.00996963,G
B_GCA_002839705.1:0.0159165)'1.0:g__UBA349':0.0867119,GB_GCA_002504725.1:0.0976153)0.96
9:0.0238901,(GB_GCA_003558085.1:0.131866,GB_GCA_003600755.1:0.0738678)'1.0:g__UBA588':0.
0618184)0.999:0.0327792)0.984:0.0272508,((((((((((GB_GCA_003266105.1:0.0481322,GB_GCA_902
797085.1:0.0431737)0.303:0.0097692,(GB_GCA_013330915.1:0.00305355,GB_GCA_013331465.1:0.
00580513)1.0:0.0378732)0.909:0.01049,GB_GCA_003266165.1:0.0632029)1.0:0.0497232,(((GB_GCA
_003266065.1:0.0382662,RS_GCF_003268005.1:0.0201661)0.204:0.00908896,GB_GCA_003266145.
1:0.0187721)1.0:0.0544106,GB_GCA_003266075.1:0.111609)1.0:0.0373093)1.0:0.0217672,(((GB_GC
A_002509095.1:0.00195732,GB_GCA_900322125.1:0.00181931)1.0:0.0642097,RS_GCF_001729965.
1:0.0648413)0.326:0.0146211,RS_GCF_000012545.1:0.0655761)0.998:0.0166072)0.975:0.0342669,R
S_GCF_003149675.1:0.0998312)'1.0:g__Methanosphaera':0.31933,(((((RS_GCF_000302455.1:0.0256
562,GB_GCA_000309865.1:0.0207043)1.0:0.018745,RS_GCF_002813695.1:0.0400376)0.999:0.01611
3,((GB_GCA_001316325.1:0.0129502,GB_GCA_003491305.1:0.00410637)1.0:0.0119198,RS_GCF_00
0499765.1:0.0366726)1.0:0.0422104)1.0:0.0277223,((GB_GCA_002496805.1:0.0301002,GB_GCA_01
2838205.1:0.0279157)1.0:0.0542103,GB_GCA_002067065.1:0.0511703)1.0:0.0168286)0.514:0.0130
033,GB_GCA_013403005.1:0.0519506)'1.0:g__Methanobacterium':0.104403)0.985:0.0304564,((((((G
B_GCA_003158115.1:0.0117237,GB_GCA_003164415.1:0.0207184)0.994:0.00846945,GB_GCA_0031
51535.1:0.0125969)1.0:0.0303134,(GB_GCA_003162115.1:0.0155732,GB_GCA_003162655.1:0.0099
6246)1.0:0.029926)1.0:0.0198276,RS_GCF_000744455.1:0.0469208)1.0:0.0346974,(RS_GCF_000191
585.1:0.0193225,GB_GCA_009712615.1:0.0143334)1.0:0.0786175)'1.0:g__Methanobacterium_B':0.
0685205,((RS_GCF_000214725.1:0.0570751,GB_GCA_002498385.1:0.0886458)1.0:0.0353947,RS_GC
F_900095295.1:0.100963)'1.0:g__Methanobacterium_C':0.0304862)1.0:0.0304223)0.652:0.016197,(
(((((GB_GCA_002502855.1:0.0179932,GB_GCA_011620845.1:0.0190534)0.8:0.00326356,GB_GCA_00
2067325.1:0.00644579)0.203:0.00311696,GB_GCA_002495625.1:0.00978196)1.0:0.031665,GB_GCA
_002494495.1:0.0831392)1.0:0.0651601,GB_GCA_002067565.1:0.0803586)0.93:0.0239469,(GB_GCA
_003161815.1:0.0418914,GB_GCA_003166335.1:0.0391557)1.0:0.0804573)'1.0:g__Methanobacteriu
m_A':0.0530146)1.0:0.0333316,(((RS_GCF_000745485.1:0.0118288,RS_GCF_002287175.1:0.012959
7)0.986:0.00805265,GB_GCA_002509665.1:0.0250888)1.0:0.0715302,(GB_GCA_002494885.1:0.0413
261,GB_GCA_002505765.1:0.0654086)1.0:0.04518)'1.0:g__Methanobacterium_D':0.0662608)1.0:0.0
385551,GB_GCA_003491285.1:0.142422)1.0:0.0469116)'1.0:f__Methanobacteriaceae':0.0658151,(((
RS_GCF_000008645.1:0.0265742,RS_GCF_000828575.1:0.00491226)1.0:0.0295935,RS_GCF_000145
295.1:0.0354208)1.0:0.0423759,RS_GCF_900095815.1:0.0572148)'1.0:f__Methanothermobacterace
ae;g__Methanothermobacter':0.11682)1.0:0.0502664,(((GB_GCA_001507955.1:0.0418366,RS_GCF_
003264935.1:0.0367692)0.826:0.0103636,GB_GCA_012840175.1:0.0291694)1.0:0.0219141,(GB_GCA
_003584625.1:0.0435341,GB_GCA_011370395.1:0.0380238)1.0:0.0287466)'1.0:f__Methanothermo
bacteraceae_A;g__Methanothermobacter_A':0.108467)1.0:0.0936266,RS_GCF_000166095.1:0.2147
06)'1.0:c__Methanobacteria;o__Methanobacteriales':0.184461,(((((((((RS_GCF_000017225.1:0.0184
93,RS_GCF_000018485.1:0.0123941)0.901:0.0057994,RS_GCF_000016125.1:0.0142453)1.0:0.01355

63,RS_GCF_002945325.1:0.0232536)1.0:0.0615523,RS_GCF_000017165.1:0.0924954)1.0:0.0691369,
RS_GCF_000006175.1:0.211083)'1.0:g__Methanococcus':0.0813368,RS_GCF_000376965.1:0.090194
7)1.0:0.03866,((RS_GCF_000017185.1:0.183032,RS_GCF_000179575.2:0.0694153)'1.0:g__Methanot
hermococcus_A':0.0394297,(RS_GCF_003351865.1:0.0312264,RS_GCF_004310395.1:0.0509472)'1.0:
g__Methanofervidicoccus':0.139697)0.766:0.0401944)1.0:0.0895087,(RS_GCF_000214415.1:0.02561
62,RS_GCF_000243455.1:0.0444666)'1.0:g__Methanotorris':0.0666945)'1.0:f__Methanococcaceae':
0.0599481,(((((RS_GCF_000023985.1:0.0355312,RS_GCF_000739065.1:0.017688)0.979:0.00891596,
RS_GCF_000025525.1:0.0152496)0.985:0.00848393,RS_GCF_000091665.1:0.0163798)1.0:0.0182784
,(RS_GCF_000024625.1:0.0114987,GB_GCA_013153845.1:0.00805832)1.0:0.058603)'1.0:g__Methan
ocaldococcus':0.0360089,(RS_GCF_000092305.1:0.139736,RS_GCF_000371805.1:0.110648)'1.0:g__
Methanocaldococcus_A':0.0500328)'1.0:f__Methanocaldococcaceae':0.093699)'1.0:c__Methanococ
ci;o__Methanococcales':0.296268)0.881:0.043631,((RS_GCF_000007185.1:0.00990988,RS_GCF_002
201895.1:0.0271506)1.0:0.113121,GB_GCA_013154335.1:0.14461)'1.0:c__Methanopyri;o__Methan
opyrales;f__Methanopyraceae;g__Methanopyrus':0.392787)0.997:0.0336377,(((((((((((((RS_GCF_00
0221185.1:0.00830723,RS_GCF_012027645.1:0.00818696)0.99:0.00371727,(RS_GCF_001484195.1:0
.00942483,RS_GCF_012027495.1:0.0127662)1.0:0.00568023)0.135:0.00110162,RS_GCF_006274605.
1:0.0122897)0.994:0.00651074,RS_GCF_904067545.1:0.0103266)1.0:0.0181985,GB_GCA_01105017
5.1:0.0352533)0.987:0.00879049,(((((RS_GCF_002214565.1:0.0124789,RS_GCF_012027615.1:0.0187
224)0.942:0.00549725,RS_GCF_002214465.1:0.0260374)0.891:0.00592621,RS_GCF_012027305.1:0.
0129629)0.412:0.00754734,RS_GCF_002214545.1:0.0222216)0.996:0.00756021,(RS_GCF_00221436
5.1:0.0404855,RS_GCF_002214525.1:0.086895)1.0:0.0270048)0.999:0.00959042)0.42:0.00775387,R
S_GCF_000265525.1:0.0345096)0.837:0.00743731,(RS_GCF_002214485.1:0.0360678,RS_GCF_00221
4505.1:0.0319177)0.974:0.0111525)0.96:0.00639168,(((RS_GCF_000018365.1:0.0126688,RS_GCF_0
12027395.1:0.00656623)1.0:0.0173022,(RS_GCF_001647085.1:0.0132309,RS_GCF_012027555.1:0.0
0511042)1.0:0.0234046)1.0:0.0267519,RS_GCF_012027355.1:0.0360928)0.267:0.00675983)1.0:0.02
55309,((((RS_GCF_000258515.1:0.056245,RS_GCF_002214385.1:0.0165161)1.0:0.0301353,(RS_GCF_
002214585.1:0.0363092,RS_GCF_012027595.1:0.0708181)1.0:0.0237889)0.94:0.00982458,((RS_GCF
_000022365.1:0.0145098,RS_GCF_000151205.2:0.0150601)1.0:0.0165035,RS_GCF_000816105.1:0.0
265595)1.0:0.0233232)0.303:0.0133398,((((RS_GCF_012027635.1:0.00919574,RS_GCF_900198835.1
:0.0116818)0.531:0.00327941,RS_GCF_000585495.1:0.00730831)1.0:0.0208824,RS_GCF_000769655
.1:0.0275812)1.0:0.0202081,(RS_GCF_000009965.1:0.0120216,RS_GCF_001592435.1:0.013319)1.0:0
.0384705)1.0:0.0123777)0.991:0.0138925)0.996:0.0206585,GB_GCA_013153595.1:0.164388)'1.0:g_
_Thermococcus':0.0491936,((((((GB_GCA_003663525.1:0.0102939,GB_GCA_003663535.1:0.0087356
6)0.999:0.0103577,RS_GCF_000022545.1:0.0192117)1.0:0.0403968,(RS_GCF_001317345.1:0.004663
53,RS_GCF_012027425.1:0.0113334)1.0:0.0378161)1.0:0.0349388,((RS_GCF_000246985.2:0.008935
34,RS_GCF_001484685.1:0.0239476)1.0:0.0137336,RS_GCF_000430485.1:0.0220017)1.0:0.0174083)
'1.0:g__Thermococcus_A':0.0549511,((((GB_GCA_003660495.1:0.00184835,GB_GCA_003663695.1:0
.00134854)1.0:0.0710233,RS_GCF_000725425.1:0.0454562)1.0:0.0282055,RS_GCF_000966265.1:0.1
10447)'1.0:g__Palaeococcus':0.0451813,GB_GCA_002009975.1:0.0834744)1.0:0.0269001)0.997:0.01
6869,((RS_GCF_000517445.1:0.0172437,RS_GCF_012027325.1:0.0109839)0.985:0.00869599,RS_GCF
_000151105.2:0.018299)'1.0:g__Thermococcus_B':0.0385264)1.0:0.0338228)0.998:0.0273403,((((((R
S_GCF_001577775.1:0.0126037,GB_GCA_003660485.1:0.0188948)0.079:0.00486338,RS_GCF_00221
4605.1:0.0147945)1.0:0.0201971,RS_GCF_000263735.1:0.0384433)1.0:0.0158457,((RS_GCF_000011
105.1:0.0422647,RS_GCF_000195935.2:0.0323201)0.905:0.0128684,RS_GCF_000211475.1:0.045239
4)1.0:0.0223444)0.773:0.0137426,RS_GCF_000007305.1:0.0536943)1.0:0.0402347,RS_GCF_0002159
95.1:0.0573436)'1.0:g__Pyrococcus':0.0345102)'1.0:o__Thermococcales;f__Thermococcaceae':0.286
289,((((((GB_GCA_001587575.1:0.0281999,GB_GCA_013178285.1:0.0313441)0.99:0.00946324,(GB_
GCA_001587595.1:0.0137467,GB_GCA_012799835.1:0.0279355)1.0:0.0570421)0.977:0.0094491,(GB

_GCA_001587715.1:0.0280515,GB_GCA_013329085.1:0.0239914)1.0:0.0189635)0.999:0.0209453,G
B_GCA_003558335.1:0.10495)0.941:0.0319851,GB_GCA_001587675.1:0.0676167)'1.0:f__Methanof
astidiosaceae;g__Methanofastidiosum':0.377439,((GB_GCA_004212155.1:0.0100937,GB_GCA_0113
72835.1:0.0130212)'1.0:f__NM3;g__NM3':0.419977,GB_GCA_003660605.1:0.349994)1.0:0.0728178
)'1.0:o__Methanofastidiosales':0.13995)'1.0:c__Thermococci':0.0723644)'1.0:p__Methanobacteriota
':0.0402517,(((((((GB_GCA_001515185.1:0.0455806,GB_GCA_004375695.1:0.054859)'1.0:g__DG-
33':0.147701,(GB_GCA_003661465.1:0.118064,GB_GCA_012962055.1:0.116775)'1.0:g__B75-
G9':0.0805278)1.0:0.0484168,(GB_GCA_001515205.2:0.0652632,GB_GCA_014361095.1:0.0850703)'
1.0:g__Hadarchaeum':0.147866)'1.0:f__Hadarchaeaceae':0.0986771,(GB_GCA_011367225.1:0.1358
69,GB_GCA_011370445.1:0.1314)'1.0:f__DTKF01;g__DTKF01':0.223425)0.992:0.0540242,((GB_GCA_
004347925.1:0.108194,GB_GCA_011367165.1:0.0899325)'1.0:f__WYZ-LMO6;g__WYZ-
LMO6':0.216276,(GB_GCA_011361855.1:0.155803,GB_GCA_011367145.1:0.153627)'1.0:f__DSZJ01;g
__DSZJ01':0.223425)0.998:0.0654397)'1.0:c__Hadarchaeia;o__Hadarchaeales':0.18804,GB_GCA_003
660555.1:0.471163)'0.995:p__Hadarchaeota':0.0535711,((((GB_GCA_002897955.1:0.0157313,GB_G
CA_003229935.1:0.0353801)'1.0:g__BMS3B':0.290446,(GB_GCA_013152685.1:0.0980608,GB_GCA_
013152995.1:0.213798)1.0:0.121363)'1.0:f__BMS3B':0.0867352,(GB_GCA_002011125.1:0.256728,G
B_GCA_013152845.1:0.299709)0.982:0.0445224)0.506:0.0384486,((GB_GCA_002923215.1:0.049409
4,GB_GCA_003230355.1:0.0432555)'1.0:g__SZUA-
236':0.197436,GB_GCA_003695745.1:0.166259)'1.0:f__SZUA-
236':0.252972)'1.0:p__Hydrothermarchaeota;c__Hydrothermarchaeia;o__Hydrothermarchaeales':0.
161991)0.996:0.0358465)0.953:0.0252543)1.0:0.0369752,((((((((((((((((((((((((((((((((((GB_GCA_0037
02525.1:0.0083154,GB_GCA_003702545.1:0.0154679)0.971:0.00525321,GB_GCA_003702495.1:0.01
63035)0.995:0.00542723,RS_GCF_000018465.1:0.0155486)1.0:0.00785669,((GB_GCA_000746765.1:
0.00514086,GB_GCA_000746785.1:0.00294474)0.974:0.00527213,GB_GCA_000746685.1:0.0088608
4)1.0:0.0109666)0.974:0.0068059,RS_GCF_006740685.1:0.0158641)0.985:0.00770268,(RS_GCF_000
299365.1:0.027079,RS_GCF_000875775.1:0.0271656)0.295:0.00641557)1.0:0.0265966,GB_GCA_008
080815.1:0.127857)0.974:0.0148286,((((((GB_GCA_001437625.1:0.0167888,GB_GCA_014384445.1:0
.0157452)1.0:0.0393759,GB_GCA_902514095.1:0.0293546)0.923:0.012579,GB_GCA_002737455.1:0.
059286)0.999:0.0160737,GB_GCA_902514275.1:0.0246249)0.999:0.0178994,((RS_GCF_001541925.1
:0.0132727,GB_GCA_003702465.1:0.023068)1.0:0.0133946,(RS_GCF_002156965.1:0.0104681,GB_G
CA_003331425.1:0.0124875)0.995:0.00705576)1.0:0.019981)1.0:0.0512885,GB_GCA_013867245.1:0
.0347984)1.0:0.0360666)1.0:0.0226932,((((((((GB_GCA_013203245.1:0.0500045,RS_GCF_013407165
.1:0.0284919)1.0:0.0156229,GB_GCA_014384555.1:0.0392597)0.992:0.00966751,RS_GCF_01340714
5.1:0.0327717)0.932:0.00470021,(RS_GCF_000299395.1:0.0302137,GB_GCA_013043295.1:0.038247
3)0.623:0.00602574)0.999:0.0150384,(GB_GCA_002506665.1:0.137432,GB_GCA_014075315.1:0.067
7206)0.656:0.0115369)0.997:0.0118381,GB_GCA_002788515.1:0.06187)0.818:0.0110682,RS_GCF_0
00242875.2:0.0618252)0.992:0.0112823,(((RS_GCF_000956175.1:0.0126418,RS_GCF_014078525.1:0
.0191989)1.0:0.0190895,RS_GCF_013407185.1:0.0217754)0.949:0.007548,RS_GCF_003175215.1:0.0
381831)1.0:0.0344803)0.989:0.0100168)0.806:0.0100059,(((((GB_GCA_000484975.1:0.0195205,RS_
GCF_013390905.1:0.016717)1.0:0.045706,GB_GCA_013911135.1:0.0444339)0.98:0.0120813,GB_GC
A_001510275.1:0.048717)1.0:0.0250464,(((GB_GCA_001543015.1:0.00884679,GB_GCA_002317795.
1:0.00699392)1.0:0.0585521,GB_GCA_002730325.1:0.0496369)0.987:0.0227212,GB_GCA_01321528
5.1:0.0738479)0.933:0.0148478)0.982:0.0102771,GB_GCA_013002265.1:0.0870723)0.832:0.009386
49)1.0:0.0223824,((((GB_GCA_003724285.1:0.0483155,GB_GCA_003724325.1:0.0598124)1.0:0.0439
957,RS_GCF_900620265.1:0.292817)1.0:0.0830085,GB_GCA_007571225.1:0.27839)1.0:0.055377,GB
_GCA_007571135.1:0.109977)0.999:0.0310365)'1.0:g__Nitrosopumilus':0.0325283,((((((GB_GCA_00
4297665.1:0.0185225,GB_GCA_013140415.1:0.0122312)0.773:0.00624128,GB_GCA_009697485.1:0.
0167103)0.997:0.0193379,(GB_GCA_003569705.1:0.0300343,GB_GCA_005798405.1:0.0298227)0.32

6:0.00470684)0.993:0.0113529,((RS_GCF_000220175.1:0.0238988,GB_GCA_012030815.1:0.0259054)1.0:0.0289419,GB_GCA_000204585.1:0.0458515)1.0:0.0160291)0.467:0.00988219,GB_GCA_002737445.1:0.0699802)'1.0:g__Nitrosarchaeum':0.0449586,GB_GCA_002781805.1:0.0998756)0.354:0.0128422)0.942:0.0181589,(GB_GCA_011773785.1:0.0620117,GB_GCA_013867335.1:0.0543711)'1.0:g__JACEMX01':0.0471639)1.0:0.0655236,(((GB_GCA_003724275.1:0.0386709,GB_GCA_007570915.1:0.0377232)1.0:0.327345,GB_GCA_000200715.1:0.200404)'0.991:g__Cenarchaeum':0.0688546,(GB_GCA_009840065.1:0.0836323,GB_GCA_009843815.1:0.0774284)'1.0:g__VYCS01':0.338208)1.0:0.0797856)0.294:0.0197615,(GB_GCA_003352285.1:0.00540256,RS_GCF_014078545.1:0.00505164)'1.0:g__PXYB01':0.125572)0.974:0.0246005,GB_GCA_011055585.1:0.157548)0.871:0.0181282,GB_GCA_011523285.1:0.400763)0.273:0.0124264,((((((GB_GCA_000402075.1:0.0279633,RS_GCF_013390745.1:0.0120199)1.0:0.0304399,(RS_GCF_013390375.1:0.0169037,RS_GCF_013390765.1:0.0390922)1.0:0.0322591)0.893:0.0112278,RS_GCF_013390785.1:0.0620773)0.873:0.0125062,(((GB_GCA_002709285.1:0.0093394,GB_GCA_902578515.1:0.0110546)0.993:0.0177422,GB_GCA_902590655.1:0.0185652)1.0:0.0189293,GB_GCA_902606965.1:0.0368658)1.0:0.0317063)1.0:0.0534652,(GB_GCA_000746705.1:0.0684702,GB_GCA_012964965.1:0.0546152)1.0:0.0490453)1.0:0.122709,(((((((RS_GCF_000812185.1:0.0231795,GB_GCA_902552015.1:0.0127885)0.74:0.0055242,GB_GCA_902511735.1:0.032854)0.975:0.00986891,GB_GCA_001510295.1:0.0512942)0.871:0.0120679,GB_GCA_902512865.1:0.0522898)0.842:0.00984347,(GB_GCA_001627235.1:0.00908214,GB_GCA_902566725.1:0.0210311)1.0:0.0263656)1.0:0.10707,(GB_GCA_002698885.1:0.103939,GB_GCA_902606945.1:0.107324)0.774:0.0234022)1.0:0.0675468,GB_GCA_902608175.1:0.163525)1.0:0.0618721)'1.0:g__Nitrosopelagicus':0.0475567)0.206:0.0191896,(GB_GCA_001443365.1:0.0710521,GB_GCA_008974855.1:0.0846007)'1.0:g__CSP1-
1':0.0745227)1.0:0.0406739,((((((GB_GCA_002499525.1:0.0666918,GB_GCA_010028495.1:0.0390181)0.983:0.0115567,(RS_GCF_002787055.1:0.0252772,GB_GCA_900299125.1:0.067474)0.997:0.0117411)1.0:0.023532,RS_GCF_000955905.1:0.0447738)1.0:0.0602582,GB_GCA_005240025.1:0.116396)0.692:0.0184358,RS_GCF_000723185.1:0.0970749)0.991:0.0263725,((RS_GCF_000685395.1:0.00646108,RS_GCF_013407275.1:0.00896132)1.0:0.0262126,GB_GCA_004322465.1:0.0326366)1.0:0.0805133)'1.0:g__Nitrosotenuis':0.0947124)1.0:0.0710229,((((((((RS_GCF_900143675.1:0.0661891,RS_GCF_900177045.1:0.0348989)0.864:0.00877491,RS_GCF_011319475.1:0.0255475)0.377:0.00559115,(GB_GCA_013288545.1:0.0199984,RS_GCF_900065925.1:0.0223124)0.963:0.0117316)0.998:0.0187354,(GB_GCA_007280465.1:0.0192775,RS_GCF_900167955.1:0.0165879)1.0:0.028837)1.0:0.0738419,RS_GCF_011319465.1:0.0983616)0.861:0.0199136,((GB_GCA_001920395.1:0.0588663,GB_GCA_005877205.1:0.0408919)0.8:0.0141692,GB_GCA_001917995.1:0.0575651)0.999:0.0344563)'1.0:g__Nitrosotalea':0.0556983,((GB_GCA_007280335.1:0.0175708,GB_GCA_013287585.1:0.0203211)1.0:0.0250779,GB_GCA_005877305.1:0.0371385)'1.0:g__TA-
20':0.0528984)0.999:0.0407964,GB_GCA_013330055.1:0.0867793)1.0:0.0690687)'1.0:f__Nitrosopumilaceae':0.229858,(((((((GB_GCA_000802205.2:0.030896,GB_GCA_009379865.1:0.0215634)0.962:0.0148942,(RS_GCF_007826885.1:0.0136467,GB_GCA_013694585.1:0.00839608)1.0:0.0197242)1.0:0.0781819,(((RS_GCF_001870125.1:0.0353649,GB_GCA_013114705.1:0.0412951)1.0:0.0335077,RS_GCF_900696045.1:0.0726707)1.0:0.039215,RS_GCF_008389435.1:0.123394)0.99:0.0370464)1.0:0.0695472,GB_GCA_013821245.1:0.144677)'1.0:g__Nitrosocosmicus':0.201581,((GB_GCA_014523485.1:0.0930411,GB_GCA_014523515.1:0.0720661)'1.0:g__TH1177':0.160556,GB_GCA_013114725.1:0.217732)1.0:0.0693467)0.895:0.0413935,(((GB_GCA_005877075.1:0.0604911,GB_GCA_014523495.1:0.0375637)0.999:0.0249574,(GB_GCA_005877345.1:0.0115442,GB_GCA_014523525.1:0.0068566)1.0:0.044946)0.0:0.0202825,GB_GCA_014523595.1:0.051754)'1.0:g__TA-
21':0.270486)0.913:0.0251898,((((((RS_GCF_000698785.1:0.0188944,RS_GCF_000730285.1:0.0265924)1.0:0.0585034,GB_GCA_013388945.1:0.0919001)1.0:0.0415678,GB_GCA_013114715.1:0.12866)1.0:0.0451418,((RS_GCF_000303155.1:0.0348905,GB_GCA_002501845.1:0.0622573)1.0:0.045495,GB

_GCA_002494895.1:0.127202)0.996:0.0354225)'1.0:g__Nitrososphaera':0.0959413,((((GB_GCA_002
501855.1:0.00509136,GB_GCA_003176995.1:0.013597)0.987:0.00869552,GB_GCA_009665115.1:0.0
0785405)1.0:0.0796717,RS_GCF_009898475.1:0.164148)'1.0:g__UBA10452':0.127473,GB_GCA_014
523685.1:0.240814)0.828:0.0347478)0.742:0.0331392,(((GB_GCA_009377575.1:0.0158331,GB_GCA
_014523695.1:0.0180843)1.0:0.0398374,GB_GCA_014523665.1:0.0412681)0.62:0.0175064,GB_GCA
_014523625.1:0.0434577)'1.0:g__TH5896':0.265911)0.994:0.0355535)'1.0:f__Nitrososphaeraceae':0
.1325)1.0:0.0579688,(((((GB_GCA_002898655.1:0.025342,RS_GCF_900248165.1:0.00969056)1.0:0.0
217104,GB_GCA_013538805.1:0.0295321)1.0:0.0356328,GB_GCA_013538675.1:0.0581357)'1.0:g__
Nitrosocaldus':0.246359,GB_GCA_013538715.1:0.290594)'1.0:f__Nitrosocaldaceae':0.0669904,(GB_
GCA_002713325.1:0.158282,GB_GCA_011331095.1:0.11017)'1.0:f__UBA213;g__UBA213':0.141408)
0.968:0.0480969)1.0:0.145279,((((GB_GCA_013306035.1:0.0231387,GB_GCA_013378385.1:0.01477
89)1.0:0.0530677,GB_GCA_002495905.1:0.0645702)1.0:0.113944,GB_GCA_002693165.1:0.133328)1
.0:0.0969093,GB_GCA_002713205.1:0.208495)'1.0:f__UBA57;g__UBA57':0.345346)0.999:0.0521806,
(GB_GCA_011355215.1:0.0426388,GB_GCA_011605775.1:0.0378612)'1.0:f__JAAOZN01;g__JAAOZN
01':0.309247)0.081:0.0293964,((((((GB_GCA_005877225.1:0.0183828,GB_GCA_005877365.1:0.0163
631)0.996:0.0172039,GB_GCA_005877185.1:0.024782)1.0:0.176961,GB_GCA_002495205.1:0.26414
9)'0.923:g__UBA183':0.048159,(GB_GCA_003135575.1:0.211347,GB_GCA_003164815.1:0.186634)'1
.0:g__BOG-
1369':0.152962)0.978:0.077928,RS_GCF_902812495.1:0.255945)'1.0:f__UBA183':0.233713,((GB_GC
A_013288145.1:0.228574,RS_GCF_902812485.1:0.22235)'1.0:f__CADDZS01':0.185851,GB_GCA_002
495315.1:0.412838)0.935:0.0475076)0.967:0.0475311)0.999:0.0471878,(GB_GCA_011358735.1:0.10
9158,GB_GCA_013388925.1:0.156912)'1.0:f__JACAEJ01;g__JACAEJ01':0.231253)'0.999:o__Nitrososp
haerales':0.0616329,GB_GCA_014361085.1:0.460899)0.808:0.0436537,((((GB_GCA_002506605.1:0.0
854576,GB_GCA_009903795.1:0.0773889)1.0:0.191838,GB_GCA_002499005.1:0.279345)'1.0:f__UB
A164;g__UBA164':0.338618,(GB_GCA_011054345.1:0.139087,RS_GCF_013340765.1:0.133589)'1.0:f
__Conexivisphaeraceae;g__Conexivisphaera':0.351534)0.287:0.0572017,GB_GCA_011333845.1:0.44
6674)'1.0:o__Conexivisphaerales':0.110951)1.0:0.126794,(((GB_GCA_003661505.1:0.000952199,GB_
GCA_003661525.1:0.000256481)'1.0:g__B109-
G9':0.296882,GB_GCA_002011085.1:0.24241)1.0:0.111584,GB_GCA_002011075.1:0.336816)'1.0:o_
_Geothermarchaeales;f__JDFR-
13':0.362938)1.0:0.0628572,((((((((GB_GCA_000405685.1:0.000487921,GB_GCA_011337925.1:0.001
95401)1.0:0.0571304,GB_GCA_011375845.1:0.0481784)1.0:0.0410828,GB_GCA_011363825.1:0.088
8522)1.0:0.0923965,GB_GCA_011364615.1:0.153255)'1.0:g__JGI-0000106-
J15':0.0975944,(GB_GCA_002255105.1:0.163718,GB_GCA_003660635.1:0.173979)'1.0:g__EX4484-
121':0.0705562)1.0:0.116201,((GB_GCA_011363045.1:0.0505903,GB_GCA_011364235.1:0.0488533)
1.0:0.03635,GB_GCA_003056285.1:0.0713386)'1.0:g__NZ13-MG1':0.243952)'1.0:f__NZ13-
MGT':0.0566753,(((GB_GCA_000270325.1:0.0211404,GB_GCA_011364605.1:0.0199722)1.0:0.16967
1,(GB_GCA_011373345.1:0.171984,GB_GCA_011373645.1:0.141599)0.999:0.0489216)'1.0:g__Caldar
chaeum':0.133271,GB_GCA_002898355.1:0.235042)'1.0:f__Caldarchaeaceae':0.185799)1.0:0.05619
96,((((((GB_GCA_002898395.1:0.0603241,GB_GCA_009889515.1:0.0337531)1.0:0.0525617,GB_GCA
_011373505.1:0.105167)1.0:0.047505,GB_GCA_011373385.1:0.14195)'1.0:g__HRBIN02':0.0896492,
GB_GCA_000494185.1:0.210947)1.0:0.142566,(GB_GCA_000494145.1:0.072284,GB_GCA_01136426
5.1:0.0666292)'1.0:g__JGI-OTU-
1':0.294786)0.991:0.0505767,(GB_GCA_011334935.1:0.0663058,GB_GCA_011362255.1:0.0860322)'
1.0:g__DTDI01':0.185768)'1.0:f__HR02':0.176283)'1.0:o__Caldarchaeales':0.220332)'0.99:c__Nitroso
sphaeria':0.03715,((((GB_GCA_011362045.1:0.0593061,GB_GCA_011363915.1:0.0830888)1.0:0.0503
288,(GB_GCA_011389375.1:0.111381,GB_GCA_011605725.1:0.118874)0.809:0.0334564)'1.0:f__JAA
OZP01;g__JAAOZP01':0.378288,(GB_GCA_011371845.1:0.440593,GB_GCA_011605715.1:0.466417)0

.998:0.109638)1.0:0.106911,(GB_GCA_002255045.1:0.403548,GB_GCA_011332645.1:0.423035)0.99
5:0.0886824)'1.0:c__EX4484-205;o__EX4484-
205':0.18901)1.0:0.0468515,(((((((((((((((((((((((((((((GB_GCA_012511995.1:0.0966937,GB_GCA_01251
5445.1:0.078465)1.0:0.0290316,GB_GCA_011367965.1:0.0499604)0.91:0.018745,GB_GCA_0113326
15.1:0.0554987)0.921:0.0194511,(GB_GCA_009781675.1:0.0439324,GB_GCA_009783705.1:0.04163
2)1.0:0.0758223)1.0:0.0334865,GB_GCA_012513715.1:0.107207)1.0:0.0392573,((GB_GCA_0031517
35.1:0.0448588,GB_GCA_903901015.1:0.0671003)0.995:0.0175018,GB_GCA_003141855.1:0.029615
)1.0:0.0396314)1.0:0.0256561,(GB_GCA_003164295.1:0.0810832,GB_GCA_003165195.1:0.0874966)
0.995:0.0206189)0.073:0.0156809,GB_GCA_003133625.1:0.0536568)1.0:0.0244828,RS_GCF_003589
585.1:0.0902868)0.906:0.0240431,((((GB_GCA_009781395.1:0.0463378,GB_GCA_009787585.1:0.05
60716)0.93:0.0127524,GB_GCA_009779045.1:0.0544116)1.0:0.0154258,GB_GCA_009776805.1:0.08
4395)1.0:0.0184893,(GB_GCA_009778385.1:0.0416656,GB_GCA_009787175.1:0.0890331)0.739:0.01
12126)1.0:0.111145)'1.0:g__PALSA-
986':0.0777667,(GB_GCA_001273385.1:0.255056,GB_GCA_013330975.1:0.173949)0.823:0.031863)0
.741:0.0213349,(GB_GCA_001273335.1:0.0737781,GB_GCA_007050895.1:0.0995955)'1.0:g__SG8-
32-
3':0.0701529)0.443:0.0220681,GB_GCA_011051215.1:0.102633)1.0:0.0394537,GB_GCA_011334665.
1:0.204761)1.0:0.0920937,((GB_GCA_002779555.1:0.127695,GB_GCA_011370995.1:0.201071)1.0:0.
0375354,(GB_GCA_013388895.1:0.0883997,GB_GCA_013388905.1:0.0718918)'1.0:g__JACAEI01':0.0
715861)0.979:0.0207238)1.0:0.0250681,((GB_GCA_002010975.1:0.0833413,GB_GCA_003601605.1:
0.0462168)'1.0:g__JdFR-
07':0.0540451,GB_GCA_011053435.1:0.111581)0.0:0.0125392)1.0:0.0197534,GB_GCA_004376975.1
:0.151707)0.592:0.0136025,((((GB_GCA_011373445.1:0.0322439,GB_GCA_011373895.1:0.0349822)'
1.0:g__DRVV01':0.0974922,GB_GCA_011364805.1:0.155943)0.866:0.0253776,(GB_GCA_011364585.
1:0.0566161,GB_GCA_011373965.1:0.058431)'1.0:g__DRVP01':0.0491896)0.792:0.0199068,GB_GCA
_009889605.1:0.0965113)1.0:0.0547789)0.987:0.0178191,((GB_GCA_003601785.1:0.00186558,GB_
GCA_003662325.1:0.00190011)'1.0:g__PIYA01':0.0685252,GB_GCA_003601875.1:0.116533)1.0:0.03
11402)1.0:0.072213,((GB_GCA_002010925.1:0.0782916,GB_GCA_002507245.1:0.0655818)1.0:0.035
167,GB_GCA_003661945.1:0.0841495)'1.0:g__UBA233':0.121596)'1.0:f__UBA233':0.0476346,GB_GC
A_004376645.1:0.309395)0.0:0.0244902,((((GB_GCA_003096235.1:0.0766008,GB_GCA_003096255.
1:0.103795)0.892:0.0197735,GB_GCA_004376385.1:0.0551969)'1.0:g__BIN-L-
1':0.16289,GB_GCA_001399795.1:0.119794)1.0:0.0449405,((GB_GCA_003662715.1:0.165015,GB_GC
A_004376295.1:0.139354)0.881:0.0224734,GB_GCA_001399805.1:0.182855)0.622:0.0187516)'1.0:f_
_BA1':0.0598938)0.998:0.0364567,GB_GCA_009889585.1:0.293949)1.0:0.0488788,((((((GB_GCA_00
1593845.1:0.114142,GB_GCA_003661975.1:0.115577)0.0:0.0251115,GB_GCA_003601725.1:0.07211
97)0.778:0.0215148,GB_GCA_003601695.1:0.135293)'1.0:g__B63':0.0771637,GB_GCA_003601835.1
:0.161363)1.0:0.0671579,(((GB_GCA_011363945.1:0.169732,GB_GCA_011364715.1:0.0923717)0.99
9:0.0302996,(GB_GCA_011366945.1:0.0788663,GB_GCA_011380195.1:0.0704459)0.999:0.0289511)
1.0:0.0332663,GB_GCA_011363705.1:0.116551)'1.0:g__DTEX01':0.123874)1.0:0.0538189,((GB_GCA
_001593925.1:0.0587363,GB_GCA_001593935.1:0.0638539)'1.0:g__B26-
1':0.118899,GB_GCA_003662645.1:0.135797)1.0:0.0465152)'1.0:f__B26-
1':0.0780048)0.985:0.0389526,GB_GCA_002490245.1:0.322299)'1.0:o__B26-
1':0.047512,((GB_GCA_002255025.1:0.112025,GB_GCA_011040545.1:0.114473)'1.0:f__EX4484-
135;g__EX4484-135':0.195939,GB_GCA_003662535.1:0.218723)'1.0:o__EX4484-
135':0.0798287)1.0:0.0520855,(((((((GB_GCA_001775965.1:0.00240138,GB_GCA_001776015.1:0.006
26261)1.0:0.155139,GB_GCA_013619005.1:0.200306)'1.0:g__RBG-16-57-
9':0.111328,(GB_GCA_001774245.1:0.057826,GB_GCA_013329615.1:0.0823944)'1.0:g__UBA8941':0
.212031)1.0:0.0842209,(GB_GCA_002726865.1:0.119425,GB_GCA_014381835.1:0.0621312)'1.0:g__

GCA-
2726865':0.188736)1.0:0.0587161,GB_GCA_003662205.1:0.118823)1.0:0.0735578,GB_GCA_001593
875.1:0.219614)1.0:0.0935187,GB_GCA_003601775.1:0.212347)'1.0:o__TCS64;f__TCS64':0.230667)
0.993:0.0484513,((((((((((((((GB_GCA_005883075.1:0.0174591,GB_GCA_005884605.1:0.021721)0.21
7:0.00443327,GB_GCA_005883085.1:0.0162465)0.983:0.00699366,GB_GCA_005888745.1:0.031093
5)0.951:0.00526949,GB_GCA_001918745.1:0.0299052)0.999:0.0122445,((GB_GCA_005882895.1:0.0
144386,GB_GCA_005883695.1:0.0277692)0.384:0.00356074,GB_GCA_005881695.1:0.0182136)1.0:0
.0143858)0.123:0.00876431,(((GB_GCA_005884195.1:0.0182962,GB_GCA_005888755.1:0.0160092)
1.0:0.0114656,GB_GCA_005881615.1:0.0241044)1.0:0.0135287,GB_GCA_001918765.1:0.029916)0.9
81:0.00824193)1.0:0.119589,(((GB_GCA_001915065.1:0.0219371,GB_GCA_005882905.1:0.0217953)
1.0:0.0530375,GB_GCA_002495485.1:0.0900813)0.999:0.0318931,((GB_GCA_005882955.1:0.014095
,GB_GCA_005888635.1:0.0228373)0.994:0.0140476,GB_GCA_001919285.1:0.0265146)1.0:0.068726
9)1.0:0.0910876)'0.997:g__40CM-2-53-
6':0.0416122,GB_GCA_005888735.1:0.190152)1.0:0.0592794,(GB_GCA_005888675.1:0.00557501,G
B_GCA_005888695.1:0.00677885)'1.0:g__BA-13':0.198005)'1.0:f__40CM-2-53-
6':0.233505,(GB_GCA_001768965.1:0.0891958,GB_GCA_003152955.1:0.0922728)'1.0:f__FEN-
987;g__FEN-
987':0.270177)1.0:0.08428,((GB_GCA_011338225.1:0.0627634,GB_GCA_011369655.1:0.0407854)'1.
0:f__DTDX01;g__DTDX01':0.230058,(GB_GCA_011358085.1:0.297077,GB_GCA_011389385.1:0.2771
55)'1.0:f__DTGE01':0.0788663)1.0:0.0691688)0.477:0.0347221,((GB_GCA_001775955.1:0.0348179,
GB_GCA_007050885.1:0.0571737)'1.0:g__RBG-13-38-
9':0.285738,GB_GCA_011355205.1:0.187238)'1.0:f__RBG-13-38-9':0.158966)'1.0:o__40CM-2-53-
6':0.105324,GB_GCA_001775995.1:0.448408)0.883:0.0410714,((((GB_GCA_001593855.1:0.0283643,
GB_GCA_003662305.1:0.0266534)1.0:0.0723184,GB_GCA_003662485.1:0.0932825)1.0:0.0785716,G
B_GCA_003662515.1:0.0967174)'1.0:g__B25':0.138948,((GB_GCA_002254975.1:0.115136,GB_GCA_
003661965.1:0.115504)'1.0:g__EX4484-
218':0.104584,GB_GCA_002011035.1:0.270775)0.746:0.0579171)'1.0:o__B25;f__B25':0.176698)0.98
6:0.043707)1.0:0.0474073,(((((GB_GCA_011369595.1:0.0224009,GB_GCA_011374155.1:0.0308284)1
.0:0.0280006,GB_GCA_011361905.1:0.0523373)1.0:0.0375215,GB_GCA_011366665.1:0.0827091)'1.
0:g__DTBU01':0.124738,GB_GCA_001593865.1:0.127378)'1.0:f__B24':0.229452,(GB_GCA_01136727
5.1:0.103114,GB_GCA_011375775.1:0.0900581)'1.0:f__DRYS01;g__DRYS01':0.33382)'1.0:o__B24':0.
162188)'0.929:c__Bathyarchaeia':0.0302349)0.0:0.0208924,GB_GCA_003661605.1:0.715771)0.999:0
.0371071,((((((((((((((((GB_GCA_003086435.1:0.00823217,RS_GCF_009729015.1:0.00362544)0.967:0.
00485736,RS_GCF_009729545.1:0.00591829)1.0:0.11714,(RS_GCF_002116695.1:0.0610672,RS_GCF
_003201765.2:0.0619962)1.0:0.0977423)1.0:0.0433901,RS_GCF_000632495.1:0.229429)'1.0:g__Acid
ianus':0.0473455,(GB_GCA_000565255.1:0.146202,RS_GCF_003201835.2:0.149366)1.0:0.0789903)0
.915:0.0305294,((((RS_GCF_000016605.1:0.0767535,RS_GCF_003201675.2:0.110695)0.993:0.02944
93,RS_GCF_000204925.1:0.136735)1.0:0.0351878,RS_GCF_013343295.1:0.140513)1.0:0.0576084,RS
_GCF_000243315.1:0.149201)'1.0:g__Metallosphaera':0.11938)1.0:0.0666646,((((RS_GCF_00002248
5.1:0.024465,RS_GCF_009601705.1:0.0284173)0.916:0.0130733,RS_GCF_900079115.1:0.0288142)1.
0:0.103727,RS_GCF_001719125.1:0.134981)'1.0:g__Saccharolobus':0.0999602,((GB_GCA_00249584
5.1:0.0363228,RS_GCF_008326425.1:0.0297505)1.0:0.0605289,RS_GCF_001316045.1:0.0973346)'1.
0:g__Sulfuracidifex':0.23397)0.999:0.0468223)0.996:0.0388307,((((RS_GCF_000012285.1:0.0073791
9,GB_GCA_000508305.1:0.00721012)'1.0:g__Sulfolobus':0.192307,(GB_GCA_003086555.1:0.013601
1,RS_GCF_009729035.1:0.0144565)'1.0:g__Stygiolobus':0.133428)0.999:0.0448228,((RS_GCF_00001
1205.1:0.00568561,RS_GCF_009729055.1:0.00431537)1.0:0.00936283,RS_GCF_012222305.1:0.0182
625)'1.0:g__Sulfurisphaera':0.129817)1.0:0.0661558,GB_GCA_000389735.1:0.331382)0.942:0.04211
)1.0:0.063073,RS_GCF_003967175.1:0.370161)'1.0:f__Sulfolobaceae':0.259138,((RS_GCF_00001794

5.1:0.0906073,GB_GCA_013154085.1:0.104277)'1.0:g__Ignicoccus_A':0.120147,RS_GCF_001481685.
1:0.196076)'1.0:f__Ignicoccaceae':0.306391)1.0:0.0555335,(((((((((GB_GCA_011361755.1:0.0124101
,GB_GCA_011380255.1:0.0139292)1.0:0.0709051,GB_GCA_011055355.1:0.092932)1.0:0.170913,(GB
_GCA_011366635.1:0.100676,GB_GCA_011375585.1:0.105395)1.0:0.13085)0.623:0.0408396,GB_GC
A_000145985.1:0.209948)0.981:0.0403426,((GB_GCA_011057405.1:0.142161,GB_GCA_011366545.
1:0.171651)1.0:0.0673879,(GB_GCA_011362275.1:0.0555505,GB_GCA_011364155.1:0.0318162)1.0:
0.133545)1.0:0.0831796)'1.0:g__Ignisphaera':0.114201,GB_GCA_013154235.1:0.27032)1.0:0.072132
4,(GB_GCA_003661775.1:0.110942,GB_GCA_003661865.1:0.113678)'1.0:g__QMWT01':0.145898)1.0
:0.0975489,GB_GCA_003661905.1:0.325093)'1.0:f__Ignisphaeraceae':0.0744778,((((((GB_GCA_0113
61945.1:0.0124809,GB_GCA_011375725.1:0.0123744)1.0:0.0555868,GB_GCA_011364565.1:0.06082
36)1.0:0.166721,GB_GCA_003056265.1:0.184411)'1.0:g__Zestosphaera':0.144831,(GB_GCA_002255
065.1:0.249932,GB_GCA_003661745.1:0.149788)'0.999:g__EX4484-
204':0.072533)0.997:0.0441272,(GB_GCA_011373125.1:0.0603425,GB_GCA_011375535.1:0.034702
6)'1.0:g__DRZP01':0.307342)0.978:0.0495447,(GB_GCA_011337845.1:0.133785,GB_GCA_011366425
.1:0.148557)'1.0:g__DTCV01':0.179532)'1.0:f__NBVN01':0.130426)0.704:0.0492327,((RS_GCF_00311
6855.1:0.122323,GB_GCA_011056395.1:0.141211)'1.0:g__AG1':0.180614,GB_GCA_009903825.1:0.2
97353)'1.0:f__AG1':0.198452)1.0:0.0662155)0.518:0.0248174,((((((((GB_GCA_002495025.1:0.102627
,GB_GCA_011056815.1:0.1319)1.0:0.0643737,(GB_GCA_011364525.1:0.13058,GB_GCA_011375785.
1:0.138571)1.0:0.0571035)1.0:0.0543176,(GB_GCA_011380145.1:0.116515,GB_GCA_011380285.1:0.
10233)1.0:0.110374)'1.0:g__UBA285':0.10328,((RS_GCF_000092185.1:0.0380324,GB_GCA_0110538
55.1:0.0300901)'1.0:g__Thermosphaera':0.205497,(RS_GCF_000186365.1:0.1024,RS_GCF_00051385
5.1:0.104203)'1.0:g__Desulfurococcus':0.111831)0.976:0.0472569)1.0:0.057966,(RS_GCF_00026449
5.1:0.106917,GB_GCA_011375875.1:0.112436)'1.0:g__Thermogladius':0.198005)1.0:0.0653536,((((R
S_GCF_000015945.1:0.0236202,RS_GCF_000092465.1:0.0355023)'1.0:g__Staphylothermus':0.11819
4,GB_GCA_013154205.1:0.188159)0.999:0.0565182,((GB_GCA_003661825.1:0.188112,GB_GCA_011
364225.1:0.236354)0.703:0.0343251,GB_GCA_002254665.1:0.148238)1.0:0.0547756)0.426:0.05305
98,GB_GCA_011366735.1:0.330249)0.997:0.0460976)'1.0:f__Desulfurococcaceae':0.215014,(((GB_G
CA_002899805.1:0.120624,GB_GCA_011333245.1:0.132587)'1.0:g__ARK-
14':0.143385,RS_GCF_000258425.1:0.231329)1.0:0.168123,(GB_GCA_009904015.1:0.326418,GB_GC
A_011380215.1:0.287015)0.89:0.0677577)'1.0:f__Fervidicoccaceae':0.170114)0.992:0.0444735,((((((
RS_GCF_000144915.1:0.0951155,RS_GCF_003431325.1:0.102464)1.0:0.101165,GB_GCA_002496425
.1:0.207048)0.218:0.0336262,GB_GCA_000495735.1:0.153368)'1.0:g__Acidilobus':0.138082,((RS_GC
F_000317795.1:0.0373814,GB_GCA_002506595.1:0.034106)1.0:0.115188,GB_GCA_002877855.1:0.1
36634)'1.0:g__Caldisphaera':0.180767)1.0:0.143943,(((RS_GCF_000011125.1:0.0411833,RS_GCF_00
0591035.1:0.0319441)'1.0:g__Aeropyrum':0.263497,GB_GCA_013152575.1:0.273619)0.961:0.05309
23,GB_GCA_011367065.1:0.347372)0.999:0.0505382)'1.0:f__Acidilobaceae':0.190598,(((((RS_GCF_0
01412615.1:0.0801246,RS_GCF_001462395.1:0.0909984)'1.0:g__Pyrodictium':0.0556268,RS_GCF_0
00015145.1:0.133854)0.905:0.0355706,((GB_GCA_003978665.1:0.234304,GB_GCA_013540595.1:0.1
812)0.855:0.0367422,GB_GCA_013153105.1:0.14184)'0.678:g__QNYQ01':0.02883)1.0:0.0994548,GB
_GCA_013153145.1:0.235622)1.0:0.0707192,RS_GCF_000223395.1:0.245068)'1.0:f__Pyrodictiaceae'
:0.137327)1.0:0.0606627)0.76:0.0297734)'1.0:o__Sulfolobales':0.0621807,(GB_GCA_002254745.1:0.
166335,GB_GCA_011375895.1:0.156348)'1.0:o__EX4484-217-1;f__EX4484-217-
1':0.234649)1.0:0.0890444,((((GB_GCA_003019535.1:0.0112046,RS_GCF_902812505.1:0.0109385)'1
.0:g__ECH-B-
1':0.283015,GB_GCA_003019595.1:0.269733)'1.0:f__Marsarchaeaceae':0.370739,(GB_GCA_0025070
85.1:0.0238194,GB_GCA_011055275.1:0.0358303)'1.0:f__UBA168;g__UBA168':0.726108)'1.0:o__M
arsarchaeales':0.128953,((GB_GCA_000375685.1:0.260755,GB_GCA_000380705.1:0.228095)0.547:0.
0545175,GB_GCA_011056475.1:0.255552)'1.0:o__Gearchaeales;f__Gearchaeaceae':0.439207)0.868:

0.0677825)0.888:0.0404393,(((((((((((((RS_GCF_000148385.1:0.00918663,GB_GCA_001316125.1:0.0
232619)1.0:0.0161734,(GB_GCA_001316245.1:0.0318132,GB_GCA_002506645.1:0.0664222)0.989:0.
0127752)0.6:0.00556245,(GB_GCA_001316005.1:0.027709,GB_GCA_001316285.1:0.0230894)0.7:0.0
0479023)0.969:0.0106289,(GB_GCA_001315985.1:0.00919554,GB_GCA_001316265.1:0.00722522)1.
0:0.0390225)0.999:0.0190691,RS_GCF_000190315.1:0.0549597)0.679:0.0170497,(GB_GCA_0013160
25.1:0.0416606,GB_GCA_002496475.1:0.0423521)1.0:0.0572261)1.0:0.0276449,GB_GCA_00151676
5.1:0.0779425)'1.0:g__Vulcanisaeta':0.0594024,RS_GCF_001748385.1:0.113532)1.0:0.152799,(((RS_
GCF_000018305.1:0.0276203,GB_GCA_002506515.1:0.0425426)1.0:0.0692944,RS_GCF_001663375.
1:0.0762846)'1.0:g__Caldivirga':0.225798,((RS_GCF_001315925.1:0.0261594,GB_GCA_002508315.1:
0.0301341)1.0:0.0844619,GB_GCA_001516585.1:0.119114)'1.0:g__Thermocladium':0.269942)1.0:0.
104039)0.954:0.0532992,(((GB_GCA_013153895.1:0.0309829,GB_GCA_013154355.1:0.0309525)1.0:
0.0273717,GB_GCA_013153275.1:0.0584816)'1.0:g__JAADER01':0.232153,GB_GCA_002254895.1:0.
202709)0.999:0.0654834)'1.0:f__Thermocladiaceae':0.0711488,(((((RS_GCF_000007225.1:0.0716895
,RS_GCF_000234805.1:0.0810061)0.616:0.0164714,(RS_GCF_000016385.1:0.00843531,GB_GCA_000
247545.1:0.00817235)1.0:0.0997817)0.983:0.0230358,((RS_GCF_000015205.1:0.0993974,RS_GCF_0
00019805.1:0.0896136)1.0:0.0336976,(GB_GCA_001189275.1:0.0105742,GB_GCA_009903905.1:0.0
0633679)1.0:0.0829621)0.949:0.0142738)1.0:0.0350642,GB_GCA_000015805.1:0.0936788)'1.0:g__P
yrobaculum':0.15342,(((((RS_GCF_002077075.2:0.00352967,GB_GCA_009903945.1:0.00443434)1.0:
0.0544955,RS_GCF_000193375.1:0.0388324)1.0:0.0430362,GB_GCA_002497345.1:0.0927873)0.999:
0.03533,RS_GCF_000253055.1:0.123333)1.0:0.0549704,GB_GCA_000960885.1:0.125562)'1.0:g__Th
ermoproteus':0.110209)'1.0:f__Thermoproteaceae':0.241459)1.0:0.178477,GB_GCA_003649205.1:0.
296348)'1.0:o__Thermoproteales':0.107429,(((((((RS_GCF_000015225.1:0.134573,RS_GCF_0008132
45.1:0.161249)'1.0:g__Thermofilum_B':0.103187,(RS_GCF_000993805.1:0.151493,GB_GCA_002855
745.1:0.119706)'1.0:g__Thermofilum_A':0.114661)0.967:0.040331,(((GB_GCA_011047595.1:0.06971
37,GB_GCA_011372985.1:0.0381894)0.959:0.0148832,GB_GCA_011363115.1:0.0459136)1.0:0.0890
971,GB_GCA_011364825.1:0.181885)'1.0:g__Thermofilum_C':0.0925681)1.0:0.193966,(((GB_GCA_0
11053655.1:0.0148365,GB_GCA_011369665.1:0.0193275)1.0:0.0797114,(GB_GCA_011055445.1:0.0
646717,GB_GCA_011372935.1:0.0537828)1.0:0.0482151)'1.0:g__DRZQ01':0.123407,GB_GCA_00364
9495.1:0.149546)1.0:0.169408)'1.0:f__Thermofilaceae':0.081062,GB_GCA_003649235.1:0.321312)0.
99:0.0612658,GB_GCA_003649225.1:0.350361)0.982:0.046593,(((GB_GCA_003649515.1:0.174728,G
B_GCA_003650675.1:0.196415)1.0:0.12704,GB_GCA_003650785.1:0.305682)'0.885:f__B20-
G17':0.0496606,GB_GCA_002254595.1:0.354237)0.819:0.0678944)1.0:0.0491428,(GB_GCA_003649
445.1:0.0986002,GB_GCA_003650815.1:0.104313)'1.0:f__B15-G2;g__B15-
G2':0.267384)'0.887:o__Thermofilales':0.0480429)0.989:0.038424,(GB_GCA_003649665.1:0.220595,
GB_GCA_003650925.1:0.214686)'1.0:o__QMSL01;f__QMSL01':0.167341)1.0:0.063963)'0.999:c__The
rmoproteia':0.0398798,((((((((GB_GCA_001717015.1:0.0408551,GB_GCA_004028775.1:0.0298926)1.
0:0.0311928,GB_GCA_014361455.1:0.07651)'1.0:g__Methanosuratus':0.148108,((GB_GCA_0017170
25.1:0.0669943,GB_GCA_001717035.1:0.0533075)1.0:0.152177,GB_GCA_011329225.1:0.155727)'1.
0:g__Methanomethylicus':0.0594703)1.0:0.116923,((GB_GCA_004347955.1:0.0130877,GB_GCA_011
362965.1:0.0173656)0.986:0.0103001,GB_GCA_014361445.1:0.0180721)'1.0:g__WYZ-
LMO10':0.12292)1.0:0.102921,GB_GCA_004348015.1:0.184535)'1.0:f__Methanomethylicaceae':0.19
5182,GB_GCA_003649085.1:0.284904)'1.0:o__Methanomethylicales':0.130961,((((((GB_GCA_01136
2155.1:0.0491463,GB_GCA_011369545.1:0.0681307)0.666:0.0137688,GB_GCA_011605835.1:0.0493
138)1.0:0.0510583,GB_GCA_004347965.1:0.108342)1.0:0.0470301,GB_GCA_003649565.1:0.100724)
0.979:0.0458878,GB_GCA_003650865.1:0.140357)'1.0:f__WYZ-LMO8;g__WYZ-
LMO8':0.27956,((GB_GCA_003649615.1:0.151594,GB_GCA_011375665.1:0.196876)'1.0:f__B40-
G2':0.127408,GB_GCA_011364865.1:0.324763)1.0:0.103988)'1.0:o__Nezhaarchaeales':0.0963609)0.
048:0.0297805,((((((GB_GCA_011370515.1:0.00239089,GB_GCA_011605825.1:0.00734848)1.0:0.049

9465,GB_GCA_011372955.1:0.0400317)1.0:0.09193,GB_GCA_011367155.1:0.140986)'1.0:f__DSZF01;g__DSZF01':0.188105,GB_GCA_003649185.1:0.211664)0.997:0.0616293,(GB_GCA_003649145.1:0.0727549,GB_GCA_003650625.1:0.0686876)'1.0:f__B29-G17;g__B29-G17':0.13545)'1.0:o__B29-G17':0.132883,GB_GCA_011362245.1:0.514233)0.991:0.0558243)'1.0:c__Methanomethylicia':0.0386064)1.0:0.0590834)0.909:0.0352702,((((((RS_GCF_000019605.1:0.0215259,GB_GCA_011056255.1:0.0215464)1.0:0.0975712,GB_GCA_003344655.1:0.110434)'1.0:g__Korarchaeum':0.230394,RS_GCF_003947435.1:0.356009)0.994:0.0640975,GB_GCA_011042755.1:0.303762)'0.997:f__Korarchaeaceae':0.067022,(GB_GCA_003661265.1:0.0590471,GB_GCA_003661365.1:0.0909001)'1.0:f__QMVU01;g__QMVU01':0.282204)1.0:0.191393,GB_GCA_003661385.1:0.503688)'1.0:o__Korarchaeales':0.173988,GB_GCA_011041895.1:0.587591)'1.0:c__Korarchaeia':0.0777545)'1.0:p__Thermoproteota':0.0499309,(((((((((((((((GB_GCA_013375475.1:0.0508308,GB_GCA_013375495.1:0.0515576)1.0:0.0233108,GB_GCA_011364925.1:0.0855293)1.0:0.0322389,GB_GCA_004375715.1:0.113981)1.0:0.0417026,GB_GCA_005222975.1:0.0960548)1.0:0.0503401,GB_GCA_004524725.1:0.189414)0.959:0.0254441,GB_GCA_011364975.1:0.166235)1.0:0.0444622,(GB_GCA_005223125.1:0.0708932,GB_GCA_013375485.1:0.0542086)1.0:0.100866)'0.999:g__SOKP01':0.0420582,((GB_GCA_004524385.1:0.0241179,GB_GCA_004524535.1:0.0289717)1.0:0.13623,(GB_GCA_004524425.1:0.141419,GB_GCA_004524515.1:0.132909)0.0:0.0253125)'1.0:g__SDNM01':0.0410136)1.0:0.0700794,((GB_GCA_004524365.1:0.162613,GB_GCA_008080765.1:0.160892)1.0:0.047894,GB_GCA_008080735.1:0.138784)'1.0:g__TEKIR-8':0.0736804)'1.0:f__SOKP01':0.266149,(GB_GCA_004524545.1:0.398333,RS_GCF_008000775.1:0.380467)'1.0:f__AMARA-1':0.144072)0.946:0.0708222,GB_GCA_001940655.1:0.45791)'1.0:o__CR-4':0.213388,((((GB_GCA_013375405.1:0.0920152,GB_GCA_013375455.1:0.0957808)1.0:0.0600245,GB_GCA_005191415.1:0.0984183)'1.0:f__HEL-GB-A;g__HEL-GB-A':0.231224,(GB_GCA_011365055.1:0.367901,GB_GCA_013375355.1:0.328392)1.0:0.0987039)0.999:0.0724461,GB_GCA_005191425.1:0.454556)'1.0:o__Helarchaeales':0.0940989)'0.958:c__Lokiarchaeia':0.0537259,(((((((((GB_GCA_001563335.1:0.0400922,GB_GCA_002825515.1:0.0446196)1.0:0.0323418,(GB_GCA_001940705.1:0.0547159,GB_GCA_011364905.1:0.0988713)0.997:0.0207203)1.0:0.0325259,GB_GCA_004376265.1:0.090753)'1.0:g__SMTZ1-45':0.0487061,(((GB_GCA_002825465.1:0.0107313,GB_GCA_002825535.1:0.0118333)1.0:0.0673138,GB_GCA_004524565.1:0.07903)1.0:0.0481879,(GB_GCA_003345545.1:0.143639,GB_GCA_004524595.1:0.108551)0.405:0.0232479)'1.0:g__MP8T-1':0.0359363)1.0:0.0696589,((GB_GCA_011364985.1:0.0615333,GB_GCA_011365025.1:0.0552635)1.0:0.0979461,GB_GCA_001563325.1:0.12165)'1.0:g__SMTZ1-83':0.0422493)0.94:0.02211,GB_GCA_013138615.1:0.179827)0.988:0.023439,((GB_GCA_004524445.1:0.203091,GB_GCA_008080745.1:0.178617)0.937:0.0300781,GB_GCA_004524435.1:0.201359)0.982:0.0254747)0.985:0.0222144,((GB_GCA_003345555.1:0.118058,GB_GCA_003345595.1:0.116135)'1.0:g__OWC5':0.0966961,GB_GCA_013388835.1:0.215963)1.0:0.0423069)0.864:0.032795,GB_GCA_003662765.1:0.124647)'1.0:c__Thorarchaeia;o__Thorarchaeales;f__Thorarchaeaceae':0.473224)0.944:0.0419511,((GB_GCA_011362025.1:0.0759955,GB_GCA_011364305.1:0.0566994)'1.0:c__DTBI01;o__DTBI01;f__DTBI01;g__DTBI01':0.3803,GB_GCA_001940665.1:0.468218)0.671:0.0557708)0.985:0.0362071,(((((GB_GCA_001940755.1:0.0754379,GB_GCA_002505645.1:0.0608921)1.0:0.107419,GB_GCA_013166775.1:0.143592)'1.0:o__UBA460;f__UBA460;g__UBA460':0.365073,(GB_GCA_001940725.1:0.3126,GB_GCA_002728275.1:0.317469)'1.0:o__LC-2;f__LC-2':0.351819)1.0:0.115353,GB_GCA_013166835.1:0.546294)0.973:0.0593404,((GB_GCA_003144275.1:0.19517,GB_GCA_011364965.1:0.188269)'1.0:f__B3-JM-08;g__B3-JM-08':0.313213,GB_GCA_001940645.1:0.558601)'1.0:o__LC-3':0.225831)'1.0:c__Heimdallarchaeia':0.111926)'1.0:p__Asgardarchaeota':0.0592267)1.0:0.0777952)0.995:0.047286,((((((GB_GCA_003663045.1:0.147869,GB_GCA_903930485.1:0.273701)1.0:0.0832387,(GB_GCA_003663115.1:0.179315,GB_GCA_003663175.1:0.239032)0.979:0.0458085)0.895:0.03717

61,(GB_GCA_002254405.1:0.167711,GB_GCA_002254565.1:0.134097)'1.0:g__EX4484-
2':0.236445)'1.0:f__QMZM01':0.0544116,(((GB_GCA_001723835.1:0.0925582,GB_GCA_001723855.
1:0.0342431)'1.0:g__WOR-SM1-SCG':0.248438,GB_GCA_001723845.1:0.284548)'1.0:f__WOR-SM1-
SCG':0.0752851,GB_GCA_001742785.1:0.403922)0.994:0.0423213)0.807:0.0455816,((GB_GCA_0004
02775.1:0.333653,GB_GCA_011380095.1:0.37914)0.761:0.0588986,GB_GCA_902384455.1:0.433123
)'1.0:f__SCGC-AAA252-
I15':0.104269)'1.0:o__IMC4':0.0953698,((GB_GCA_001873845.1:0.0815864,GB_GCA_002083985.1:0
.0598198)0.996:0.0392134,GB_GCA_002841105.1:0.0593823)'1.0:o__Altarchaeales;f__Altarchaeace
ae;g__Altarchaeum':0.553157)'1.0:p__Altarchaeota;c__Altarchaeia':0.189482)0.995:0.0495342,((((((
(((((GB_GCA_013425375.1:0.148967,GB_GCA_902386555.1:0.166784)1.0:0.0652252,GB_GCA_9038
35325.1:0.220965)'1.0:g__CAILMU01':0.095976,GB_GCA_903835385.1:0.285348)1.0:0.0610463,((GB
_GCA_011358535.1:0.12503,GB_GCA_013425425.1:0.150325)'1.0:g__JACCLJ01':0.106982,GB_GCA_
011358475.1:0.292678)1.0:0.0748057)1.0:0.063705,GB_GCA_013331535.1:0.343782)1.0:0.107594,((
GB_GCA_013425455.1:0.0296454,GB_GCA_903920925.1:0.032562)'1.0:g__CAIXOA01':0.256206,GB_
GCA_903828955.1:0.289766)1.0:0.158927)'0.998:f__UBA10214':0.0650934,(((GB_GCA_011380495.1
:0.480269,GB_GCA_013153395.1:0.294926)'1.0:g__JAADEL01':0.310099,GB_GCA_003694965.1:0.49
0141)1.0:0.117006,GB_GCA_011362975.1:0.328145)'1.0:f__J096':0.124504)1.0:0.123112,((((((GB_G
CA_902384555.1:0.165707,GB_GCA_903923795.1:0.159197)'1.0:g__CAIXYX01':0.163157,GB_GCA_0
02778455.1:0.292976)0.704:0.0848487,GB_GCA_011334515.1:0.427466)1.0:0.126311,((GB_GCA_01
3331805.1:0.186934,GB_GCA_903892905.1:0.200576)'1.0:g__UBA10161':0.186638,GB_GCA_90238
4585.1:0.305099)0.999:0.0746706)'0.992:f__UBA10161':0.0560579,GB_GCA_001871475.1:0.411997
)0.977:0.0536797,GB_GCA_001871535.1:0.635166)0.786:0.0351844)'1.0:o__UBA10214':0.0629643,(
((((GB_GCA_013425335.1:0.0526063,GB_GCA_013425405.1:0.0557959)1.0:0.0772505,GB_GCA_013
426015.1:0.108129)'1.0:g__Sv326':0.0711198,GB_GCA_011335015.1:0.104295)'1.0:f__DTGH01':0.24
8925,((GB_GCA_903884505.1:0.151278,GB_GCA_903921825.1:0.188549)1.0:0.0758985,GB_GCA_01
1358645.1:0.194947)'1.0:f__DTNL01':0.181969)'1.0:o__DTNL01':0.0610889,((GB_GCA_003660965.1:
0.180912,GB_GCA_902386535.1:0.172925)'1.0:o__B9-G16;f__B9-
G16':0.253049,GB_GCA_902384795.1:0.446593)0.921:0.0593739)1.0:0.057974)0.911:0.0413876,((G
B_GCA_011332735.1:0.232267,GB_GCA_902385155.1:0.251117)'1.0:o__CABMEP01;f__CABMEP01':
0.340617,(GB_GCA_011389055.1:0.11209,GB_GCA_903832205.1:0.15308)'1.0:o__CAILAH01;f__CAIL
AH01;g__CAILAH01':0.489511)0.967:0.0600638)0.645:0.0339397,((((GB_GCA_001889985.1:0.33647
1,GB_GCA_002501775.1:0.273324)0.967:0.0618917,RS_GCF_002214165.1:0.387245)1.0:0.0880675,(
(GB_GCA_013330215.1:0.23601,GB_GCA_903858385.1:0.263586)0.978:0.0484265,GB_GCA_903884
115.1:0.332681)1.0:0.0796736)1.0:0.132991,GB_GCA_902385515.1:0.437043)'1.0:o__Micrarchaeale
s;f__Micrarchaeaceae':0.328833)1.0:0.0597563,((((((GB_GCA_002499405.1:0.378197,GB_GCA_0027
92915.1:0.32373)1.0:0.122299,(GB_GCA_002763345.1:0.0641344,GB_GCA_902384935.1:0.0731052)
'1.0:g__0-14-0-20-59-
11':0.286948)'0.959:f__UBA93':0.0540371,GB_GCA_903893865.1:0.449053)0.933:0.0525407,(GB_G
CA_013331595.1:0.368024,GB_GCA_013360305.1:0.367664)'1.0:f__UBA10210':0.0974901)1.0:0.056
8611,(((GB_GCA_001871495.1:0.188082,GB_GCA_013425325.1:0.134757)'1.0:f__UBA8480;g__UBA8
480':0.227725,GB_GCA_011358415.1:0.577421)1.0:0.07497,(GB_GCA_001871595.1:0.480109,GB_G
CA_005239845.1:0.390152)0.988:0.0601887)0.858:0.0418167)0.979:0.0471072,(GB_GCA_90238504
5.1:0.635655,GB_GCA_903851445.1:0.458433)0.687:0.0682135)'1.0:o__UBA8480':0.207024)'1.0:p_
_Micrarchaeota;c__Micrarchaeia':0.133777,((((((((((((GB_GCA_002499505.1:0.000376093,GB_GCA_
002687735.1:6e-
09)1.0:0.0972732,bin.647:0.100169):0.0981818,GB_GCA_002762975.1:0.242448)'0.973:g__UBA493'
:0.0456297,GB_GCA_000402355.1:0.206861)0.272:0.0317821,((GB_GCA_005239615.1:0.166295,GB
_GCA_902386385.1:0.189585)1.0:0.108254,GB_GCA_013331625.1:0.226172)'1.0:g__UBA10205':0.0

545359)'1.0:f__Iainarchaeaceae':0.165102,GB_GCA_000830275.1:0.295413)0.309:0.0533629,GB_GC
A_013331455.1:0.410481)1.0:0.0767796,(GB_GCA_002763225.1:0.401663,GB_GCA_013331925.1:0.
413446)0.932:0.0607307)0.811:0.0399143,((GB_GCA_002779065.1:0.40888,(GB_GCA_011334955.1:
0.36564,bin.350:0.373148):0.0990482)0.999:0.0804978,GB_GCA_002688035.1:0.371361)0.898:0.04
66289)0.999:0.059417,(((GB_GCA_002068885.1:0.184063,GB_GCA_902385225.1:0.200654)1.0:0.09
13761,GB_GCA_012799645.1:0.221159)'1.0:f__JAAZKV01;g__JAAZKV01':0.0560695,bin.104:0.31078
4):0.278732)'0.999:o__Iainarchaeales':0.0720408,((GB_GCA_002779075.1:0.164309,GB_GCA_90391
6665.1:0.173327)'1.0:f__0-14-0-20-30-16;g__0-14-0-20-30-
16':0.62513,(GB_GCA_003559395.1:0.340589,GB_GCA_009392915.1:0.317833)'1.0:f__CSSED10-
239':0.40133)'0.976:o__0-14-0-20-30-
16':0.10477)1.0:0.113667,(GB_GCA_013154035.1:0.356126,GB_GCA_013154145.1:0.346339)'1.0:o_
_JAADDC01;f__JAADDC01':0.31227)1.0:0.0750434,(((GB_GCA_003551125.1:0.13935,GB_GCA_00355
3825.1:0.168774)'1.0:g__B1Sed10-29':0.166784,GB_GCA_003558235.1:0.29543)'1.0:f__B1Sed10-
29':0.285605,GB_GCA_003660975.1:0.436658)'1.0:o__B1Sed10-
29':0.176694)'0.544:p__Iainarchaeota;c__Iainarchaeia':0.041542)1.0:0.0535239)0.955:0.0352083,((((
GB_GCA_002495465.1:0.0243112,GB_GCA_014189675.1:0.0181501)1.0:0.0441479,GB_GCA_002687
935.1:0.0587439)'1.0:f__Undinarchaeaceae;g__Undinarchaeum':0.375487,GB_GCA_002502135.1:0.
375975)0.996:0.0715162,GB_GCA_014189685.1:0.362393)'1.0:p__Undinarchaeota;c__Undinarchaei
a;o__Undinarchaeales':0.293043)1.0:0.0422431,(((((((((((((((((((GB_GCA_003560545.1:0.0924429,GB
_GCA_003562145.1:0.0817593)0.0:0.0171405,GB_GCA_007131205.1:0.0591036)'1.0:g__PWWA01':
0.19744,(GB_GCA_002762915.1:0.241499,GB_GCA_903891505.1:0.295191)'0.998:g__21-14-0-10-
32-
9':0.0646575)0.78:0.0513523,((GB_GCA_007116295.1:0.293486,GB_GCA_007117755.1:0.305851)'0.
813:g__SKIS01':0.0483479,GB_GCA_007117145.1:0.296104)1.0:0.092188)'1.0:f__21-14-0-10-32-
9':0.16347,(((((GB_GCA_013202845.1:8.53103e-06,bin.69:6.11352e-
06):0.139763,GB_GCA_014384375.1:0.132539)1.0:0.047998,GB_GCA_002686295.1:0.236095)1.0:0.0
605243,GB_GCA_014384455.1:0.194199)'1.0:f__GCA-2686295;g__GCA-
2686295':0.161087,(GB_GCA_001595785.1:0.139118,GB_GCA_011372335.1:0.11157)'1.0:f__SM23-
78;g__SM23-
78':0.232422)0.999:0.051677)0.458:0.0300497,((GB_GCA_011329125.1:0.123152,GB_GCA_0133305
95.1:0.157884)'1.0:g__UBA9642':0.256357,GB_GCA_003599145.1:0.39194)'1.0:f__UBA9642':0.0911
536)0.0:0.0163775,(((((GB_GCA_002498125.1:0.174454,GB_GCA_013331575.1:0.19223)'1.0:g__UBA
525':0.25408,(GB_GCA_002503705.1:0.277599,GB_GCA_003694385.1:0.264822)'1.0:g__UBA153':0.
169074)'1.0:f__UBA525':0.0850811,GB_GCA_007117735.1:0.476827)0.0:0.0376805,((GB_GCA_0071
16645.1:0.100142,GB_GCA_007128245.1:0.0903601)1.0:0.222851,GB_GCA_007117065.1:0.36062)'1
.0:f__SKIA01;g__SKIA01':0.293086)0.983:0.0506458,(GB_GCA_002685855.1:0.404097,GB_GCA_0036
94805.1:0.435294)0.994:0.0739742)1.0:0.0524383)1.0:0.07304,(((GB_GCA_000830295.1:0.228902,G
B_GCA_013335235.1:0.272571)1.0:0.193234,GB_GCA_003564925.1:0.336959)0.994:0.0570221,(GB
_GCA_002762705.1:0.465755,GB_GCA_002867475.1:0.41059)0.982:0.0716981)0.973:0.0401993)0.9
87:0.035982,(((((GB_GCA_013203345.1:6.76572e-06,bin.595:6.11352e-
06):0.221692,GB_GCA_013331125.1:0.26732)1.0:0.121128,GB_GCA_003695435.1:0.442464)'1.0:f__
UBA11576':0.0889473,(GB_GCA_002762985.1:0.309061,GB_GCA_013331695.1:0.409153)'1.0:f__UB
A12501':0.0941639)0.34:0.0303446,((((GB_GCA_903904885.1:0.343512,GB_GCA_903907555.1:0.33
8027)0.853:0.0454285,GB_GCA_903842985.1:0.304025)'1.0:f__CAIVOO01':0.0956074,GB_GCA_013
331965.1:0.507916)0.974:0.0346193,GB_GCA_003650545.1:0.425337)0.996:0.0423756)1.0:0.03487
86)0.655:0.0230362,(((((GB_GCA_002686855.1:0.157678,GB_GCA_002688925.1:0.191728)1.0:0.122
975,(GB_GCA_002688775.1:0.246619,GB_GCA_013330825.1:0.396421)0.416:0.0541067)'1.0:f__GW
2011-

AR4':0.097858,((GB_GCA_002687795.1:0.315374,(GB_GCA_005222965.1:0.290266,bin.78:0.32753):0.0314072)'0.999:f__GCA-
2687795':0.0574163,GB_GCA_013329175.1:0.46828)0.958:0.0487789)0.994:0.038818,(((GB_GCA_002762865.1:0.0664961,bin.482:0.0706623):0.100304,GB_GCA_003561825.1:0.269421)'1.0:f__CG1-02-33-
12':0.169242,GB_GCA_013329045.1:0.526027)0.79:0.0422758)0.946:0.0289018,(((GB_GCA_003695265.1:0.324396,GB_GCA_013331615.1:0.402894)0.935:0.0470921,((GB_GCA_013152815.1:0.381909,bin.101:0.322798):0.0614523,((GB_GCA_013202955.1:5.02714e-06,bin.305:6.11352e-
06):0.197745,bin.458:0.177167):0.131709)0.942:0.0564028)0.969:0.0339753,(GB_GCA_003695045.1:0.39089,GB_GCA_011368255.1:0.431028)0.999:0.0582598)0.996:0.03729)1.0:0.0358123)0.991:0.030582,((((((((GB_GCA_002762795.1:0.319292,GB_GCA_902385765.1:0.234166)0.73:0.0628676,(GB_GCA_003599055.1:0.260962,GB_GCA_003694525.1:0.255498)0.987:0.0688723)1.0:0.132921,GB_GCA_011331795.1:0.406898)0.905:0.0569497,GB_GCA_002687275.1:0.502226)'0.995:f__ARS49':0.0689467,GB_GCA_001871415.1:0.616587)0.554:0.0389737,GB_GCA_003648985.1:0.466302)0.74:0.0292533,(((GB_GCA_002688315.1:0.107539,bin.219:0.121932):0.175523,GB_GCA_013331515.1:0.362831)'1.0:f__UBA10216':0.139781,GB_GCA_002763335.1:0.454005)1.0:0.0695753)0.948:0.0375738,GB_GCA_002779235.1:0.519237)0.0:0.0254134)0.954:0.0296253,((GB_GCA_002505945.1:0.48313,GB_GCA_011045925.1:0.499384)'1.0:f__UBA119':0.124848,(GB_GCA_003650585.1:0.217482,GB_GCA_903915225.1:0.254754)'1.0:f__B29-G15;g__B29-
G15':0.277784)1.0:0.081894)0.912:0.035407,((((((GB_GCA_002686315.1:0.00158191,GB_GCA_013330875.1:0.00667316)1.0:0.254673,GB_GCA_002762785.1:0.199419)'1.0:g__UBA11998':0.0731644,(GB_GCA_002763025.1:0.0883453,(GB_GCA_013204355.1:9.7929e-06,bin.502:6.11352e-
06):0.0802137)'1.0:g__1-14-0-10-32-
24':0.207946)0.755:0.0309912,((GB_GCA_002762845.1:0.232294,GB_GCA_002792115.1:0.231293)0.998:0.0486641,(GB_GCA_013331555.1:0.299518,GB_GCA_013331635.1:0.251439)0.99:0.0544134)0.62:0.0273442)1.0:0.0907686,((GB_GCA_002688265.1:0.337154,GB_GCA_902529885.1:0.507602)1.0:0.052789,bin.210:0.269132):0.051479)0.813:0.05373,GB_GCA_002687775.1:0.383459)'1.0:f__GW2011-
AR9':0.213954)'1.0:o__Woesearchaeales':0.0632696,((((((((((((((((GB_GCA_002762735.1:0.112412,GB_GCA_009992175.1:0.132835)1.0:0.128468,GB_GCA_009992215.1:0.224121)0.998:0.0616206,GB_GCA_903831045.1:0.272924)'0.99:g__GW2011-
AR1':0.0517912,(GB_GCA_002254395.1:0.165178,bin.179:0.143965):0.115795)0.999:0.0545535,GB_GCA_000806015.1:0.288356)0.988:0.0383129,(GB_GCA_002069705.1:0.270553,GB_GCA_013329055.1:0.267432)0.203:0.0357721)0.988:0.0296906,((((GB_GCA_002689985.1:0.115402,GB_GCA_002731115.1:0.096369)'1.0:g__NP1466':0.0957638,GB_GCA_002897655.1:0.181896)1.0:0.0414285,bin.50:0.170457):0.0382334,(GB_GCA_000405225.1:0.287878,GB_GCA_002686355.1:0.263572)0.969:0.0508861)0.94:0.0276902)0.851:0.0263836,((GB_GCA_001786415.1:0.163193,GB_GCA_902386025.1:0.18605)0.13:0.053332,GB_GCA_012798335.1:0.304791)'1.0:g__RBG-13-33-
26':0.0645703)0.86:0.0297375,GB_GCA_902385635.1:0.376369)0.52:0.0323553,GB_GCA_002763085.1:0.343383)'1.0:f__GW2011-
AR1':0.110153,(GB_GCA_001872185.1:0.264784,(GB_GCA_002763155.1:0.239249,bin.77:0.256726):0.0411025)'1.0:f__PFCU01;g__PFCU01':0.173328)0.982:0.0422302,(((((((GB_GCA_002688335.1:0.193618,GB_GCA_002688875.1:0.235086)0.968:0.058776,GB_GCA_002688165.1:0.210983)'1.0:g__ARS1285':0.0526362,bin.129:0.21236):0.0429659,(GB_GCA_011333975.1:0.223259,GB_GCA_902385625.1:0.213063)0.0:0.0491291)1.0:0.116092,GB_GCA_903866625.1:0.36895)'0.922:f__ARS1285':0.0513646,GB_GCA_001786395.1:0.366512)1.0:0.0485226,(((((GB_GCA_002688415.1:0.319675,GB_GCA_003693875.1:0.351904)0.993:0.0697608,GB_GCA_002690445.1:0.245712)0.0:0.0409621,GB_GCA_903828655.1:0.268559)1.0:0.0553806,bin.665:0.277854):0.0542424,GB_GCA_902385655.1:0.348316)'

1.0:f__ARS1160':0.176781)0.709:0.0313681)0.828:0.0310766,(((((GB_GCA_002496015.1:0.316236,G
B_GCA_003694055.1:0.38418)0.927:0.0595321,(GB_GCA_002686195.1:0.246767,GB_GCA_0026880
15.1:0.285517)0.847:0.0643685)0.999:0.0507597,((GB_GCA_009694725.1:0.407663,GB_GCA_90387
3955.1:0.31472)1.0:0.105192,(GB_GCA_002687255.1:0.336217,bin.498:0.259664):0.0996205)0.219:
0.048513)1.0:0.0422508,((((GB_GCA_002497285.1:0.18485,GB_GCA_002792635.1:0.210682)0.724:0
.0443288,GB_GCA_001872315.1:0.201904)1.0:0.104336,GB_GCA_002763075.1:0.295748)0.112:0.04
83861,(GB_GCA_004195435.1:0.294397,GB_GCA_009694755.1:0.367912)0.998:0.0626681)0.993:0.0
483174)'1.0:f__UBA73':0.12934,GB_GCA_003554465.1:0.606219)0.852:0.0472784)0.872:0.0339318,
GB_GCA_002763035.1:0.499758)1.0:0.0954292,((((GB_GCA_002499185.1:0.390151,GB_GCA_01137
1765.1:0.273333)0.663:0.0535915,GB_GCA_002254805.1:0.341944)0.982:0.0642366,GB_GCA_0027
63115.1:0.482321)0.994:0.0549179,(GB_GCA_002780285.1:0.48178,GB_GCA_011362825.1:0.40528
2)0.582:0.0556056)0.827:0.0483166)1.0:0.107181,GB_GCA_013331945.1:0.510053)'1.0:o__Pacearc
haeales':0.165817,((((((GB_GCA_002687825.1:0.141919,GB_GCA_002688395.1:0.153936)'1.0:f__AR
S1403;g__ARS1403':0.215277,GB_GCA_002688095.1:0.382427)0.732:0.0611504,GB_GCA_00040251
5.1:0.391888)1.0:0.0906795,GB_GCA_002687815.1:0.390336)0.872:0.0382406,((((GB_GCA_0036944
95.1:0.326197,GB_GCA_013329215.1:0.388277)0.949:0.0718242,(((GB_GCA_009691355.1:0.136683
,GB_GCA_013331795.1:0.0992516)'1.0:g__GW2011-
AR17':0.239537,bin.391:0.353132):0.00700996,bin.497:0.298938):0.102904)'1.0:f__GW2011-
AR17':0.032589,bin.185:0.376582):0.079245,GB_GCA_002505585.1:0.505587)0.885:0.0400909)0.79
2:0.0310463,((((GB_GCA_000830315.1:0.326724,GB_GCA_002688355.1:0.386964)'1.0:f__GW2011-
AR20':0.0480021,bin.683:0.395898):0.0425888,GB_GCA_000806155.1:0.514787)0.962:0.0388706,((
GB_GCA_002687185.1:0.622681,((GB_GCA_013824915.1:0.321008,bin.312:0.3511):0.0782343,bin.3
00:0.345386):0.0366459)0.243:0.0508202,GB_GCA_002688325.1:0.456253)0.863:0.0406554)0.982:0
.043936)'1.0:o__SCGC-AAA011-
G17':0.0562115)0.234:0.0488884)0.995:0.0562356,(((GB_GCA_002506365.1:0.0746773,GB_GCA_01
3215525.1:0.0974642)'1.0:g__UBA583':0.280813,GB_GCA_007117405.1:0.225443)0.727:0.0576967,
GB_GCA_002505525.1:0.310497)'1.0:o__UBA583;f__UBA583':0.604167)0.897:0.0392991,(((((GB_GC
A_002686215.1:0.00113887,GB_GCA_002725495.1:0.000940365)1.0:0.336306,GB_GCA_014239585.
1:0.210897)'0.998:g__ARS21':0.104504,GB_GCA_014240455.1:0.277121)'1.0:f__ARS21':0.302932,bi
n.525:0.530112):0.0720123,((GB_GCA_011333955.1:0.341221,GB_GCA_903864915.1:0.293717)1.0:
0.130968,GB_GCA_013331935.1:0.392562)'1.0:f__UBA10117':0.170929)'1.0:o__UBA10117':0.18507
4)0.204:0.0297555,(((((GB_GCA_000387965.1:0.0191639,GB_GCA_003086415.1:0.0116131)0.946:0.
0140543,GB_GCA_001552015.1:0.0298617)'1.0:g__Nanopusillus':0.318205,GB_GCA_003568775.1:0.
271724)1.0:0.0885185,GB_GCA_011331665.1:0.224009)'1.0:f__Nanopusillaceae':0.247115,GB_GCA_
000008085.1:0.492661)'1.0:o__Nanoarchaeales':0.283324)0.262:0.0327501,(((((GB_GCA_003563585
.1:0.0646535,GB_GCA_007133845.1:0.0702747)'1.0:g__CSSed11-
243R1':0.317914,GB_GCA_011333755.1:0.4026)'1.0:o__CSSed11-243R1;f__CSSed11-
243R1':0.181561,(GB_GCA_003660925.1:0.437433,GB_GCA_011366705.1:0.532789)0.887:0.058761
4)0.711:0.0451485,((GB_GCA_002502045.1:0.13486,GB_GCA_002503215.1:0.171276)'1.0:o__Parvar
chaeales;f__Parvarchaeaceae;g__Parvarchaeum':0.704871,(GB_GCA_002779595.1:0.320986,GB_GC
A_902385255.1:0.309664)'1.0:o__CG07-land;f__CG07-
land':0.314931)0.978:0.0806575)0.497:0.0419122,GB_GCA_002763265.1:0.72237)0.999:0.0657103)'
1.0:p__Nanoarchaeota;c__Nanoarchaeia':0.0578099,(GB_GCA_002789275.1:1.01115,GB_GCA_0110
49195.1:0.82357)0.445:0.074926)0.995:0.0433331)1.0:0.0553078,(((((((GB_GCA_001563915.1:0.077
4846,GB_GCA_001564035.1:0.0755529)1.0:0.114873,GB_GCA_001564145.1:0.132415)'1.0:g__B1-
Br10-
U2g21':0.144816,(GB_GCA_001761425.1:0.194014,RS_GCF_009617975.1:0.182052)'0.968:g__SG9':0
.0435472)0.925:0.0267863,(GB_GCA_000220375.1:0.291687,RS_GCF_013343275.1:0.172733)0.622:

0.0366728)0.998:0.0347165,(((GB_GCA_001563995.1:0.176763,GB_GCA_014297435.1:0.15311)'1.0:
g__PL-Br10-
U2g16':0.0541217,(GB_GCA_003009795.1:0.0392905,GB_GCA_003009815.1:0.0278524)'1.0:g__SW-
4-43-
9':0.157979)0.986:0.0359524,(GB_GCA_001563875.1:0.109332,GB_GCA_001563905.1:0.0996781)'1.
0:g__B1-Br10-
U2g19':0.100462)1.0:0.0452103)1.0:0.0921253,GB_GCA_000220355.1:0.392016)'1.0:p__Nanohaloar
chaeota;c__Nanosalinia;o__Nanosalinales;f__Nanosalinaceae':0.506807,((((GB_GCA_003660865.1:0.
371211,GB_GCA_013139905.1:0.418459)'0.996:f__B79-
G9':0.0584236,GB_GCA_902384675.1:0.429586)0.982:0.0777115,(GB_GCA_013138765.1:0.154663,
GB_GCA_013138805.1:0.170659)'1.0:f__GLR71;g__GLR71':0.37057)0.999:0.071884,((GB_GCA_0022
54415.1:0.245658,GB_GCA_003660905.1:0.203946)'1.0:f__EX4484-52;g__EX4484-
52':0.469658,GB_GCA_013153555.1:0.608012)0.994:0.0795926)'1.0:p__EX4484-52;c__EX4484-
52;o__EX4484-
52':0.0882774)1.0:0.0609488)0.99:0.0430421,(((((GB_GCA_003553905.1:0.131857,GB_GCA_003554
845.1:0.130922)1.0:0.0493363,GB_GCA_003555465.1:0.142791)1.0:0.0595071,GB_GCA_003550625.
1:0.172331)0.953:0.0455079,GB_GCA_003554235.1:0.144539)'1.0:f__PWEA01;g__PWEA01':0.35058
5,GB_GCA_003663345.1:0.326329)'1.0:p__PWEA01;c__PWEA01;o__PWEA01':0.15414)0.0:0.028794
4,(((((((GB_GCA_004297575.1:0.337868,GB_GCA_014238095.1:0.437731)0.999:0.0524444,bin.598:
0.438306):0.043298,GB_GCA_011363135.1:0.26205)0.363:0.0396764,(GB_GCA_011334385.1:0.2449
78,GB_GCA_011335085.1:0.251477)1.0:0.0720262)'0.992:f__SCSR01':0.0547262,((GB_GCA_0022545
45.1:0.337113,GB_GCA_011333035.1:0.316878)0.273:0.0542413,GB_GCA_011047985.1:0.362758)'1
.0:f__EX4484-
224':0.0698253)1.0:0.0913086,(GB_GCA_001784635.1:0.326879,GB_GCA_002789635.1:0.309634)'1.
0:f__CG10238-14':0.217555)'1.0:o__CG10238-
14':0.101811,(((GB_GCA_003663225.1:0.416103,GB_GCA_003663415.1:0.294303)'0.974:f__QMZP01
':0.0519901,GB_GCA_003663445.1:0.450774)0.193:0.0498011,((GB_GCA_011772645.1:0.355373,GB
_GCA_013151465.1:0.435496)0.716:0.0524264,GB_GCA_003663325.1:0.340688)'1.0:f__QMZT01':0.
0765723)'1.0:o__QMZP01':0.148884)0.969:0.045203,((GB_GCA_000405245.1:0.286116,GB_GCA_01
1773475.1:0.261415)'1.0:f__Aenigmatarchaeaceae':0.127991,GB_GCA_013152905.1:0.439097)'1.0:o
__Aenigmatarchaeales':0.147173)'0.87:p__Aenigmatarchaeota;c__Aenigmatarchaeia':0.0274076)0.9
99:0.0688141,((((GB_GCA_000806115.1:0.16781,GB_GCA_013331845.1:0.178288)'1.0:g__GW2011-
AR5':0.166846,GB_GCA_903825675.1:0.352436)'1.0:f__GW2011-
AR5':0.0996342,GB_GCA_002688965.1:0.514522)1.0:0.128398,GB_GCA_011373045.1:0.540076)'0.9
94:p__Aenigmatarchaeota_A;c__Aenigmatarchaeia_A;o__GW2011-AR5':0.0669493)d__Archaea;
